# The transcriptome analysis of early morphogenesis in *Paracoccidioides brasiliensis *mycelium reveals novel and induced genes potentially associated to the dimorphic process

**DOI:** 10.1186/1471-2180-7-29

**Published:** 2007-04-10

**Authors:** Karinne P Bastos, Alexandre M Bailão, Clayton L Borges, Fabricia P Faria, Maria SS Felipe, Mirelle G Silva, Wellington S Martins, Rogério B Fiúza, Maristela Pereira, Célia MA Soares

**Affiliations:** 1 Laboratório de Biologia Molecular, Instituto de Ciências Biológicas, Universidade Federal de Goiás, 74001-970, Goiânia, Goiás, Brasil; 2 Departamento de Bioquímica e Biologia Molecular, Universidade Federal de Goiás, Brasil; 3 Laboratório de Biologia Molecular, Universidade de Brasília, Brasília, D.F, Brasil; 4 Departamento de Informática, Universidade Católica de Goiás, Goiânia, Goiás, Brasil

## Abstract

**Background:**

*Paracoccidioides brasiliensis *is a human pathogen with a broad distribution in Latin America. The fungus is thermally dimorphic with two distinct forms corresponding to completely different lifestyles. Upon elevation of the temperature to that of the mammalian body, the fungus adopts a yeast-like form that is exclusively associated with its pathogenic lifestyle. We describe expressed sequence tags (ESTs) analysis to assess the expression profile of the mycelium to yeast transition. To identify *P. brasiliensis *differentially expressed sequences during conversion we performed a large-scale comparative analysis between *P. brasiliensis *ESTs identified in the transition transcriptome and databases.

**Results:**

Our analysis was based on 1107 ESTs from a transition cDNA library of *P. brasiliensis*. A total of 639 consensus sequences were assembled. Genes of primary metabolism, energy, protein synthesis and fate, cellular transport, biogenesis of cellular components were represented in the transition cDNA library. A considerable number of genes (7.51%) had not been previously reported for *P. brasiliensis *in public databases. Gene expression analysis using in silico EST subtraction revealed that numerous genes were more expressed during the transition phase when compared to the mycelial ESTs [1]. Classes of differentially expressed sequences were selected for further analysis including: genes related to the synthesis/remodeling of the cell wall/membrane. Thirty four genes from this family were induced. Ten genes related to signal transduction were increased. Twelve genes encoding putative virulence factors manifested increased expression. The in silico approach was validated by northern blot and semi-quantitative RT-PCR.

**Conclusion:**

The developmental program of *P. brasiliensis *is characterized by significant differential positive modulation of the cell wall/membrane related transcripts, and signal transduction proteins, suggesting the related processes important contributors to dimorphism. Also, putative virulence factors are more expressed in the transition process suggesting adaptation to the host of the yeast incoming parasitic phase. Those genes provide ideal candidates for further studies directed at understanding fungal morphogenesis and its regulation.

## Background

*Paracoccidioides brasiliensis *is a dimorphic pathogenic ascomyceteous fungus, endemic to the Latin America that can cause primary disease in humans. In the soil the fungus grows as saprobic mycelium, resulting in the formation of propagules, which initiates the infection in humans when inhaled into the respiratory tract. Subsequently, in the lung, the mycelia propagules develop into yeast cells. The mycelium to yeast transition can be replicated in vitro by growing mycelia in conditions of elevated temperature. The ability of *P. brasiliensis *to grow in the mycelia form in the soil and shift to the yeast form in the host is important for infection and disease. Once introduced into the host, the mycelial propagules have to convert to yeasts, a condition essential for the fungus to survive and proliferate [2,3].

The morphological transition in *P. brasiliensis *is governed predominantly by the temperature and is preceded by several molecular changes. The identification of genes specifically involved in the mycelium to yeast transition in *P. brasiliensis *has been subject of great interest, since pathogenicity is intimately linked to the dimorphic transition in some fungi [4]. Approaches used in the identification of genes important for the transition process include, for example, the differential expression of *P. brasiliensis *genes in both fungal phases identified by electronic subtraction and cDNA microarray hybridization, which were employed to search for genes whose expression, displayed statistically significant modulation during the mycelium to yeast transition [5-8].

The biochemical processes that control the morphogenesis of *P. brasiliensis *are just coming to light. The dimorphic transition involves alterations in the cell wall composition and in the structure of carbohydrates polymers [9,10]. The yeast cells exhibit an energy metabolism biased towards ethanol production through fermentation, whereas mycelium metabolism tends to be more aerobic than that of yeast cells. Also the glyoxylate pathway is more active in the yeast form of *P. brasiliensis *[5]. Hyper expression of some enzymes in the sulphur metabolism pathway in the yeast phase of *P. brasiliensis*, as well as during the transition from mycelium to yeast have been reported, corroborating previous descriptions of the importance of this metabolic pathway to the dimorphic process [6,8,11].

Here, we have tested the concept that novel genes involved in *P. brasiliensis *phase transition could be described by applying a transcriptome analysis of cells undergoing mycelium to yeast transition. In this manuscript we describe EST analysis to assess the expression profile of mycelium undergoing yeast transition. This choice of approach distinguishes the present work from previous recently published papers that employed microarray hybridization, electronic subtraction and suppressive subtraction hybridization in order to assess differences using differentiated yeast and mycelium cells [5-8,12]. Using a custom analysis pipeline for sequences of *P. brasiliensis*, isolate *Pb*01, yeast and mycelium forms [1] we obtained an EST databank web interface [13].

In this study we report the in silico analyses and comparison of ESTs from mycelium undergoing the early transition to yeast with mycelium differentiated cells. Our analysis revealed 179 genes that are positively modulated during the early transition process, when compared to mycelia. Additionally 48 novel genes were described in the *P. brasiliensis *transition cDNA library. Upon categorization by known databases we have selected MIPS (Munich Center for Protein Sequences) categories for further analyses. Several ESTs were selected for semi-quantitative and quantitative analysis to examine changes in gene expression induced by the temperature induced transition of phases.

## Results and Discussion

### cDNA library construction, sequencing and sequence annotation

Transcriptome profiling of mycelium undergoing differentiation to yeast cells in *P. brasiliensis *has directed our studies to reveal several uncharacterized genes involved in this process. We performed in this EST-based program the sequencing 2880 randomly selected clones. Of these, 2666 gave readable sequences. 1107 sequences remained after vector and low quality sequences were removed. Of these, 166 consisted of singletons and 473 corresponded to consensus with two or more ESTs. In total, 447761 bp of assembled sequences were obtained corresponding to an average consensus sequence length of 404 bp. The 1107 sequences were annotated. A total of 828 sequences (74.8%) showed significant similarity to known protein sequences (E value ≤ 10^-4^) based on BLAST searches and 433 ESTs (39.1%) had unknown function and were classified as hypothetical proteins. 992 sequences (89.6%) gave significant hits to ESTs present in the *P. brasiliensis *transcriptome database [1] or in the GenBank database. In addition, 115 sequences (10.4% of the total) represented novel genes of *P. brasiliensis*.

### Description of the ESTs in the transition transcriptome

**Figure 1:**
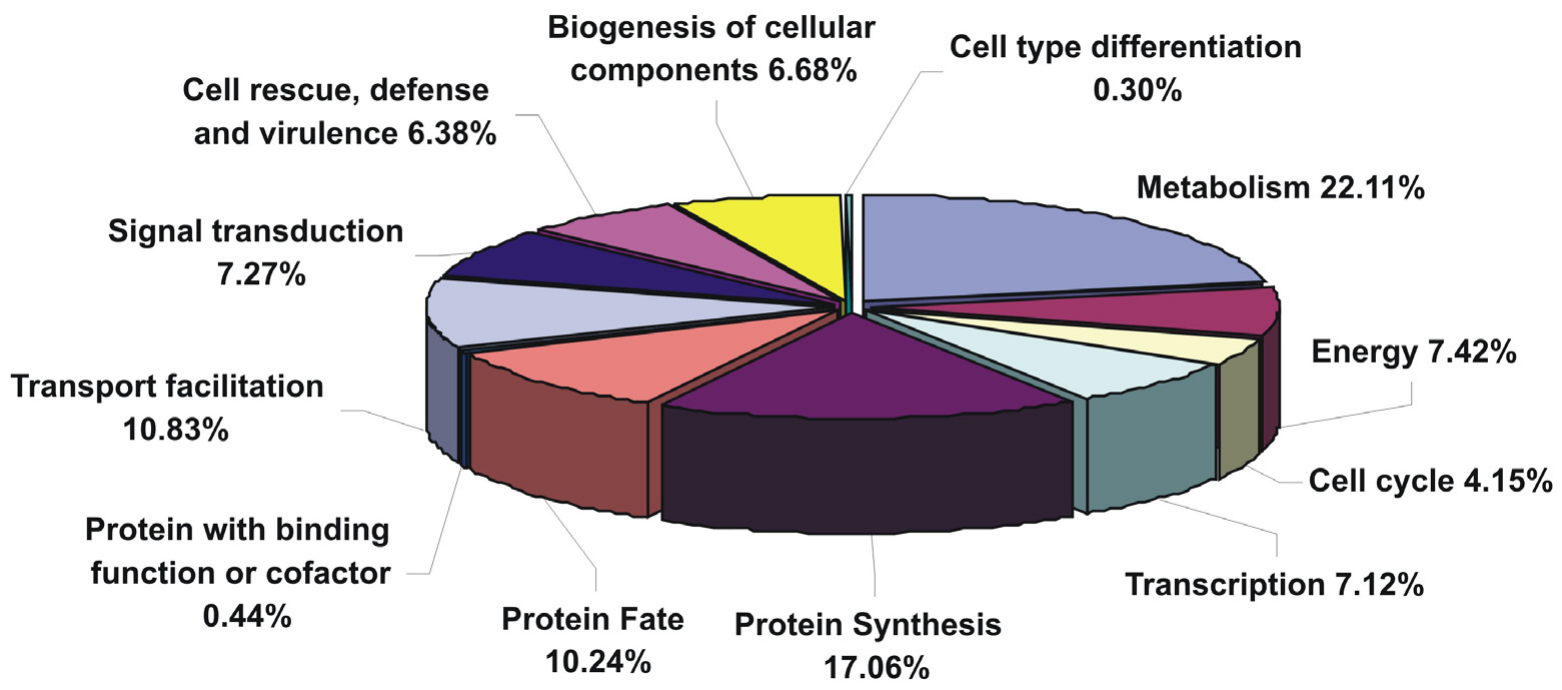
**Classification of ESTs from the transition cDNA library of *P. brasiliensis***. The classification was based on *E *value and performed according to the functional categories developed on the MIPS functional annotation scheme.

An overview of the probable adaptations made by *P. brasiliensis *mycelium during morphogenesis can be obtained by analyzing the ESTs in this early stage of cellular differentiation. As shown in Fig. 1, the ESTs were mainly represented as following: a total of 22.11% of the annotated ESTs corresponded to the fungal metabolism; 17.06% of the ESTs were related to the protein synthesis machinery; 10.83% of the transcripts corresponded to homologues encoding transport facilitators; 10.24% corresponded to ESTs related to protein fate; 7.42% to energy; 7.27% to signal transduction proteins; 7.12% were related to the transcription machinery; 6.68% corresponded to transcripts related to the biogenesis of cellular components; 6.38% corresponded to ESTs encoding cell rescue, defense and virulence factors.

### Comparison of *P. brasiliensis *ESTs present in the transition library to those described for yeast and mycelium stage specific phases: induced genes identified by in silico EST subtraction

We attempted to determine the putative function of the set of 639 phrap unisequences by searching for homologs in the GenBank non-redundant protein database using BLAST X. We also compared the sequenced ESTs present in the transition library to those present in the mycelium transcriptome database. According to the subtractive analysis, the classification of induced genes was designed for the ESTs that were not previously described in *P. brasiliensis *in databases or that manifested increased expression in the transition library as compared to mycelia transcriptome database [1]. This classification was performed according to the statistical test described by Audic and Claverie [14], with a 99% confidence rate. The comparative analysis of all the ESTs annotated in the transition library is available in Table 1, supplementary material. From the 1107 ESTs identified in this work, 426 of the total corresponded to induced genes in the transition library. From the 426 annotated ESTs, 115 corresponded to novel ESTs, representing 48 novel classified genes. Table 2, supplementary material, summarizes the results of such comparison. As shown, the majority of transition induced genes (82.12%) was composed of unique sequences or groups of two or three ESTs. Genes with altered expression included those involved in metabolism of amino acids, nitrogen, sulfur, nucleotides, carbohydrates, vitamins and lipids. In addition genes related to energy generation, signal transduction and cell wall biogenesis, were increased. A small subset of genes with elevated expression had unknown function. The largest induced groups of sequences consisted of a total of 24 ESTs with homology to a histidine protein kinase sensor for GlnG regulator, 18 ESTs exhibiting homology to ubiquinone/menaquinone methyltransferase, 11 ESTs with homology to arylsulfatase regulatory protein, 09 ESTs with homology to acidic amino acid permease, 06 ESTs with homology to a HSP 90 and 07 ESTs with homology to aspartyl protease.

Genes involved in sulfur assimilation, have been described as induced in *P. brasiliensis *transition from mycelium to yeast and in yeast differentiated cells [6,8]. Here, we described in the transition transcriptome the induction of a set of genes related to sulphur metabolism, such as, the transcript encoding sulfite reductase (E.C. 1.8.1.2) an enzyme of the sulfur assimilation pathway, leading to cysteine biosynthesis. Sulfite reductase contains a special acidic heme group called siroheme. One of the novel genes detected in the transiton library encodes for an urophorphyrinogen III methylase (E.C 2.1.1.107) homologue to the Met1p of *Saccharomyces cerevisiae*, related to the sirohaem and cobalamin biosynthesis [15,16]. Also, the transcript encoding sulfate permease was induced compared to the mycelia transcriptome. Sulfate is co-transported into the cells in an energy dependent process catalyzed by specific plasma membrane permeases [17]. An arylsufatase regulatory protein probably involved in the regulation of sulfatase genes was described in the transition transcriptome. The transcript in *P. brasiliensis *has sequence identity to bacterial and fungal arylsulfatases regulatory proteins. Sulfatases catalyze hydrolytic cleavage of sulfate ester bonds, liberating sulfate and the corresponding alcohol [18]. In *Neurospora crassa *arylsulfatase is up regulated by sulfur starvation and appears to function as a mechanism for sulfur scavenging [19]. Also, a thiosulfate sulphurtransferase (TST) (E.C. 2.8.1.1) putatively, a mitochondrial matrix protein that plays roles in formation of iron sulfur proteins, as well as in modification of iron-sulfur proteins [20] was induced in the transition transcriptome. The increase in the expression of genes related to the sulphur metabolism, including the description of novel transcripts corroborates the previous descriptions of the involvement of sulphur metabolism in the transition process of *P. brasiliensis *[6,8,11].

The list of induced genes also includes several ESTs encoding proteins related to lipid metabolism, to signal transduction and to carbohydrate metabolism that will be referred below. Also proteases, such the Lon protease putatively related to degradation of damaged or nonnative proteins in the mitochondrial matrix are induced [21]. An aspartyl protease and a zinc metalloprotease were among the transcripts with increased expression. Of special note molecules related to protein fate, such as to glycoslylation and degradation, are abundant in the transition transcriptome, as shown in Table 2, supplementary material.

### An overview of genes related to the membrane/cell wall remodeling presenting increased expression in the transition library

We catalogued the ESTs potentially associated with fungal cell wall/membrane synthesis/remodeling described during the mycelium to yeast transition. Table 1 depicts the ESTs predominantly related to the synthesis of those components. The transcripts with increased expression include those encoding enzymes related to the cell wall carbohydrates biosynthesis and degradation, the transporters of the precursors for the synthesis of such molecules, enzymes related to protein glycosylation and to the synthesis of membrane lipids.

It is presumed that the dimorphic transition occurs simultaneously with changes in the fungal cell wall composition of such compounds as phospholipids and carbohydrate polymers [3,10,22]. In *P. brasiliensis*, lipids, chitin, glucans and proteins are the main constituents of the cell wall in mycelium and yeast cells. The transition transcriptome data suggest that *P. brasiliensis *favors the membrane and cell wall remodeling in the early stages of transition, from mycelium to yeast. Transcription of 34 cell wall/membrane related genes were induced upon temperature shift (Table 1).

**Figure 2:**
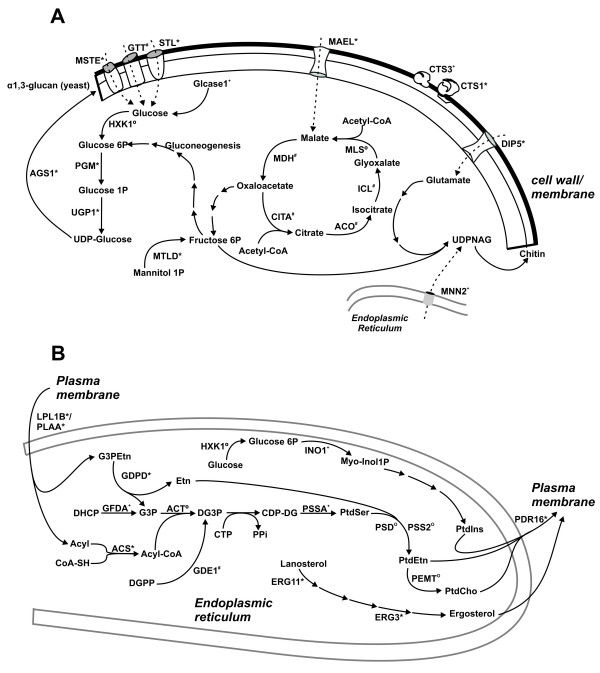
**The synthesis of the cell wall components from glucose and lipids**. Induced transcripts (*), novel transcripts (+), transcripts detected in the transition transcriptome without induction (#) and transcripts present at public databases (o). A – Some steps in the synthesis of glucan and chitin. GLCase 1: Alpha-glucosidase 1; HXK1: hexokinase; PGM: phosphoglucomutase; UGP1: uridine diphosphate glucose pyrophosphorylase; AGS1: alpha glucan synthase; MTLD: mannitol-1-phosphate dehydrogenase; MSTE: monosaccharide transport protein; GTT: glucose transporter protein; STL: sugar transporter protein; CTS 1: chitinase 1; CTS 3: chitinase 3; DIP 5: acidic amino acid permease; MAEL: malate permease; MDH: malate dehydrogenase; CITA: citrate synthase; ACO: aconitase; ICL: isocytrate lyase; MLS: malate synthase; UDPNAG: uridine diphosphate N acetylglucosamine; MNN2: UDPNAG transporter. B – The synthesis of some lipids from the cell membrane. LPL1B: Lysophospholipase; PLAA: phospholipase A2; DHCP: dihydroxycetone phosphate; GFDA: glycerol 3 phosphate dehydrogenase; G3P: glycerol 3 phosphate; G3PEtn: Phosphatidyl ethanolamine; GDPD: glycerophosphodiester phosphodiesterase; ACT: acyltransferase; ACS: acyl-coenzyme A synthethase; Acyl-CoA: acyl-coenzyme A; DGPP: diacylglycerol pyrophosphate; GDE1: diacylglycerol pyrophosphate phosphatase; DG3P: diacylglycerol 3 phosphate; CTP: cytidine triphosphate; PPi: pyrophosphate; CDP-DG: cytidine diphosphate diacylglycerol; PSSA: phosphatidylserine synthase; PtdSer: phosphatidylserine; PSS2: phosphatidylethanolamine serine transferase; PSD: phosphatidylserine decarboxylase; PtdEtn: phosphatidylethanolamine; PEMT: phosphatidylethanolamine metyltransferase; PtdCho: phosphatidylcholine; INO1: myo-inositol 1 phosphate synthase; Myo-Inol1P: myo-inositol 1phosphate; PtdIns: phosphatidylinositol; PDR16: phosphatidylinositol transfer protein; ERG 11: Lanosterol 14-alpha demetylase; ERG 3: sterol delta 5,6-desaturase.

In Table 1 and Fig. 2A, an overview of the induced enzymes and transporters putatively related to the biosynthesis of the carbohydrate compounds of the cell wall, is shown.

Many cell wall-related proteins were found among the presently identified ESTs, including molecules related to the chitin synthesis, alpha glucan synthesis and chitin degradation. The main polysaccharide of the yeast cell wall is alpha-glucan, whereas the mycelium contains predominantly beta-glucan [23]. Several genes related to the synthesis of the carbohydrate components of the cell wall were induced in the transition library, in comparison to the mycelium transcriptome database [1]. Those genes include phosphoglucomutase (*pgm*) UDP-Glucose pyrophosphorylase (*ugp1*), and alpha -1,3 glucan synthase (*ags1*), (Table 1, Fig. 2A), putatively enabling the increase in the synthesis of alpha-1,3 glucan in the yeast incoming cell wall [10]. A novel transcript encoding an alpha glucosidase 1 (GLCase I) was described. It has been suggested that glucosidases are directly involved in the synthesis or processing of beta-1,6 glucan in *S. cerevisiae *[24].

Chitin is the major component of yeast cells in which it comprises (37% to 48%) of the total cell wall components. Of special note is the detection of a novel transcript encoding an UDP-N-acetyl glucosamine transporter (MNN2), which has been described in *S. cerevisiae*. The cytoplasm is the sole site of sugar nucleotide synthesis and sugar nucleotides must be transported into various organelles in which they are utilized as a donor substrate for sugar chain synthesis. It has been demonstrated that UDP-N-acetyl glucosamine transporter encoded by the YEA4 gene in *S. cerevisiae *is located in the endoplasmic reticulum and is involved in cell wall chitin synthesis in this fungi [25]. GDA1 (guanosine diphosphatase) generates both GMP and UMP required as antiporters for guanosine and uridine sugar transport into the Golgi lumen. Deleted strains of *Kluveromyces lactis *for *gda1 *present altered cell wall stability and composition [26]. Chitinase 1 (CTS1) and 3 (CTS3), the latter a novel gene, were induced in the transition library suggesting their role in the remodeling of the cell wall and providing N-acetyl glucosamine for the synthesis of chitin. The DIP5 encoding transcript (acidic amino acid permease) was increased in the transition library and could provide the uptake of glutamate, a precursor required for the synthesis of chitin. We recently described that this transcript is up regulated in *P. brasiliensis *yeast cells during incubation in human blood and is hypothetically related to the cell wall remodeling supposed to occur during osmotic stress [27]. In addition, the induced enzyme HPAT (histidinol phosphate aminotransferase) could also provide glutamate for the synthesis of chitin precursors.

Sugar transporters MSTE (monosaccharide transport protein), STL (sugar transport protein), GTT (glucose transporter) were present in the transition transcriptome; the first two genes were present as increased transcripts. The increased expression may permit the fungus to increase uptake of carbohydrates, thus accelerating the synthesis of glucan and chitin (Table 1, Fig. 2A). The *mael *(malate permease) cDNA encoding the transporter for malate is an induced gene in the transition library and could provide the precursor for gluconeogenesis furnishing carbohydrate precursors to the cell wall components biosynthesis. Also the availability of compounds to the glyoxalate cycle seems to be favored during transition. The MAEL (malate transporter) could provide malate for the glyoxylate cycle. The enzymes (CITA) citrate synthase (E.C.2.3.3.1), (ACO) aconitase (E.C.4.2.1.3), (ICL) isocitrate lyase (E.C.4.1.3.1), and (MDH) malate dehydrogenase (E.C.1.1.1.37) were present in the transition library, indicating that the glyoxalate cycle is functional during the transition from mycelium to yeast. Of note the transcriptome analysis in *P. brasiliensis *showed several pathways that provide substrates for the glyoxalate cycle that is up regulated in the yeast cell, as described previously [5].

Induced transcripts in the transition library also involve those related to the phospholipids synthesis, as well as to ergosterol, as shown in Table 1 and Fig. 2B. The enzyme GFDA (glycerol 3P dehydrogenase) converts DHCP (dihydroxycetona phosphate) in G3P (glycerol 3P). The *gfdA *null mutant of *Aspergillus nidulans *displays reduced G3P levels and an osmoremediable growth defect, which is associated with abnormal hyphal morphology [28]. G3P can be produced by the action of the enzyme GDPD (glycerophosphodiester phosphodiesterase) which promotes the hydrolysis of phosphatidylethanolamine (G3PEtn). Both enzymes are induced in the transition from mycelium to yeast cells, as shown in Table 1 and Fig. 2B. The ACT (acyltransferase) promotes the addition of acyl groups to G3P generating DG3P (diacylglycerol 3P); this enzyme is described in *P. brasiliensis *in the public databases. The acyl CoA required for the synthesis of DG3P is produced by ACS (acyl-CoA synthetase) which can utilize an acyl group that can be liberated by the action of phospholipases A and B (PLAA LPB1B and respectively); all the ESTs encoding those enzymes are induced in the transition from mycelium to yeast, as described in Fig. 2B and Table 1. Also, DG3P can be produced by GDE1 (diacylgycerol pyrophosphate phosphatase). CDP-diacylglycerol (CDP-DG) produced from DG3P is the precursor of phospholipids. PSSA (phosphatidylserine synthase) produce phosphatidylserine from CDP-DG, and is a novel transcript described in the present work. The induced transcript of INO1 (myo-inositol-1-phoshate synthase), produces myo-inositol 1P the precursor for the synthesis of phosphatidylinositol. The PDR16 (phosphatidylinositol transfer protein), also induced, transports phospholipids from their site of synthesis in the endoplasmic reticulum to the plasma membrane [29].

Polyunsaturated fatty acids (UFA) are major components of the membranes and are produced from monounsaturated fatty acids by several fatty acid desaturases in many fungi. DESA (fatty acid desaturase) was demonstrated to be induced in the transition library suggesting active membrane remodeling during the morphogenetic event in *P. brasiliensis*. The synthesis of ergosterol seems also to be induced during the transition process. ERG 11 (lanosterol 14-alpha demetylase) and ERG 3 (sterol delta 5, 6-desaturase) present transcripts induced in the transiton library (Fig. 2B, Table 1).

### An overview of induced genes putatively related to signal transduction

We also identified a variety of signal transduction systems in *P. brasiliensis *ongoing differentiation to yeast cells, such as MAPK, serine/threonine protein kinases, signal histidine kinases and two component sensor kinases. The most increased transcript encodes for a histidine protein kinase sensor for GlnG regulator, which presented 24 ESTs in the transition library (Table 2, supplementary material and Table 3, supplementary material). Novel genes were also those encoding for a two-component sensor kinase (06 ESTs), calcineurin subunit b (02 ESTs), UVSB phosphatidylinositol-3-kinase (01 EST), forkhead associated protein (01 EST), Rho GTPAse activating protein (01 EST).

Histidine kinases are signaling transduction proteins that organisms in all three domains of life use to respond to environmental signals and control developmental process [30,31]. *S. cerevisiae *has a single hybrid histidine kinase, sln1p, which regulates an osmosensing mitogen-activated protein kinase (MAPK) cascade, an oxidative stress-response pathway, and cell wall biosynthesis [32,33]. *Blastomyces dermatitidis *DRK1 (for dimorphism-regulating histidine kinase) is a conserved hybrid histidine kinase that is indispensable for dimorphism, virulence and pathogenicity [34]. The ESTs encoding the putative histidine kinase induced in the transition library presents some structure domains and sequence of histidine kinase, such as the histidine-containing H-box and an aspartate-containing D-box (data not shown).

The fungal cell wall is an essential cellular boundary that controls many cellular processes. It allows cells to withstand turgor pressure preventing cell lysis. In *S. cerevisiae *a MAPK cascade which is essential in transducing signals to adapt cell wall biosynthesis under a variety of environmental conditions, is activated by the protein kinase C, constituting the PKC cell integrity pathway [35]. A MAPK and PKC proteins were induced in the transition library suggesting their involvement in the cell wall biosynthesis. In addition, calcineurin has been proposed as essential for survival during membrane stress in *Candida albicans *[36]. Also a FHA (forkhead associated) protein and an UVSB phosphatidylinositol-3-kinase were increased in the transition library suggesting the requirement of DNA damage checkpoint kinases in the dimorphic transition of *P. brasiliensis *[37,38].

In *P. brasiliensis *transition transcriptome it was detected 53 ESTs (4.78% of the total ESTS) encoding for potential signal transduction proteins (see Table 3, supplementary material). From those, 10 are induced transcripts comprehending 06 novel genes, suggesting that the morphological transition in *P. brasiliensis *is mediated by a series of signal transduction systems that control the adaptation to the environment to the fungus survive and proliferate within the host.

### Novel genes of *P. brasiliensis *detected in the transition library

Table 2 summarizes the transcripts detected in the transition library that were not present in the *P. brasiliensis *transcriptome [1] or in public databases. A total of 48 novel genes are reported here. Several enzymes related to the general metabolism were described as novel genes. As examples, the orotate phosphoribosyltransferase (URA5) (E.C.2.4.2.10) was present in the transition library. Also a phosphatidylserine synthase (E.C.2.7.8.8) putatively related to the metabolism of phospholipids, as cited above. Enzymes related to protein modification, transport facilitators and signal transduction were also detected as novel genes in the transition library and were discussed before.

A novel transcript encodes for a homologue of SamB, related to morphogenesis in ascomycetous fungi [39]. We exploited sequence data to examine the presence of the conserved Zn-finger like domain in the deduced homolog of *P. brasiliensis *(data not shown). It was observed the high conservation of the Zn finger-like domain in SamB, crucial for fungal morphogenesis, as described [39].

### Putative virulence factors

Expression analysis can be a valuable first step in virulence genes discovery. Putative virulence factors were selected on basis with homology in other pathogenic microrganisms. With these criteria, we classified 12 induced genes as putative virulence factors of *P. brasiliensis*. Table 3 presents some induced genes, potential virulence factors in *P. brasiliensis*. AGS1 was catalogued as a potential virulence factor, since in *Histoplasma capsulatum *the reduction of its activity by RNA interference or allelic replacement leads to reduction in the fungal ability to colonize lung [40]. Mutants of *Aspergillus fumigatus *in glucanosyltransferases 1 and 2 (gel 1 and 2) have abnormal cell wall compositon and conidiogenesis and reduced virulence in a murine model of invasive aspergillosis, suggesting that beta(1–3) glucanosyltransfease activity is required for both morphogenesis and virulence in this fungal pathogen [41]. Calcineurin plays a global role in stress responses necessary for fungal cell survival and in this sense can be defined as a virulence factor [42]. Deleted para-aminobenzoic acid synthetase (paba) strains of *A. fumigatus *present complete inability in causing lethal infection in mice [43]. We previously described that the catalase P (CAT P) presents canonical motifs of monofunctional typical catalases, as well as the peroxisome PTS-1 targeting signal and its expression was induced in cells treated with H_2_O_2_, suggesting its involvement in protecting *P. brasiliensis *yeast cells against exogenously produced peroxides [44]. Secreted products are a common means by which fungi can promote virulence [45,46]. The aspartyl proteinase (ASP) described in Table 3 is putatively a secreted protease that may facilitate tissue invasion; the same could be hypothesized to the transcript encoding a zinc metalloprotease [46]. Phospholipases are critical for modification and redistribution of lipid substrates, membrane remodeling and microbial virulence. The null mutants and revertant strains for a phospholipase B gene of *C. albicans *present reduced phospolipase A2 activity and attenuated virulence [47]. In addition an inositol phosphosphingolipid phospholipase C (PLC) gene of *C. neoformans *promotes neurotropism of *C. neoformans *depending on the immune status of the host by protecting the fungus from the hostile intracellular environment of phagocytes [48].

Specific adhesins can enable fungal cells to adhere to host cells or the ECM components. We previously demonstrated that he fungal glyceraldehyde 3-phosphate dehydrogenase (GAPDH) is a potential virulence factor of *P. brasiliensis*, since it can diminish the fungus yeast cells ability to adhere and invade in vitro cultured pneumocytes [49]. Also the mannosyltation of proteins can be related to virulence. The mnn5 mutant of *C. albicans *exhibited attenuated virulence in mice [50]. The transcripts encoding for a hemolysin like protein of *Candida glabrata *(HLP) and for urease (URE), are possible virulence factors (Table 3). Switching in *C. glabrata *which may provide colonizing populations for rapid response to the changing physiology of the host regulates the hlp expression [51]. Urease which catalyzes the conversion of urea into ammonia is described to contribute to alkalinity at the sites of fungal infection, causing a great damage to the host tissues [52]. Of special note, the up regulation of those potential virulence factors in the transition of mycelium to yeast cells suggests the fungal adaptation to the new conditions to be faced in the host milieu.

### Expression profile

**Figure 3:**
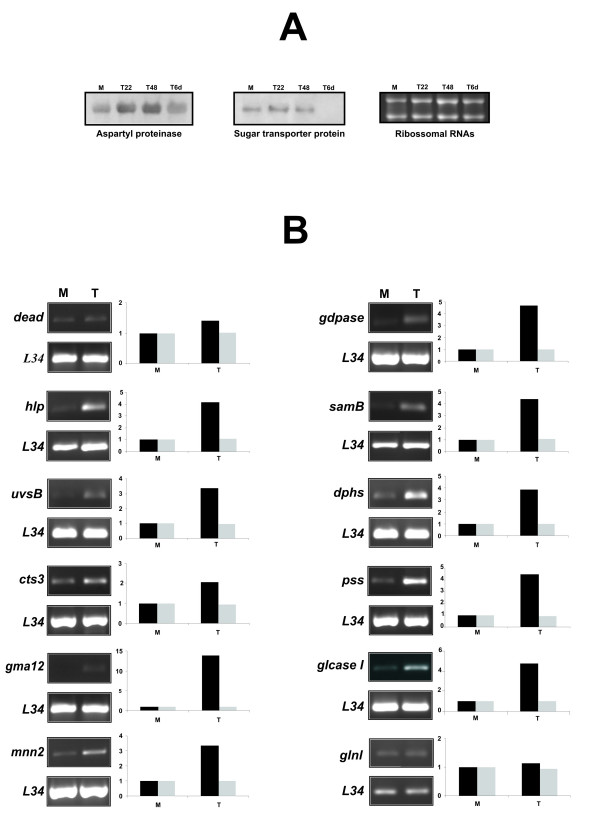
**Validation of the classification of induced transcripts in the transition library**. A – Analysis by northern blot was carried out with RNA from mycelium during transition to yeast colleted at 22 h, 48 h and 6 days after the temperature shift. Total RNA was fractionated on a 1.2% formaldehyde agarose gel and hybridized to the cDNA inserts Aspartyl proteinase (*asp*) and Sugar transporter protein (*stl*). Ribosomal RNAs are shown as the loading control. The sizes of the transcripts are as follows: *asp *1.7 kb; *stl *2.65 kb. B – Validation of some novel genes of *P. brasiliensis*. Semi-quantitative RT-PCR of RNAs obtained from mycelium in transition to yeast. Semi-quantitative RT-PCR analysis was carried out with specific primers, as described. Gray bars indicate the transcript level for the L34 ribosomal protein and black bars refers to the described new transcript. Numbers associated with the bars indicate fold differences relative to the data for the reference mycelium, which were established by densitometry analysis. Using varied number of cycle numbers, the exponential phase of each primer was determined and used to allow semi-quantitative analysis of the respective reactions. The same amount of cDNA was used for all PCRs. The RNAs used for RT-PCR were obtained from samples of: mycelium (M) and mycelium in transition to yeast after 22 h of the temperature shift (T). Genes and sizes of the respective amplified fragments are as follows in bp: *dead*: 408; *hlp*: 274; *uvsB*: 318; *cts3*: 268; *gma12*: 152; *mnn2*: 363; *gdpase*: 126; *samB*: 114; *dphs*: 284; *pss*: 281; *glcaseI*: 359; *glnl*: 368.

We validated the classification of induced transcripts by northern blot analysis, as shown in Figure 3A. The transcripts encoding aspartyl proteinase and sugar transporter protein, were classified as induced in the transition library by electronic northern and according to our experimental northern blot data, were accumulated in mycelium during transition to yeast cells. It has to be emphasized that the in silico analysis of the ESTs redundancy revealed for the transcripts encoding aspartyl protease and sugar transporter protein, 3 ESTs in the mycelium transcriptome database for both; 7 and 5 in the present transition library, respectively and 3 for both, ESTs in the yeast transcriptome database. We also validated 12 novel genes identified in the transition cDNA library, by semi-quantitative RT-PCR, and their expression profiles are shown in Figure 3B. All transcripts were induced upon transition, as demonstrated.

## Conclusion

The 1107 ESTs identified in this study represent the first effort to define the *P. brasiliensis *genes present in a cDNA library of the fungal RNA obtained during the transition from mycelium to yeast. These data increase the number of identified *P. brasiliensis *genes induced during the transition. Annotation of the unisequences revealed that 992 (89.6%) had homologues in the *P. brasiliensis *public databases, and therefore about 115 (10.4%) represent novel genes. Annotation of the ESTs revealed a great repertoire of genes that could function in cell wall/membrane remodeling during the transition process. Also, putative virulence factors, novel transduction signal proteins, novel enzymes related to sulphur metabolism, among others, had been described. Overall these data can help in accelerating research on this important human fungal pathogen.

## Methods

### Fungal isolate, growth conditions and induction of mycelium to yeast transition

*P. brasiliensis*, isolate *Pb*01 (ATCC-MYA-826), has been studied at our laboratory. It was grown in Fava-Netto's medium [1% (w/v) peptone; 0.5% (w/v) yeast extract; 0.3% (w/v) proteose peptone; 0.5% (w/v) beef extract; 0.5% (w/v) NaCl; 4% (w/v) agar, pH 7.2], at 22°C, as mycelium. The differentiation was performed in liquid medium (Fava-Netto's medium) by changing the culture temperature from 22°C to 36°C for the mycelium to yeast transition, as we previously described [44]. The cells were previously grown in liquid medium for 18 h before changing the incubation temperature, which was maintained for 22 h.

### RNA extraction and preparation of the cDNA library

Total RNA was purified from *P. brasiliensis *mycelium in transition to yeast cells (see above) using TRIZOL (GIBCO™, Invitrogen, Carlsbard, CA). The mRNA was purified by using the Poly (A) Quick ^R ^mRNA isolation kit (Stratagene, La Jola, CA). The cDNA library was constructed in the unidirectional pCMV.SPORT 6 (Invitrogen) according to the manufacturer's instructions, exploiting the *Not *I and *Sal *I restriction sites. The cDNA library was not normalized, i.e., no attempt was made to reduce the redundancy of highly expressed transcripts.

### Plasmid isolation and DNA sequencing of the cDNA library

Plasmids constructs were transformed into *Escherichia coli *ElectroMAX™ DH10B cells (Invitrogen). The cDNA library was plated to approximately 200 colonies per plate (150 mm Petri dish). The colonies were randomly selected and transferred to a 96-well polypropylene plate containing LB medium and grown overnight. Plasmid DNA was isolated and purified using Millipore filters (MilliPore^®^). cDNA inserts were sequenced from the 5' end by employing standard fluorescence labeling DYE namic™ ET dye terminator kit with the M13 flanking vector primer. Samples were loaded onto a MegaBACE 1000 DNA sequencer (GE Healthcare, Amersham Biosciences), for automated sequence analysis.

### EST Processing Pipeline, Annotation and Sequence Analysis

The resulting electropherograms were transferred to the server where the pre-processing took place. ESTs were screened for vector sequences against the UniVec data. The sequences were assembled by using the PHRED/PHRAP/CONSED [53]. EST sequences were pre-processed using the Phred [54] and Crossmatch [55] programs. Only sequences with at least 100 nucleotides and Phred quality greater or equal to 20 were considered for further analysis. A total of 1107 ESTs were selected by these inclusion criteria. The resulting sequences were uploaded to a relational database (MySQL) on a Linux (Fedora Core 3) platform, and processed using a modified version of the PHOREST tool [56]. We modified PHOREST to the assembling of the sequences using the CAP [57] and store the BLAST results of many databases including GenBank non-redundant (nr) database, Cluster of Orthologus Groups (COG), Gene Ontology (GO), MIPS [58], KEGG [59] and some fungi specific databases. In addition, an option to automatically translate EST sequences and compare their frames against the InterPro database [60] was implemented. These modifications allowed easy identification of homolog sequences, as well as the identification of domains and functional sites, which improved the manual annotation process. Similarities with E-values ≤ 10^-4 ^were considered significant. For comparative analysis the ESTs were grouped in 639 clusters, represented by 166 contigs and 473 singlets. The clusters were compared with *P. brasiliensis *transcriptome database [1] and public databases to identify new transcripts, by using the BLAST program [61]. The ESTs had been submitted to GenBank, under accession numbers EH040628 to EH041734.

### In silico determination of induced genes in the mycelium to yeast transition by electronic northern

To assign a differential expression character, the contigs formed with mycelium and the transition ESTs were statistically evaluated using the Audic and Claverie's method [14]. It were considered induced genes in the transition library those that were not previously described in the mycelium transcriptome database [1], as well as those more expressed as determined with a 99% confidence rate. A web site [62] was used to compute the probability of differential regulation.

### Northern blot

Northern hybridization was performed with 10 μg of total RNA fractioned on a 1.2% agarose-formaldehyde denaturing gel and transferred to a Hybond-N+ nylon membrane (GE Healthcare). The RNAs, corresponding to different times of cellular differentiation, were hybridized to the correspondent cDNA probes in Rapid-hyb buffer (GE Healthcare) and washed according to the manufacturer's instructions. Probes were radiolabeled by using Rediprime II Random Prime labeling System (GE Healthcare).

### Semi-quantitative RT-PCR analysis (sqRT-PCR)

Semi-quantitative RT-PCR was performed for 12 genes to confirm the presence of new transcripts. Total RNA was extracted from *P. brasiliensis *mycelium in transition to yeast after 22 h of the temperature shift from 22°C to 36°C, as described. RNAs used for sqRT-PCR were from independent experiments from those used in the cDNA library construction. cDNAs were synthesized by reverse transcription using the Superscript II RNAse H-reverse transcriptase (Invitrogen™, Life Technologies). cDNAs were used for PCR in 30 μl reaction mixture containing specific primers, sense and antisense, as described in Table 4. PCR conditions were: 25–35 cycles at 95°C for 1 min; annealing at 55–65°C for 2 min; 72°C for 1 min. The annealing temperature and the number of PCR cycles were optimized for each experimental condition to ensure linear phase of amplification. Amplicons were analyzed by agarose gels electrophoresis (1%). The analyses of relative differences were performed by using Scion Image Beta 4.03 program [63].

## Authors' contributions

KPB prepared the cDNA library, performed the DNA sequencing, the validation experiments, contributed to gene ontology classification and supported the preparation of the figures and tables. AMB contributed to the construction of the cDNA library, to the classification of gene ontology terms, to the data analysis and to the preparation of the manuscript. CLB contributed to the culture of the fungus, to the construction of the cDNA library, to the classification of gene ontology terms and to the manuscript edition. FPF contributed to the construction of the cDNA library. MSSF contributed to the results discussion and to the manuscript preparation. MGS contributed to the DNA sequencing and to the classification of gene ontology terms. WSM and RBF analyzed the raw sequences and contributed to the construction of the EST database. MP contributed to the analysis of the raw sequences and to the preparation of the manuscript. CMAS designed the project and the database, contributed to the data analysis and to the preparation of the manuscript. All authors read and approved the final manuscript.

## Supplementary Material

Additional File 1 ***P. brasiliensis *clusters annotated in the cDNA library**. Table representing the annotated clusters that were generated by sequencing of the cDNA clones. For each cluster the table includes: the function as assigned by BLAST-based similarity, the BLAST subject species, the GenBank ID for the BLAST subject used for functional assignment and the Expect value obtained with each unisequence, the redundancy in the transition library and in the mycelium transcriptome database. Click here for file

Additional File 2 **Induced *P. brasiliensis *ESTs and novel genes generated from the transition library**. Table representing the annotated clusters that were generated by sequencing of the cDNA clones. For each cluster the table includes: the function as assigned by BLAST-based similarity, the BLAST subject species, the GenBank ID for the BLAST subject used for functional assignment and the Expect value obtained with each unisequence, the redundancy in the transition library and in the mycelium transcriptome database. Click here for file

Additional File 3 ***P. brasiliensis *induced transcripts potentially related to signal transduction**. Table representing the annotated clusters that were generated by sequencing of the cDNA clones of the transition library. For each cluster the table includes: the function as assigned by BLAST-based similarity, the redundancy in the transition library and in the mycelium transcriptome database. Click here for file

## Figures and Tables

**Table 1:** Induced *P. brasiliensis *transcripts potentially related to membrane and cell wall synthesis/remodeling.

**Gene Product**	**E.C. number**	**Annotated function**	**Predicted redundancy‡**	
			
			**M**	**T**
Alpha-glucosidase I* (glcase 1)	3.2.1.106	Single glucose residues remotion from oligossaccharides	-	1
Phosphoglucomutase (pgm)	5.4.2.8	Synthesis of glucose	-	1
UDP-glucose pyrophosphorylase (ugp1)	2.7.7.9	Synthesis of UDP-Glucose	-	2
Alpha-1,3 glucan synthase (ags1)	2.4.1.183	Synthesis of α1–3-glucan	-	1
Mannitol-1-phosphate dehydrogenase (mtld)	1.1.1.17	Synthesis of fructose 6-phosphate	2	3
Monosaccharide transport protein (mstE)	-	Low affinity glucose uptake	-	1
Sugar transporter protein (stl1)	-	Uptake of hexoses	3	5
Chitinase 1(cts1)	3.2.1.14	Hydrolysis of chitin	1	2
Chitinase 3* (cts3)	3.2.1.14	Hydrolysis of chitin	-	1
Acidic amino acid permease (dip5)	-	Acidic amino acid uptake	9	9
Histidinol phosphate aminotransferase (hpat)	2.6.1.9	Synthesis of L-histidinol phosphate/glutamate	-	1
Malate permease (mael)	-	Uptake of Malate	-	2
UDP-N-acetylglucosamine transporter* (mnn2)	-	Required for transport of the chitin precursor to Golgi and Endoplasmic reticulum	-	1
Glucanosyltransferase family protein (gel)	2.4.1.-	Transglucosidase activity	1	3
Rho GTPAse activating protein* (bem3)	-	Regulation of the beta(1,3)-glucan synthase	-	1
Mannosyltransferase (mnt1)	2.4.1.131	Mannosylation of proteins/lipids	-	1
Alpha-1,2-mannosyltransferase (mnn5)	2.4.1.131	Mannosylation of proteins/lipids	3	3
Guanosine diphosphatase* (gdA1)	3.6.1.42	Synthesis of GMP	-	1
Alpha-1,2 galactosyltransferase* (gma12)	2.4.1.-	Galactose incorporation in N- and O-linked mannoproteins	-	1
Lysophospholipase (lpb1b)	3.1.1.5	Hydrolysis of phospholipids	-	1
Phospholipase A2 (plaA)	3.1.1.4	Hydrolysis of phospholipids	-	1
Glycerol-3-phosphate dehydrogenase* (NADP) (gfdA)	1.1.1.94	Synthesis of Glycerol-3-phosphate.	-	1
Glycerophosphodiester phosphodiesterase (gpdp)	3.1.4.46	Synthesis of choline and ethanolamine	1	4
Acyl-coenzyme A synthetase (acs)	6.2.1.3	Convertion of the fatty acid to acyl-coA for subsequent beta oxidation	-	1
Phosphatidylserine synthase* (pssA)	2.7.8.8	Glycerophospholipid metabolism/Phosphatidylserine synthesis	-	1
Myo-inositol-1-phosphate synthase (ino1)	5.5.1.4	Synthesis of myo-inositol 1 phosphate	-	1
Phosphatidylinositol transfer protein (pdr16)	-	Transport of phospholipids from their site of synthesis to cell membranes/Regulator of phospholipid biosynthesis	-	1
Lanosterol 14-alpha-demethylase (erg11)	1.14.13.70	Synthesis of ergosterol	3	4
Sterol delta 5,6-desaturase (erg3)	1.3.3.-	Regulation of ergosterol biosynthesis	-	1
Serine esterase (net1)	-	Catalysis of the cleavage of fatty acids from membrane lipids	-	3
Peroxisomal hydratase dehydrogenase epimerase (hde)	4.2.1.-	Beta oxidation	-	4
Fatty acid desaturase (desA)	1.14.99.-	Insaturation of acyl group of lipids	1	2
Carnitine dehydratase (caiB)	4.2.1.89	Transport of long-chain fatty acids	-	1
Suppressor of anucleate metulae B protein* (samB)	-	Morphogenesis regulation	-	1

‡ The predicted redundancy was obtained from the transition cDNA library in comparison to mycelia transcriptome database [1].

* Novel genes detected in *P. brasiliensis*.

**Table 2:** List of novel genes detected in the *P. brasiliensis *transition library.

**Functional categories**	**Gene Product**	**Best hit/Accession number**	**e-value**	**E.C. number**
**Amino acid metabolism**	Diphthine synthase^#^	*Aspergillus fumigatus*/CAF32112	1e-38	2.1.1.98
	Acetylornithine deacetylase	*Arabidopsis thaliana*/BP845946.1	1e-31	3.5.1.16
	Histidine ammonia-lyase	*Dictyostelium discoideum*/XP_636944.1	1e-16	4.3.1.3
	Glutamate dehydrogenase (NADP(+))	*Emericella nidulans*/S04904	5e-06	1.4.1.4
**Nucleotide metabolism**	Nudix hydrolase family protein	*Aspergillus nidulans*/XP_409279.1	1e-19	-
	Adenosine deaminase	*Aspergillus oryzae*/BAE60718	2e-34	3.5.4.4
	Orotate phosphoribosyltransferase	*Mortierella alpina*/BAD29963.1	3e-45	2.4.2.10
**Phosphate metabolism**	phnO protein	*Rhizopus oryzae*/EE002192.1	4e-116	-
**C-compound and carbohydrate metabolism**	Chitinase 3^#^	*Coccidioides immitis*/AAO88269	7e-40	3.2.1.14
	Alpha-glucosidase I^#^	*Aspergillus fumigatus*/AAR23808	3e-46	3.2.1.106
	Glycerol-3-phosphate dehydrogenase (NAD(P)+)	*Cryptococcus neoformans*/AAM26266.1	2e-14	1.1.1.94
**Lipid metabolism**	Phosphatidylserine synthase^#^	*Neurospora crassa*/EAA30566.1	6e-38	2.7.8.8
**Metabolism of vitamins, cofactors and prosthetic groups**	Uroporphyrinogen III methylase	*Rhizopus oryzae*/EE010378.1	6e-109	2.1.1.107
**Energy**	Xanthine dehydrogenase	*Gibberella zeae*/XP_381737.1	9e-07	1.17.1.4
	Acetyl CoA hydrolase	*Aspergillus nidulans*/XP_405684.1	5e-42	3.1.2.1
**Cell cycle and DNA processing**	Rad21 region protein	*Neurospora crassa*/EAA34981.1	6e-17	-
	Proliferating Cell Nuclear Antigen (PCNA)	*Aspergillus nidulans*/XP_404552.1	3e-36	-
	Uracil-DNA glycosylase	*Aspergillus fumigatus*/XP_749743	3e-24	3.2.2.-
	Chromosome segregation ATPase	*Coccidioides immitis*/EAS30662	6e-52	-
**Transcription**	DEAD-like helicases superfamily protein^#^	*Aspergillus nidulans*/XP_410144.1	3e-55	-
	Transcription factor, bromodomain	*Aspergillus nidulans*/EAA60972	2e-55	-
	GatB/YqeY domain protein	*Aspergillus nidulans*/XP_410874.1	1e-22	-
	Ring type Zinc finger protein	*Aspergillus nidulans*/XP_411042.1	1e-12	-
	Zinc finger domain protein	*Aspergillus nidulans*/XP_405585.1	3e-14	-
	Arylsulfatase regulatory protein	*Blastocladiella emersonii*/CO964913.1	1e-138	-
	Transcriptional activator protein	*Coccidioides immitis*/EAS34609	8e-26	-
**Protein Synthesis**	14 kDa mitochondrial ribosomal protein	*Aspergillus nidulans*/XP_408748.1	4e-46	-
	Translation initiation factor 3 subunit 2	*Aspergillus nidulans*/XP_660601	6e-80	-
**Protein fate**	Rab geranylgeranyl transferase	*Aspergillus nidulans*/XP_412816.1	8e-13	2.5.1.60
	Guanosine diphosphatase^#^	*Aspergillus nidulans*/XP_405219.1	2e-15	3.6.1.42
	Ubiquitin thiolesterase otubain-like protein	*Aspergillus nidulans/*EAA60354	1e-28	3.4.-.-
	Non-ATPase regulatory subunit of the 26S proteasome	*Aspergillus nidulans*/XP_408912.1	2e-68	-
	Peptidase M28 domain protein	*Coccidioides immitis*/EAS33583	1e-22	3.4.11.15
	Alpha -1, 2-galactosyltransferase^#^	*Aspergillus nidulans*/XP_406106.1	3e-14	2.4.1.-
**Transport Facilitation**	Uridine diphosphate N-Acetylglucosamine transporter^#^	*Neurospora crassa*/T50997	9e-30	-
	Nuclear pore protein 84/107	*Coccidioides immitis*/EAS31445.1|	2e-13	-
	Regulator of V-ATPase in vacuolar membrane protein	*Aspergillus nidulans*/XP_404840.1	9e-59	-
	Tctex-1 family protein	*Aspergillus nidulans*/XP_405470.1	6e-25	-
	Importin-beta N-terminal domain	*Aspergillus nidulans*/XP_410143.1	1e-44	-
**Signal Transduction**	Two-component sensor kinase	*Anopheles gambiae*/EAA02130.2	2e-38	-
	Histidine protein kinase sensor for GlnG regulator^#^	*Tetrahymena thermophila*/EAR83219.1	2e-04	2.7.3.13-
	UVSB Phosphatidylinositol – 3 kinase^#^	*Aspergillus nidulans*/XP_411112.1	1e-29	-
	Rho GTPase activating protein	*Aspergillus nidulans*/XP_407883.1	3e-49	-
	Calcineurin subunit b	*Neurospora crassa*/P87072	1e-77	-
	Forkhead associated (FHA) protein	*Gibberella zeae*/XP_389397.1	4e-10	-
**Cell Rescue, Defense and Virulence**	Hemolysin like protein^#^	*Aspergillus nidulans*/XP_406013.1	2e-70	-
**Cell type differentiation**	Suppressor of anucleate metulae B protein^#^	*Aspergillus nidulans*/XP_404215.1	6e-46	-
**Unclassified**	Complex 1 protein (LYR family)	*Aspergillus nidulans*/XP_408902.1	8e-32	-

^#^Transcripts confirmed by semi-quantitative RT-PCR.

**Table 3:** Candidate homologs for virulence factors induced in the cDNA transition library.

**Virulence determinant**	**Function in other fungi**	**Reference number**
Alpha -1,3 glucan synthase (ags1)	Reduction of AGS1 activity reduces the lung colonization by *Histoplasma capuslatum*	[40]
Glucanosyltransferase family protein (gel)	Required for both morphogenesis and virulence in *Aspergillus fumigatus*	[41]
Calcineurin subunit B (canB)	Required for *Candida albicans *virulence and stress resistance	[42]
Para-aminobenzoic acid synthetase (paba)	Essential for *Aspergillus fumigatus *growth in lung tissue	[43]
Peroxisomal catalase (cat P)	Putatively related to the *P. brasiliensis *protection against peroxides	[44]
Aspartyl protease (asp)	Facilitation of pathogenesis in *Candida albicans*	[45]
Zinc metalloprotease (mp)	A elastolytic metalloprotease of *Aspergillus fumigatus *is secreted during fungal invasion of murine lung	[46]
Phosholipase A2 (plaA)	Gene inactivation attenuates virulence in *Candida albicans*	[47]
Glyceraldehyde 3 phosphate dehydrogenase (gapdh)	Recombinant GAPDH and antibodies to GAPDH diminish *P. brasiliensis *yeast binding to and infection of A549 pneumocytes	[49]
Alpha-1,2 mannosyltransferase (mnn5)	Important for virulence of *Candida albicans*	[50]
Hemolysin like protein (hlp)	Phase specific gene regulated by phenotypic switching in *Candida glabrata*	[51]
Urease (ure)	Required for *Coccidioides posadasii *virulence	[52]

**Table 4:** Oligonucleotides primers related to new genes selected for sqRT-PCR analysis.

**Sequence name**	**Forward primer (5' → 3')**	**Reverse primer (5' → 3')**
DEAD-like helicases superfamily protein (dead)	GGCCTTCTGAAACGGGGG	GAGCTTCGCAATAGGCCAAG
Hemolysin like protein (hlp)	GGCCTTCTGAAACGGGGG	GAGCTTCGCAATAGGCCAAG
UVSB Phosphatidylinosytol-3-kinase (uvsB)	CTAGCGAATGGCAATATCACT	GATAATGAGGGCATGGTCTC
Chitinase 3 (cts3)	GGAGGAGGATATGTCTCTTG	CTGCTGCCCATCCCTCAG
Alpha 1,2 galactosyltransferase (gma12)	GCTATGTCAACTTCTTCGCG	GAGAGCATGGGCCGACAG
UDP-N-Acetylglucosamine transporter (mnn2)	GCCCTCATTACGTTAACGCA	CATGGATTTTCCTTTGGCACT
Guanosine diphosphatase (gdpase)	GATCTTCCGCTTTCTCGCCA	CTCCTTGACACGGCACTGC
Suppressor of anucleate metulae B protein (samB)	CCAGTGCGCCTACTATAAATG	CAGGCATTCTTCTGGCACTC
Diphitine synthase (dphs)	CTGTTTCGCAGTGTGCCAG	CGTTCCGTAATTGCTTTTCCA
Phosphatidylserine synthase (pss)	GCTGCTCTCGGCGGACTC	CGAAGGAGACCAGATCAGC
Alpha glucosidase I (glcaseI)	CCAGCTGATAGTCCACGGC	CTTGTCCATCCTGTGAAATGC
Histidine protein kinase sensor for GlnG regulator (glnL)	CGTCTGTTGGGGCCGCAG	CATCGGGTAAAACAGCGTATC
