# Supplementary material for: The transcriptome analysis of early morphogenesis in Paracoccidioides brasiliensis mycelium reveals novel and induced genes potentially associated to the dimorphic process
Source: BMC Microbiol. 2007 Apr 10;7:29. doi: 10.1186/1471-2180-7-29 (PMC1855332; doi:10.1186/1471-2180-7-29)
Supplement: Additional File 1 — P. brasiliensis clusters annotated in the cDNA library. Table representing the annotated clusters that were generated by sequencing of the cDNA clones. For each cluster the table includes: the function as assigned by BLAST-based similarity, the BLAST subject species, the GenBank ID for the BLAST subject used for functional assignment and the Expect value obtained with each unisequence, the redundancy in the transition library and in the mycelium transcriptome database. [file 1471-2180-7-29-S1.doc]

**Table 1 – *P. brasiliensis* clusters annotated in the cDNA library**.

| **MIPS Category** | **Gene Product** | **Best hit/Accession number** | **e-value** | **Redundancy** | |
| --- | --- | --- | --- | --- | --- |
| **M** | **T** |
| **Metabolism** | D-amino acid oxidase | [*Aspergillus nidulans*/XP_404311](http://200.137.194.69/phorestwww/ncbi.php?accno=40746891) | [2e -47](http://200.137.194.69/phorestwww/xmlparse.php?clone=10215&frame=1&accno=40746891#x) | 5 | 2 |
|  | Histidinol phosphate aminotransferase^b^ | [*Aspergillus fumigatus*](http://200.137.194.69/phorestwww/ncbi.php?accno=44890004)/CAF32122 | [1e -69](http://200.137.194.69/phorestwww/xmlparse.php?clone=10803&frame=2&accno=44890004#x) | - | 1 |
|  | Diphthine synthase*^#^ | [*Aspergillus fumigatus*](http://200.137.194.69/phorestwww/ncbi.php?accno=44889994)/CAF32112 | [1e -38](http://200.137.194.69/phorestwww/xmlparse.php?clone=10808&frame=2&accno=44889994#x) | - | 2 |
|  | Acetylornithine deacetylase* | *Arabidopsis thaliana*/BP845946 | 1e -31 | - | 1 |
|  | Glutamic acid decarboxylase 1 | [*Aspergillus nidulans*/XP_409022](http://200.137.194.69/phorestwww/ncbi.php?accno=40741773) | [4e -36](http://200.137.194.69/phorestwww/xmlparse.php?clone=9952&frame=2&accno=40741773#x) | 1 | 1 |
|  | Gamma-glutamyl phosphate reductase^+^ | *Coccidioides immitis*/ EAS33218 | 1e -21 | - | 1 |
|  | Methionine adenosyltransferase | *Aspergillus oryzae*/ BAE64158 | 8e -13 | 2 | 1 |
|  | Acetolactate synthase, regulatory subunit | *Coccidioides immitis*/ EAS33057 | 2e -64 | 4 | 1 |
|  | Anthranilate synthase | *Aspergillus terreus*/XP_001210590 | 5e -22 | 1 | 1 |
|  | Kynurenine aminotransferase | *Magnaporthe grisea* **/**XP_360721 | 1e -38 | 1 | 1 |
|  | Homogentisate 1,2-dioxygenase | *Coccidioides immitis*/EAS35958 | 1e -58 | 13 | 3 |
|  | Fumarylacetoacetate hydrolase^b^ | *Emericella nidulans*/AAA85778 | [1e -69](http://200.137.194.69/phorestwww/xmlparse.php?clone=10452&frame=1&accno=1130507#x) | - | 2 |
|  | 2,4-dihydroxyhept-2-ene-1,7-dioic acid aldolase^b^ | [*Aspergillus nidulans* /XP_407838](http://200.137.194.69/phorestwww/ncbi.php?accno=40740719) | [5e -07](http://200.137.194.69/phorestwww/xmlparse.php?clone=10562&frame=3&accno=40740719#x) | - | 1 |
|  | Anthranilate phosphoribosyltransferase^a^ | [*Aspergillus nidulans* /XP_407771](http://200.137.194.69/phorestwww/ncbi.php?accno=40740652) | [2e -44](http://200.137.194.69/phorestwww/xmlparse.php?clone=10273&frame=-2&accno=40740652#x) | 1 | 2 |
|  | Histidine ammonia lyase* | *Dictyostelium discoideum*/XP_636944 | 1e -16 | - | 1 |
|  | **Glutamate dehydrogenase (NADP(+))*** | *Emericella nidulans*/ S04904 | 5e -06 | - | 2 |
|  | Ketol-acid reductoisomerase | [*Aspergillus nidulans*/XP_406663](http://200.137.194.69/phorestwww/ncbi.php?accno=40745475) | [3e -68](http://200.137.194.69/phorestwww/xmlparse.php?clone=10904&frame=1&accno=40745475#x) | 1 | 1 |
|  | 3-Methylcrotonyl-CoA carboxylase non-biotin-containing subunit | [*Emericella nidulans*](http://200.137.194.69/phorestwww/ncbi.php?accno=38505140)/AAR23111 | [2e -21](http://200.137.194.69/phorestwww/xmlparse.php?clone=9965&frame=3&accno=38505140#x) | 3 | 1 |
|  | Nitrogen regulatory protein P-II^b^ | [*Aspergillus nidulans*/XP_408436](http://200.137.194.69/phorestwww/ncbi.php?accno=40740849) | [5e -37](http://200.137.194.69/phorestwww/xmlparse.php?clone=10006&frame=2&accno=40740849#x) | - | 2 |
|  | Acetamidase^b^ | [*Aspergillus nidulans*/XP_405649](http://200.137.194.69/phorestwww/ncbi.php?accno=40744669) | [2e -53](http://200.137.194.69/phorestwww/xmlparse.php?clone=9974&frame=2&accno=40744669#x) | - | 1 |
|  | Sulfite reductase beta subunit^b^ | [*Aspergillus nidulans*/XP_411737](http://200.137.194.69/phorestwww/ncbi.php?accno=40742990) | [3e -75](http://200.137.194.69/phorestwww/xmlparse.php?clone=10045&frame=2&accno=40742990#x) | - | 1 |
|  | Urease, alpha subunit^a^ | [*Aspergillus fumigatus*](http://200.137.194.69/phorestwww/ncbi.php?accno=50788080)/CAE17672 | [1e -71](http://200.137.194.69/phorestwww/xmlparse.php?clone=10685&frame=3&accno=50788080#x) | 1 | 4 |
|  | Cyanate lyase^b^ | [*Aspergillus nidulans*/XP_411468](http://200.137.194.69/phorestwww/ncbi.php?accno=40742192) | [6e -46](http://200.137.194.69/phorestwww/xmlparse.php?clone=10622&frame=3&accno=40742192#x) | - | 1 |
|  | Thiosulfate sulphurtransferase^a^ | [*Gibberella zeae*/XP_381684](http://200.137.194.69/phorestwww/ncbi.php?accno=42546050) | [1e -45](http://200.137.194.69/phorestwww/xmlparse.php?clone=10605&frame=2&accno=42546050#x) | 1 | 2 |
|  | Phosphoribosylpyrophosphate amidotransferase | *Coccidioides immitis*/ EAS27535 | 2e -54 | 6 | 1 |
|  | Nudix hydrolase family protein* | [*Aspergillus nidulans*/XP_409279](http://200.137.194.69/phorestwww/ncbi.php?accno=40743133) | [1e -19](http://200.137.194.69/phorestwww/xmlparse.php?clone=10834&frame=2&accno=40743133#x) | - | 3 |
|  | Quinolinate phosphoribosyl transferase | [*Aspergillus oryzae*](http://200.137.194.69/phorestwww/ncbi.php?accno=40809689)/BAD07264 | [2e -48](http://200.137.194.69/phorestwww/xmlparse.php?clone=10525&frame=3&accno=40809689#x) | 6 | 1 |
|  | GMP synthase | *Phaeosphaeria nodorum*/[EAT84008](http://www.ncbi.nlm.nih.gov/entrez/query.fcgi?cmd=Retrieve&db=Protein&list_uids=111062888&dopt=GenPept) | 9e -61 | 1 | 1 |
|  | Ribose phosphate diphosphokinase | [*Neurospora crassa*/EAA32555](http://200.137.194.69/phorestwww/ncbi.php?accno=32408259) | [3e -44](http://200.137.194.69/phorestwww/xmlparse.php?clone=10091&frame=1&accno=32408259#x) | 1 | 1 |
|  | RNA (guanine-N7) methyltransferase^a^ | [*Neurospora crassa*](http://200.137.194.69/phorestwww/ncbi.php?accno=40882314)/CAF06136 | [2e -18](http://200.137.194.69/phorestwww/xmlparse.php?clone=10483&frame=3&accno=40882314#x) | 1 | 2 |
|  | Adenine phosphoribosyltransferase 1 | [*Aspergillus nidulan*s/XP_413220](http://200.137.194.69/phorestwww/ncbi.php?accno=40742726) | [2e -26](http://200.137.194.69/phorestwww/xmlparse.php?clone=10401&frame=3&accno=40742726#x) | 2 | 2 |
|  | Adenosine deaminase * | *Aspergillus oryzae***/**BAE60718 | 2e -34 | - | 4 |
|  | Orotate phosphoribosyltransferase* | [*Mortierella alpina*](http://www.ncbi.nlm.nih.gov/Taxonomy/Browser/wwwtax.cgi?id=64518)/BAD29963 | 3e -45 | - | 2 |
|  | Nucleoside diphosphate kinase | [*Gibberella zeae*/XP_386148](http://200.137.194.69/phorestwww/ncbi.php?accno=42552774) | [3e -69](http://200.137.194.69/phorestwww/xmlparse.php?clone=10237&frame=3&accno=42552774#x) | 7 | 2 |
|  | Uracil phosphoribosyltransferase | [*Neurospora crassa*/EAA33629](http://200.137.194.69/phorestwww/ncbi.php?accno=32411271) | [1e -30](http://200.137.194.69/phorestwww/xmlparse.php?clone=10242&frame=2&accno=32411271#x) | 2 | 1 |
|  | Pyrimidine 5-nucleotidase^+^ | [*Aspergillus nidulans*/XP_410462](http://200.137.194.69/phorestwww/ncbi.php?accno=40739519) | [4e -49](http://200.137.194.69/phorestwww/xmlparse.php?clone=10435&frame=3&accno=40739519#x) | - | 2 |
|  | TatD DNAse^b^ | [*Aspergillus nidulans*/XP_408185](http://200.137.194.69/phorestwww/ncbi.php?accno=40740329) | [4e -10](http://200.137.194.69/phorestwww/xmlparse.php?clone=10794&frame=1&accno=40740329#x) | - | 1 |
|  | YjgF-like protein | [*Aspergillus nidulans*/XP_413217](http://200.137.194.69/phorestwww/ncbi.php?accno=40742723) | [1e -11](http://200.137.194.69/phorestwww/xmlparse.php?clone=10532&frame=1&accno=40742723#x) | 2 | 1 |
|  | Arylesterase region protein | *Gibberella zeae* /XP_388094 | [2e -12](http://200.137.194.69/phorestwww/xmlparse.php?clone=10351&frame=2&accno=42554447#x) | 17 | 1 |
|  | phnO protein* | *Rhizopus oryzae*/EE002192 | 4e -116 | - | 4 |
|  | Inorganic pyrophosphatase^b^ | *Coccidioides immitis*/ EAS28880 | 3e -32 | - | 1 |
|  | Alpha-1,3-glucan synthase^b^ | [*Paracoccidioides brasiliensis*/](http://200.137.194.69/phorestwww/ncbi.php?accno=55509190)AAV52833 | [8e -68](http://200.137.194.69/phorestwww/xmlparse.php?clone=9964&frame=1&accno=55509190#x) | - | 1 |
|  | Glucanosyltransferase family protein^a^ | *Aspergillus nidulans*/XP_408051 | [9e -35](http://200.137.194.69/phorestwww/xmlparse.php?clone=9708&frame=2&accno=40740033#x) | 1 | 3 |
|  | Chitinase 1^a^ | [*Aspergillus nidulans*/XP_413527](http://200.137.194.69/phorestwww/ncbi.php?accno=40747301) | [1e -1](http://200.137.194.69/phorestwww/xmlparse.php?clone=10380&frame=1&accno=40747301#x)8 | 1 | 2 |
|  | Chitinase 3*^#^ | [*Coccidioides immitis*/](http://200.137.194.69/phorestwww/ncbi.php?accno=29539602)AAO88269 | [7e -40](http://200.137.194.69/phorestwww/xmlparse.php?clone=10213&frame=3&accno=29539602#x) | - | 1 |
|  | Beta-1,3-endoglucanase | [*Aspergillus nidulans*/XP_404609](http://200.137.194.69/phorestwww/ncbi.php?accno=40747415) | [1e -63](http://200.137.194.69/phorestwww/xmlparse.php?clone=10093&frame=2&accno=40747415#x) | 4 | 2 |
|  | Glycosyl hydrolase family protein | [*Aspergillus nidulans*/XP_408041](http://200.137.194.69/phorestwww/ncbi.php?accno=40739645) | [8e -15](http://200.137.194.69/phorestwww/xmlparse.php?clone=10275&frame=1&accno=40739645#x) | 1 | 1 |
|  | Glucan 1,3 beta-glucosidase-like protein | [*Aspergillus nidulans*/XP_408837](http://200.137.194.69/phorestwww/ncbi.php?accno=40741552) | [1e -53](http://200.137.194.69/phorestwww/xmlparse.php?clone=10345&frame=2&accno=40741552#x) | 7 | 2 |
|  | Cell wall organization and biogenesis related protein | [*Aspergillus nidulans*/XP_405599](http://200.137.194.69/phorestwww/ncbi.php?accno=40745436) | [2e -18](http://200.137.194.69/phorestwww/xmlparse.php?clone=10341&frame=1&accno=40745436#x) | 1 | 1 |
|  | Hydroxyproline-rich glycoprotein | *Zea diploperennis*/CAA45514 | [4e -07](http://200.137.194.69/phorestwww/xmlparse.php?clone=6947&frame=2&accno=22092#x) | 16 | 1 |
|  | Phosphatidylinositol N-acetylglucosaminyltransferase subunit P | [*Aspergillus nidulans*/XP_412207](http://200.137.194.69/phorestwww/ncbi.php?accno=40740502) | [1e -29](http://200.137.194.69/phorestwww/xmlparse.php?clone=10776&frame=3&accno=40740502#x) | 3 | 1 |
|  | 6-phosphogluconolactonase 1 | [*Aspergillus nidulans*/XP_404422](http://200.137.194.69/phorestwww/ncbi.php?accno=40747002) | [1e -43](http://200.137.194.69/phorestwww/xmlparse.php?clone=10238&frame=1&accno=40747002#x) | 7 | 4 |
|  | Transaldolase | [*Gibberella zeae*/XP_388899](http://200.137.194.69/phorestwww/ncbi.php?accno=42549577) | [9e -25](http://200.137.194.69/phorestwww/xmlparse.php?clone=10397&frame=3&accno=42549577#x) | 4 | 2 |
|  | Malate dehydrogenase | [*Paracoccidioides brasiliensis*/](http://200.137.194.69/phorestwww/ncbi.php?accno=47119068)AAP37966 | [6e -81](http://200.137.194.69/phorestwww/xmlparse.php?clone=10708&frame=3&accno=47119068#x) | 1 | 1 |
|  | Glucose/ribitol dehydrogenase | [*Aspergillus nidulans*/XP_409694](http://200.137.194.69/phorestwww/ncbi.php?accno=40743072) | [1e -08](http://200.137.194.69/phorestwww/xmlparse.php?clone=10731&frame=2&accno=40743072#x) | 3 | 1 |
|  | Polysaccharide deacetylase family protein | [*Aspergillus nidulan/s*XP_410655](http://200.137.194.69/phorestwww/ncbi.php?accno=40738668) | [1e -96](http://200.137.194.69/phorestwww/xmlparse.php?clone=16478&frame=3&accno=40738668#x) | 4 | 3 |
|  | Phosphopyruvate hydratase | *Aspergillus oryzae*/Q12560 | [2e -74](http://200.137.194.69/phorestwww/xmlparse.php?clone=10674&frame=2&accno=3023683#x) | 2 | 1 |
|  | Uridine diphosphate glucose pyrophosphorylase^a^ | [*Emericella nidulans*/](http://200.137.194.69/phorestwww/ncbi.php?accno=57236787)AAW49005 | [3e -69](http://200.137.194.69/phorestwww/xmlparse.php?clone=10002&frame=3&accno=57236787#x) | 1 | 2 |
|  | Alpha-glucosidase I*^#^ | [*Aspergillus fumigatus*](http://200.137.194.69/phorestwww/ncbi.php?accno=38564743)/AAR23808 | [3e -46](http://200.137.194.69/phorestwww/xmlparse.php?clone=10632&frame=3&accno=38564743#x) | - | 1 |
|  | Mannitol-1-phosphate dehydrogenase^a^ | [*Paracoccidioides brasiliensis*/](http://200.137.194.69/phorestwww/ncbi.php?accno=28797565)AAO47089 | [2e -88](http://200.137.194.69/phorestwww/xmlparse.php?clone=10439&frame=3&accno=28797565#x) | 2 | 3 |
|  | Myo-inositol-1-phosphate synthase^+^ | [*Aspergillus nidulans*/XP_411762](http://200.137.194.69/phorestwww/ncbi.php?accno=40742621) | [4e -15](http://200.137.194.69/phorestwww/xmlparse.php?clone=10344&frame=1&accno=40742621#x) | - | 1 |
|  | Glycerophosphodiester phosphodiesterase^a^ | [*Aspergillus nidulans*/XP_404274](http://200.137.194.69/phorestwww/ncbi.php?accno=40746159) | [9e -70](http://200.137.194.69/phorestwww/xmlparse.php?clone=10540&frame=3&accno=40746159#x) | 1 | 4 |
|  | Diacylglycerol pyrophosphate phosphatase | [*Aspergillus nidulans*/XP_406261](http://200.137.194.69/phorestwww/ncbi.php?accno=40745800) | [2e -56](http://200.137.194.69/phorestwww/xmlparse.php?clone=10355&frame=2&accno=40745800#x) | 1 | 1 |
|  | Esterase family protein^+^ | [*Aspergillus nidulans* /XP_412451](http://200.137.194.69/phorestwww/ncbi.php?accno=40747781) | [2e -13](http://200.137.194.69/phorestwww/xmlparse.php?clone=10814&frame=3&accno=40747781#x) | - | 1 |
|  | Lysophospholipase^b^ | [*Aspergillus nidulans*/XP_412885](http://200.137.194.69/phorestwww/ncbi.php?accno=40741351) | [3e -58](http://200.137.194.69/phorestwww/xmlparse.php?clone=10606&frame=2&accno=40741351#x) | - | 1 |
|  | Phospholipase A2^+^ | *Coccidioides immitis*/ EAS34384 | 3e -27 | - | 1 |
|  | 3-ketoacyl-CoA thiolase | *Aspergillus fumigatus*/XP_755468 | 9e -50 | 2 | 1 |
|  | Peroxisomal hydratase dehydrogenase epimerase^b^ | [*Aspergillus nidulans*/XP_411248](http://200.137.194.69/phorestwww/ncbi.php?accno=40742126) | [1e -34](http://200.137.194.69/phorestwww/xmlparse.php?clone=10760&frame=2&accno=40742126#x) | - | 4 |
|  | Delta(24) sterol C-methyltransferase | [*Gibberella zeae*/XP_382959](http://200.137.194.69/phorestwww/ncbi.php?accno=42547935) | [1e -74](http://200.137.194.69/phorestwww/xmlparse.php?clone=10597&frame=3&accno=42547935#x) | 2 | 1 |
|  | Serine esterase^+^ | [*Aspergillus nidulans*/XP_406618](http://200.137.194.69/phorestwww/ncbi.php?accno=40744643) | [8e -92](http://200.137.194.69/phorestwww/xmlparse.php?clone=10364&frame=1&accno=40744643#x) | - | 3 |
|  | Glycerol-3-phosphate dehydrogenase (NAD(P)+)* | *Cryptococcus neoformans*/AAM26266 | 2e -14 | - | 1 |
|  | NADH ubiquinone oxidoreductase | *Aspergillus fumigatus* /XP_755823 | 2e -46 | 6 | 1 |
|  | Phosphatidylserine synthase*^#^ | [*Neurospora crassa*/EAA30566](http://200.137.194.69/phorestwww/ncbi.php?accno=32421427) | [6e -38](http://200.137.194.69/phorestwww/xmlparse.php?clone=10504&frame=3&accno=32421427#x) | - | 1 |
|  | 3-oxoacyl (acyl-carrier-protein) reductase | [*Aspergillus nidulans*/XP_410792](http://200.137.194.69/phorestwww/ncbi.php?accno=40738994) | [4e -41](http://200.137.194.69/phorestwww/xmlparse.php?clone=9984&frame=1&accno=40738994#x) | 9 | 3 |
|  | Trans-2-enoyl-CoA reductase | [*Aspergillus nidulans*/XP_413538](http://200.137.194.69/phorestwww/ncbi.php?accno=40747312) | [7e -42](http://200.137.194.69/phorestwww/xmlparse.php?clone=10915&frame=1&accno=40747312#x) | 4 | 1 |
|  | Fatty acid desaturase^a^ | [*Neurospora crassa*/EAA29146](http://200.137.194.69/phorestwww/ncbi.php?accno=32420673) | [4e -48](http://200.137.194.69/phorestwww/xmlparse.php?clone=10873&frame=3&accno=32420673#x) | 1 | 2 |
|  | Oxysterol binding protein homolog 7 | [*Magnaporthe grisea*/XP_365104](http://200.137.194.69/phorestwww/ncbi.php?accno=38099372) | [1e -76](http://200.137.194.69/phorestwww/xmlparse.php?clone=10509&frame=1&accno=38099372#x) | 1 | 1 |
|  | Sterol delta 5,6-desaturase ERG3^b^ | [*Aspergillus nidulans*/XP_410643](http://200.137.194.69/phorestwww/ncbi.php?accno=40738656) | [7e -40](http://200.137.194.69/phorestwww/xmlparse.php?clone=10055&frame=2&accno=40738656#x) | - | 1 |
|  | Lanosterol 14-alpha-demethylase^a^ | [*Ajellomyces capsulatus*](http://200.137.194.69/phorestwww/ncbi.php?accno=51341096)/AAU01158 | [1e -89](http://200.137.194.69/phorestwww/xmlparse.php?clone=9956&frame=3&accno=51341096#x) | 3 | 4 |
|  | Lipoic acid synthase^b^ | [*Aspergillus nidulans*/XP_413623](http://200.137.194.69/phorestwww/ncbi.php?accno=40747621) | [8e -40](http://200.137.194.69/phorestwww/xmlparse.php?clone=10131&frame=3&accno=40747621#x) | - | 1 |
|  | Uroporphyrinogen III methylase* | [*Rhizopus oryzae*](http://www.ncbi.nlm.nih.gov/Taxonomy/Browser/wwwtax.cgi?name=Rhizopus+oryzae)/EE010378 | 6e -109 | - | 4 |
|  | Ubiquinone/menaquinone biosynthesis methlytransferase UbiE^b^ | *Dictyostelium discoideum*/XP_641323 | 2e -12 | - | 18 |
|  | Para aminobenzoic acid synthetase^b^ | [*Aspergillus nidulans*/XP_410687](http://200.137.194.69/phorestwww/ncbi.php?accno=40738700) | [8e -19](http://200.137.194.69/phorestwww/xmlparse.php?clone=10879&frame=2&accno=40738700#x) | - | 1 |
|  | Acid phosphatase | [*Aspergillus nidulans*/XP_409104](http://200.137.194.69/phorestwww/ncbi.php?accno=40741855) | [6e -45](http://200.137.194.69/phorestwww/xmlparse.php?clone=17628&frame=1&accno=40741855#x) | 1 | 1 |
|  | Gephyrin | [*Aspergillus nidulans*/XP_407915](http://200.137.194.69/phorestwww/ncbi.php?accno=40740796) | [7e -49](http://200.137.194.69/phorestwww/xmlparse.php?clone=10062&frame=3&accno=40740796#x) | 1 | 1 |
|  | Pyridoxine biosynthesis protein pdx1 | [*Aspergillus nidulans*/XP_411862](http://200.137.194.69/phorestwww/ncbi.php?accno=40742050) | [7e -51](http://200.137.194.69/phorestwww/xmlparse.php?clone=10232&frame=2&accno=40742050#x) | 5 | 1 |
|  | Dephospho-CoA kinase | [*Neurospora crassa*/EAA28019](http://200.137.194.69/phorestwww/ncbi.php?accno=32403684) | [7e -13](http://200.137.194.69/phorestwww/xmlparse.php?clone=10019&frame=2&accno=32403684#x) | 2 | 1 |
|  | FabG region protein | [*Magnaporthe grisea*/XP_367544](http://200.137.194.69/phorestwww/ncbi.php?accno=38106938) | [2e -07](http://200.137.194.69/phorestwww/xmlparse.php?clone=10486&frame=2&accno=38106938#x) | 1 | 1 |
|  | Isocitrate lyase | [*Coccidioides immitis*/AAK72548](http://200.137.194.69/phorestwww/ncbi.php?accno=16304093) | [6e -65](http://200.137.194.69/phorestwww/xmlparse.php?clone=10337&frame=2&accno=16304093#x) | 5 | 2 |
|  | Isocitrate dehydrogenase (NADP+) | [*Aspergillus nidulans*/](http://200.137.194.69/phorestwww/ncbi.php?accno=15027826)AAK76730 | [2e -58](http://200.137.194.69/phorestwww/xmlparse.php?clone=10005&frame=2&accno=15027826#x) | 2 | 1 |
|  | Aconitase | [*Aspergillus terreus*/](http://200.137.194.69/phorestwww/ncbi.php?accno=3661614)AAC61778 | [2e -79](http://200.137.194.69/phorestwww/xmlparse.php?clone=10546&frame=3&accno=3661614#x) | 2 | 1 |
|  |  |  |  |  |  |
|  |  |  |  |  |  |
| **Energy** | Phosphoglucomutase^b^ | [*Neurospora crassa*/EAA34468](http://200.137.194.69/phorestwww/ncbi.php?accno=32405624) | [5e -73](http://200.137.194.69/phorestwww/xmlparse.php?clone=10074&frame=3&accno=32405624#x) | - | 1 |
|  | Enolase 1 | [*Penicillium chrysogenum*](http://200.137.194.69/phorestwww/ncbi.php?accno=34392443)/BAC82549 | [3e -47](http://200.137.194.69/phorestwww/xmlparse.php?clone=10575&frame=2&accno=34392443#x) | 4 | 3 |
|  | Triose phosphate isomerase | [*Paracoccidioides brasiliensis*/AAP02959](http://200.137.194.69/phorestwww/ncbi.php?accno=41614796) | [3e -59](http://200.137.194.69/phorestwww/xmlparse.php?clone=10287&frame=1&accno=41614796#x) | 3 | 1 |
|  | Fructose 1,6-biphosphate aldolase 1 | [*Paracoccidioides brasiliensis*/AAL25625](http://200.137.194.69/phorestwww/ncbi.php?accno=29826036) | [3e -65](http://200.137.194.69/phorestwww/xmlparse.php?clone=10111&frame=3&accno=29826036#x) | 5 | 2 |
|  | Phosphoenolpyruvate carboxylkinase | [*Aspergillus nidulans*/XP_406055](http://200.137.194.69/phorestwww/ncbi.php?accno=40745927) | [3e -53](http://200.137.194.69/phorestwww/xmlparse.php?clone=10813&frame=1&accno=40745927#x) | 2 | 1 |
|  | Glyceraldehyde-3-phosphate dehydrogenase^b^ | [*Paracoccidioides brasiliensis*](http://200.137.194.69/phorestwww/ncbi.php?accno=30995493)/AAL34975 | [7e -70](http://200.137.194.69/phorestwww/xmlparse.php?clone=14021&frame=2&accno=30995493#x) | - | 1 |
|  | NADH dehydrogenase, 21 kDa subunit^b^ | [*Aspergillus nidulans*/XP_411113](http://200.137.194.69/phorestwww/ncbi.php?accno=40742432) | [4e -38](http://200.137.194.69/phorestwww/xmlparse.php?clone=9999&frame=1&accno=40742432#x) | - | 1 |
|  | Xanthine dehydrogenase* | *Gibberella zeae*/XP_381737 | 9e -07 | - | 1 |
|  | NADP-cytochrome P450 reductase | [*Aspergillus nidulans*](http://200.137.194.69/phorestwww/ncbi.php?accno=40747538)/EAA66694 | [7e -67](http://200.137.194.69/phorestwww/xmlparse.php?clone=10566&frame=1&accno=40747538#x) | 3 | 2 |
|  | Ubiquinol cytochrome c reductase^b^ | [*Aspergillus nidulans*/XP_408525](http://200.137.194.69/phorestwww/ncbi.php?accno=40741115) | [6e -39](http://200.137.194.69/phorestwww/xmlparse.php?clone=9993&frame=3&accno=40741115#x) | - | 2 |
|  | Ubiquinol cytochrome c reductase hinge protein | [*Aspergillus nidulansg*/XP_409734](http://200.137.194.69/phorestwww/ncbi.php?accno=40743050) | [2e -19](http://200.137.194.69/phorestwww/xmlparse.php?clone=10048&frame=2&accno=40743050#x) | 2 | 1 |
|  | Cytochrome c oxidase subunit Va | [*Neurospora crassa*/CAD70919](http://200.137.194.69/phorestwww/ncbi.php?accno=32413002) | [1e -16](http://200.137.194.69/phorestwww/xmlparse.php?clone=9971&frame=2&accno=32413002#x) | 1 | 1 |
|  | NADH-ubiquinone oxidoreductase | [*Aspergillus nidulans*/XP_408819](http://200.137.194.69/phorestwww/ncbi.php?accno=40741534) | [2e -20](http://200.137.194.69/phorestwww/xmlparse.php?clone=10869&frame=3&accno=40741534#x) | 2 | 1 |
|  | NADH-ubiquinone oxidoreductase B18 subunit | [*Neurospora crassa*/EAA28195](http://200.137.194.69/phorestwww/ncbi.php?accno=32414025) | [7e -34](http://200.137.194.69/phorestwww/xmlparse.php?clone=10791&frame=2&accno=32414025#x) | 1 | 1 |
|  | NADH-ubiquinone oxidoreductase 49 kDa subunit, mitochondrial precursor | *Gibberella zeae*/EAA69636 | [5e -95](http://200.137.194.69/phorestwww/xmlparse.php?clone=18969&frame=2&accno=42546793#x) | 6 | 2 |
|  | Ferredoxin-like iron-sulfur protein ^a^ | [*Paracoccidioides brasiliensis*](http://200.137.194.69/phorestwww/ncbi.php?accno=30351132)/AAP23044 | [2e -58](http://200.137.194.69/phorestwww/xmlparse.php?clone=10307&frame=1&accno=30351132#x) | 2 | 3 |
|  | Protein PET191, mitochondrial precursor^b^ | [*Gibberella zeae*/XP_388901](http://200.137.194.69/phorestwww/ncbi.php?accno=42549579) | [4e -11](http://200.137.194.69/phorestwww/xmlparse.php?clone=10862&frame=1&accno=42549579#x) | - | 1 |
|  | Citrate synthase | [*Aspergillus nidulans*/](http://200.137.194.69/phorestwww/ncbi.php?accno=40739823)EAA59013 | [1e -62](http://200.137.194.69/phorestwww/xmlparse.php?clone=10642&frame=2&accno=40739823#x) | 2 | 1 |
|  | Mitochondrial ATP synthase epsilon chain | [*Magnaporthe grisea*/XP_360684](http://200.137.194.69/phorestwww/ncbi.php?accno=38105174) | [1e -19](http://200.137.194.69/phorestwww/xmlparse.php?clone=10752&frame=1&accno=38105174#x) | 5 | 3 |
|  | ATP synthase gamma chain^a^ | [*Aspergillus nidulans*/XP_404389](http://200.137.194.69/phorestwww/ncbi.php?accno=40746969) | [9e -57](http://200.137.194.69/phorestwww/xmlparse.php?clone=10505&frame=1&accno=40746969#x) | 1 | 3 |
|  | ATP synthase subunit 9^a^ | *Neurospora crassa*/T43671 | [8e -22](http://200.137.194.69/phorestwww/xmlparse.php?clone=10115&frame=3&accno=11266857#x) | 1 | 4 |
|  | Vacuolar ATP synthase subunit H | [Aspergillus nidulans/XP_405348](http://200.137.194.69/phorestwww/ncbi.php?accno=40746648) | [1e -34](http://200.137.194.69/phorestwww/xmlparse.php?clone=10753&frame=1&accno=40746648#x) | 2 | 1 |
|  | Acyl-coenzyme A synthetase^b^ | [*Neurospora crassa*/EAA26946](http://200.137.194.69/phorestwww/ncbi.php?accno=32403204) | [5e -10](http://200.137.194.69/phorestwww/xmlparse.php?clone=10580&frame=2&accno=32403204#x) | - | 1 |
|  | Phosphoglycerate mutase-like superfamily protein | [*Gibberella zeae*/XP_380582](http://200.137.194.69/phorestwww/ncbi.php?accno=42544654) | [2e -32](http://200.137.194.69/phorestwww/xmlparse.php?clone=10576&frame=1&accno=42544654#x) | 5 | 2 |
|  | L-carnitine dehydratase^+^ | [*Aspergillus nidulans*/XP_408367](http://200.137.194.69/phorestwww/ncbi.php?accno=40740139) | [1e -62](http://200.137.194.69/phorestwww/xmlparse.php?clone=10090&frame=3&accno=40740139#x) | - | 1 |
|  | Acetyl CoA hydrolase* | [*Aspergillus nidulans*/XP_405684](http://200.137.194.69/phorestwww/ncbi.php?accno=40745098) | [5e -42](http://200.137.194.69/phorestwww/xmlparse.php?clone=10067&frame=3&accno=40745098#x) | - | 1 |
|  | 2-methylcitrate dehydratase | [*Aspergillus nidulans*/XP_410776](http://200.137.194.69/phorestwww/ncbi.php?accno=40738978) | [4e -73](http://200.137.194.69/phorestwww/xmlparse.php?clone=10445&frame=2&accno=40738978#x) | 6 | 1 |
|  | Sorbitol dehydrogenase | *Paracoccidioides brasiliensis*/ AAL25624 | 2e -28 | 74 | 2 |
|  |  |  |  |  |  |
| **Cell cycle and DNA processing** | CHPA, a cysteine- and histidine-rich-domain-containing protein | [*Emericella nidulans*/AAR23267](http://200.137.194.69/phorestwww/ncbi.php?accno=38489215) | [5e -56](http://200.137.194.69/phorestwww/xmlparse.php?clone=10271&frame=2&accno=38489215#x) | 1 | 1 |
| Endonuclease III^b^ | [*Aspergillus nidulans*/XP_411790](http://200.137.194.69/phorestwww/ncbi.php?accno=40742649) | [1e -06](http://200.137.194.69/phorestwww/xmlparse.php?clone=11228&frame=1&accno=40742649#x) | - | 1 |
|  | ATPase involved in DNA repair | [*Aspergillus nidulans*/XP_409855](http://200.137.194.69/phorestwww/ncbi.php?accno=40743621) | [7e -86](http://200.137.194.69/phorestwww/xmlparse.php?clone=13023&frame=1&accno=40743621#x) | 1 | 1 |
|  | SGT1-like protein | [*Aspergillus nidulans*/XP_412397](http://200.137.194.69/phorestwww/ncbi.php?accno=40739808) | [4e -33](http://200.137.194.69/phorestwww/xmlparse.php?clone=10720&frame=3&accno=40739808#x) | 1 | 1 |
|  | Rad21 protein* | [*Neurospora crassa*/EAA34981](http://200.137.194.69/phorestwww/ncbi.php?accno=32420567) | [6e -17](http://200.137.194.69/phorestwww/xmlparse.php?clone=10884&frame=2&accno=32420567#x) | - | 2 |
|  | UV excision repair protein (rad23 homolog) | [*Aspergillus nidulans*/XP_406441](http://200.137.194.69/phorestwww/ncbi.php?accno=40745259) | [8e -25](http://200.137.194.69/phorestwww/xmlparse.php?clone=10414&frame=2&accno=40745259#x) | 17 | 1 |
|  | Prohibitin 2^a^ | [*Aspergillus nidulans*/XP_410210](http://200.137.194.69/phorestwww/ncbi.php?accno=40738858) | [6e -52](http://200.137.194.69/phorestwww/xmlparse.php?clone=10150&frame=1&accno=40738858#x) | 3 | 5 |
|  | Wos2 protein (p21) | [*Aspergillus nidulans*/XP_411058](http://200.137.194.69/phorestwww/ncbi.php?accno=40738486) | [1e -07](http://200.137.194.69/phorestwww/xmlparse.php?clone=10885&frame=1&accno=40738486#x) | 19 | 2 |
|  | Arrestin, N-terminal domain^b^ | [*Aspergillus nidulans*/XP_404193](http://200.137.194.69/phorestwww/ncbi.php?accno=40746078) | [2e -56](http://200.137.194.69/phorestwww/xmlparse.php?clone=10905&frame=1&accno=40746078#x) | - | 1 |
|  | Proliferating Cell Nuclear Antigen (PCNA)* | [*Aspergillus nidulans*/XP_404552](http://200.137.194.69/phorestwww/ncbi.php?accno=40747358) | [3e -36](http://200.137.194.69/phorestwww/xmlparse.php?clone=10782&frame=2&accno=40747358#x) | - | 1 |
|  | Single-stranded DNA binding protein | [*Magnaporthe grisea*/XP_369938](http://200.137.194.69/phorestwww/ncbi.php?accno=38110818) | [6e -31](http://200.137.194.69/phorestwww/xmlparse.php?clone=10621&frame=3&accno=38110818#x) | 1 | 1 |
|  | DNA polymerase delta 2 subunit^+^ | *Coccidioides immitis*/EAS28821 | 6e-33 | - | 2 |
|  | DNA polymerase eta^b^ | *Aspergillus nidulans*/XP_408815 | [1e -32](http://200.137.194.69/phorestwww/xmlparse.php?clone=18855&frame=3&accno=40741530#x) | - | 1 |
|  | Heterokaryon incompatibility protein Het-C^b^ | [*Aspergillus nidulans*/XP_406304](http://200.137.194.69/phorestwww/ncbi.php?accno=40745055) | [1e -27](http://200.137.194.69/phorestwww/xmlparse.php?clone=10413&frame=1&accno=40745055#x) | - | 2 |
|  | Uracil DNA glycosylase* | *Aspergillus fumigatus*/ XP_749743 | 3e -24 | - | 1 |
|  | Cell division protein 48 (CDC48)^a^ | *Coccidioides immitis* ***/***EAS28203 | 9e -15 | 2 | 3 |
|  | Chromosome segregation ATPase* | *Coccidioides immitis* **/**EAS30662 | 6e -52 | - | 1 |
|  | G2-specific protein kinase nimA | *Aspergillus terreus* **/**XP_001209874 | 9e -25 | 1 | 1 |
|  |  |  |  |  |  |
| **Transcription** | DEAD-like helicases superfamily protein*^#^ | [*Aspergillus nidulans*/XP_410144](http://200.137.194.69/phorestwww/ncbi.php?accno=40738566) | 3[e -55](http://200.137.194.69/phorestwww/xmlparse.php?clone=10424&frame=3&accno=40738566#x) | - | 1 |
|  | HLA-B associated transcript 4 | [*Aspergillus nidulans*/XP_408862](http://200.137.194.69/phorestwww/ncbi.php?accno=40741577) | [4e -09](http://200.137.194.69/phorestwww/xmlparse.php?clone=10700&frame=2&accno=40741577#x) | 2 | 1 |
|  | Transcription factor, bromodomain* | [*Aspergillus nidulans*/EAA60972](http://200.137.194.69/phorestwww/ncbi.php?accno=40741782) | [2e -55](http://200.137.194.69/phorestwww/xmlparse.php?clone=10495&frame=2&accno=40741782#x) | - | 1 |
|  | GatB/YqeY domain protein* | [*Aspergillus nidulans*/XP_410874](http://200.137.194.69/phorestwww/ncbi.php?accno=40739365) | [1e -22](http://200.137.194.69/phorestwww/xmlparse.php?clone=10286&frame=3&accno=40739365#x) | - | 1 |
|  | RNA polymerase I second-largest subunit | [*Aspergillus nidulans*/XP_408070](http://200.137.194.69/phorestwww/ncbi.php?accno=40740052) | [2e -36](http://200.137.194.69/phorestwww/xmlparse.php?clone=10391&frame=1&accno=40740052#x) | 1 | 1 |
|  | Pheromone receptor transcription factor | [*Aspergillus nidulans*/XP_412813](http://200.137.194.69/phorestwww/ncbi.php?accno=40740908) | [2e -32](http://200.137.194.69/phorestwww/xmlparse.php?clone=10234&frame=2&accno=40740908#x) | 1 | 1 |
|  | Zn(II)2Cys6 transcriptional activator | [*Aspergillus nidulans*/XP_408623](http://200.137.194.69/phorestwww/ncbi.php?accno=40741639) | [9e -32](http://200.137.194.69/phorestwww/xmlparse.php?clone=10671&frame=3&accno=40741639#x) | 1 | 1 |
|  | MATA_HMG-box, class I member of the HMG-box superfamily of DNA-binding proteins | [*Aspergillus nidulans*/XP_406099](http://200.137.194.69/phorestwww/ncbi.php?accno=40745971) | [3e -35](http://200.137.194.69/phorestwww/xmlparse.php?clone=6927&frame=2&accno=40745971#x) | 43 | 1 |
|  | trna pseudouridine synthase 4 | [*Aspergillus nidulans*/XP_404209](http://200.137.194.69/phorestwww/ncbi.php?accno=40746094) | [6e -12](http://200.137.194.69/phorestwww/xmlparse.php?clone=10352&frame=3&accno=40746094#x) | 2 | 1 |
|  | Small nuclear ribonucleoprotein D2 | [*Magnaporthe grisea*/XP_361539](http://200.137.194.69/phorestwww/ncbi.php?accno=38103571) | [1e -39](http://200.137.194.69/phorestwww/xmlparse.php?clone=10661&frame=3&accno=38103571#x) | 1 | 1 |
|  | snRNA-associated protein, Sm class^b^ | [*Magnaporthe grisea*/XP_368889](http://200.137.194.69/phorestwww/ncbi.php?accno=38101787) | [9e -32](http://200.137.194.69/phorestwww/xmlparse.php?clone=10279&frame=3&accno=38101787#x) | - | 1 |
|  | U6 snRNA-associated Sm-like protein LSm5^b^ | [*Neurospora crassa*](http://200.137.194.69/phorestwww/ncbi.php?accno=16944624)/CAD11394 | [4e -32](http://200.137.194.69/phorestwww/xmlparse.php?clone=9977&frame=1&accno=16944624#x) | - | 1 |
|  | U6 snRNA-associated Sm-like protein LSm6 | [*Gibberella zeae*/XP_380634](http://200.137.194.69/phorestwww/ncbi.php?accno=42545628) | [3e -27](http://200.137.194.69/phorestwww/xmlparse.php?clone=10210&frame=3&accno=42545628#x) | 1 | 1 |
|  | tRNA acetyltransferase TAN1 | [*Aspergillus nidulans*/XP_409026](http://200.137.194.69/phorestwww/ncbi.php?accno=40741777) | [1e -40](http://200.137.194.69/phorestwww/xmlparse.php?clone=10046&frame=3&accno=40741777#x) | 7 | 1 |
|  | tRNA (guanine) methyltransferase^b^ | [*Aspergillus nidulans*/XP_407082](http://200.137.194.69/phorestwww/ncbi.php?accno=40744340) | [3e -23](http://200.137.194.69/phorestwww/xmlparse.php?clone=10818&frame=1&accno=40744340#x) | - | 3 |
|  | Alanine-tRNA ligase | *Coccidioides immitis*/ EAS36927 | 2e -67 | 2 | 2 |
|  | Ring type Zinc finger protein* | [*Aspergillus nidulans*/XP_411042](http://200.137.194.69/phorestwww/ncbi.php?accno=40739114) | [1e -12](http://200.137.194.69/phorestwww/xmlparse.php?clone=10338&frame=1&accno=40739114#x) | - | 2 |
|  | Zinc finger domain protein* | [*Aspergillus nidulans*/XP_405585](http://200.137.194.69/phorestwww/ncbi.php?accno=40745422) | [3e -14](http://200.137.194.69/phorestwww/xmlparse.php?clone=10222&frame=1&accno=40745422#x) | - | 2 |
|  | Zinc finger domain protein | [*Magnaporthe grisea*/XP_370415](http://200.137.194.69/phorestwww/ncbi.php?accno=38109372) | [9e -06](http://200.137.194.69/phorestwww/xmlparse.php?clone=10771&frame=3&accno=38109372#x) | 1 | 1 |
|  | [Ap-1-like transcription factor](http://200.137.194.69/phorestwww/ncbi.php?accno=19114574)^b^ | *Aspergillus nidulans*/XP_411679 | [2e -30](http://200.137.194.69/phorestwww/xmlparse.php?clone=10993&frame=3&accno=40742932#x) | - | 1 |
|  | HAPE ([CCAAT-binding transcription factor subunit AAB-1](http://200.137.194.69/phorestwww/ncbi.php?accno=2583171)) | [*Aspergillus oryzae*](http://200.137.194.69/phorestwww/ncbi.php?accno=3059251)/BAA25636 | [2e -12](http://200.137.194.69/phorestwww/xmlparse.php?clone=10593&frame=2&accno=3059251#x) | 1 | 1 |
|  | RNA-binding protein | [*Aspergillus nidulans*/XP_407876](http://200.137.194.69/phorestwww/ncbi.php?accno=40740757) | [2e -36](http://200.137.194.69/phorestwww/xmlparse.php?clone=10853&frame=2&accno=40740757#x) | 1 | 1 |
|  | RNA-binding protein S1 | *Neurospora crassa*/EAA28780 | [1e -35](http://200.137.194.69/phorestwww/xmlparse.php?clone=7474&frame=3&accno=32418342#x) | 2 | 1 |
|  | RNA-binding protein | [*Coccidioides immitis*](http://200.137.194.69/phorestwww/ncbi.php?accno=15811426)/AAL08969 | [6e -20](http://200.137.194.69/phorestwww/xmlparse.php?clone=10026&frame=2&accno=15811426#x) | 8 | 1 |
|  | Transformer-SR ribonucleoprotein | [*Aspergillus nidulans*/XP_410813](http://200.137.194.69/phorestwww/ncbi.php?accno=40738429) | [1e -22](http://200.137.194.69/phorestwww/xmlparse.php?clone=6390&frame=3&accno=40738429#x) | 4 | 1 |
|  | Regulator of nonsense transcripts 1 homolog^b^ | *Neurospora crassa*/XP_323582 | [5e -15](http://200.137.194.69/phorestwww/xmlparse.php?clone=10469&frame=-2&accno=32405938#x) | - | 6 |
|  | Arylsulfatase regulatory protein* | *Blastocladiella emersonii*/CO964913 | 1e -138 | - | 11 |
|  | Transcriptional activator protein* | *Coccidioides immitis* **/**EAS34609 | 8e -26 | - | 1 |
|  | Nucleolar protein NOP56^a^ | *Coccidioides immitis* **/**EAS36543 | 8e -84 | 1 | 3 |
|  |  |  |  |  |  |
| **Protein Synthesis** | 40s ribosomal protein S2 | *Coccidioides immitis*/ EAS29348 | 6e -82 | 25 | 2 |
| 40S ribosomal protein S5^a^ | [*Aspergillus nidulans*/XP_404980](http://200.137.194.69/phorestwww/ncbi.php?accno=40746517) | [8e -22](http://200.137.194.69/phorestwww/xmlparse.php?clone=10658&frame=3&accno=40746517#x) | 8 | 7 |
|  | 40s ribosomal protein S8 | [*Aspergillus nidulans*/XP_404602](http://200.137.194.69/phorestwww/ncbi.php?accno=40747408) | [5e -55](http://200.137.194.69/phorestwww/xmlparse.php?clone=10149&frame=2&accno=40747408#x) | 13 | 2 |
|  | 40S ribosomal protein S9 | [*Aspergillus nidulans*/XP_408940](http://200.137.194.69/phorestwww/ncbi.php?accno=40741183) | [1e -87](http://200.137.194.69/phorestwww/xmlparse.php?clone=14352&frame=2&accno=40741183#x) | 1 | 1 |
|  | 40S ribosomal protein S11B | [*Gibberella zeae*/XP_380847](http://200.137.194.69/phorestwww/ncbi.php?accno=42544489) | [2e -69](http://200.137.194.69/phorestwww/xmlparse.php?clone=10769&frame=1&accno=42544489#x) | 2 | 1 |
|  | 40S ribosomal protein S12 | [*Paracoccidioides brasiliensis*/](http://200.137.194.69/phorestwww/ncbi.php?accno=28395450)AAO38980 | [2e -82](http://200.137.194.69/phorestwww/xmlparse.php?clone=10423&frame=2&accno=28395450#x) | 15 | 1 |
|  | 40S ribosomal protein S13^b^ | [*Neurospora crassa*/EAA34807](http://200.137.194.69/phorestwww/ncbi.php?accno=32419563) | [2e -37](http://200.137.194.69/phorestwww/xmlparse.php?clone=10367&frame=1&accno=32419563#x) | - | 1 |
|  | 40S ribosomal protein S19B | [*Aspergillus nidulans* /EAA58948](http://200.137.194.69/phorestwww/ncbi.php?accno=40739758) | [3e -30](http://200.137.194.69/phorestwww/xmlparse.php?clone=10231&frame=2&accno=40739758#x) | 3 | 1 |
|  | 40s ribosomal protein S21e | *Coccidioides immitis*/ EAS33475 | 2e -11 | 8 | 2 |
|  | 40S ribosomal protein S26^b^ | [*Neurospora crassa*/CAA39162](http://200.137.194.69/phorestwww/ncbi.php?accno=3076) | [3e -52](http://200.137.194.69/phorestwww/xmlparse.php?clone=10870&frame=1&accno=3076#x) | - | 1 |
|  | 40S ribosomal protein S30A | [*Gibberella zeae*/XP_385091](http://200.137.194.69/phorestwww/ncbi.php?accno=42551437) | [1e -18](http://200.137.194.69/phorestwww/xmlparse.php?clone=10202&frame=1&accno=42551437#x) | 16 | 1 |
|  | 60S ribosomal protein L1B | [*Aspergillus fumigatus*](http://200.137.194.69/phorestwww/ncbi.php?accno=41581246)/CAE47895 | [8e -55](http://200.137.194.69/phorestwww/xmlparse.php?clone=10181&frame=2&accno=41581246#x) | 10 | 1 |
|  | 60S ribosomal protein L2^b^ | *Coccidioides immitis*/EAS30555 | 9e -54 | - | 1 |
|  | 60S ribosomal protein L3^a^ | [*Aspergillus fumigatus*](http://200.137.194.69/phorestwww/ncbi.php?accno=21215170)/AAM43909 | [5e -85](http://200.137.194.69/phorestwww/xmlparse.php?clone=9978&frame=3&accno=21215170#x) | 1 | 2 |
|  | 60S ribosomal protein L7, mitochondrial precursor | [*Aspergillus nidulans*/XP_406194](http://200.137.194.69/phorestwww/ncbi.php?accno=40745733) | [7e -32](http://200.137.194.69/phorestwww/xmlparse.php?clone=10725&frame=3&accno=40745733#x) | 1 | 1 |
|  | 60s ribosomal protein L12 | [*Aspergillus nidulans*/XP_404399](http://200.137.194.69/phorestwww/ncbi.php?accno=40746979) | [1e -43](http://200.137.194.69/phorestwww/xmlparse.php?clone=10100&frame=2&accno=40746979#x) | 1 | 1 |
|  | 60S ribosomal protein L15B | [*Neurospora crassa*/CAD21192](http://200.137.194.69/phorestwww/ncbi.php?accno=32415471) | [3e -40](http://200.137.194.69/phorestwww/xmlparse.php?clone=10353&frame=1&accno=32415471#x) | 2 | 2 |
|  | 60S ribosomal protein L17 | [*Paracoccidioides brasiliensis*/](http://200.137.194.69/phorestwww/ncbi.php?accno=33316745)AAQ04632 | [1e -67](http://200.137.194.69/phorestwww/xmlparse.php?clone=10842&frame=2&accno=33316745#x) | 5 | 3 |
|  | 60S ribosomal protein L20^a^ | [*Magnaporthe grisea*/XP_361110](http://200.137.194.69/phorestwww/ncbi.php?accno=38105659) | [3e -16](http://200.137.194.69/phorestwww/xmlparse.php?clone=10740&frame=2&accno=38105659#x) | 3 | 6 |
|  | 60S ribosomal protein L20B | [*Gibberella zeae*/XP_381692](http://200.137.194.69/phorestwww/ncbi.php?accno=42546058) | [4e -49](http://200.137.194.69/phorestwww/xmlparse.php?clone=10039&frame=3&accno=42546058#x) | 1 | 1 |
|  | 60 ribosomal protein L23A | [*Neurospora crassa*/EAA33841](http://200.137.194.69/phorestwww/ncbi.php?accno=32411201) | [8e -53](http://200.137.194.69/phorestwww/xmlparse.php?clone=10377&frame=2&accno=32411201#x) | 3 | 1 |
|  | 60S ribosomal protein L23, mitochondrial precursor | [*Aspergillus nidulans*/XP_413606](http://200.137.194.69/phorestwww/ncbi.php?accno=40738397) | [2e -39](http://200.137.194.69/phorestwww/xmlparse.php?clone=10490&frame=2&accno=40738397#x) | 1 | 1 |
|  | 60s ribosomal protein L26 | *Schizosaccharomyces pombe*/ CAD37159 | 2e -30 | 3 | 2 |
|  | 60S ribosomal protein L27^a^ | [*Aspergillus nidulans*/XP_408359](http://200.137.194.69/phorestwww/ncbi.php?accno=40740131) | [4e -63](http://200.137.194.69/phorestwww/xmlparse.php?clone=10322&frame=2&accno=40740131#x) | 1 | 3 |
|  | 60s ribosomal protein L28 | *Coccidioides immitis*/ EAS37227 | 2e -36 | 9 | 4 |
|  | 60S ribosomal protein L29 | [*Neurospora crassa*/EAA28550](http://200.137.194.69/phorestwww/ncbi.php?accno=32403576) | [1e -27](http://200.137.194.69/phorestwww/xmlparse.php?clone=10533&frame=1&accno=32403576#x) | 2 | 1 |
|  | 60S ribosomal protein L36 | [*Gibberella zeae*/XP_381414](http://200.137.194.69/phorestwww/ncbi.php?accno=42545256) | [3e -33](http://200.137.194.69/phorestwww/xmlparse.php?clone=10711&frame=3&accno=42545256#x) | 6 | 1 |
|  | 60S ribosomal protein L43B^b^ | [*Ustilago maydis*/XP_400133](http://200.137.194.69/phorestwww/ncbi.php?accno=46098416) | [1e -30](http://200.137.194.69/phorestwww/xmlparse.php?clone=10600&frame=1&accno=46098416#x) | - | 1 |
|  | 60S acidic ribosomal protein P2 | *Aspergillus fumigatus*/AAG01801 | [2e -21](http://200.137.194.69/phorestwww/xmlparse.php?clone=11630&frame=2&accno=9887212#x) | 26 | 3 |
|  | 60S ribosome subunit biogenesis protein NIP7^b^ | [*Aspergillus fumigatus*](http://200.137.194.69/phorestwww/ncbi.php?accno=28394453)/AAM08680 | [3e -14](http://200.137.194.69/phorestwww/xmlparse.php?clone=10816&frame=3&accno=28394453#x) | - | 1 |
|  | Ribosomal protein P1 | [*Neurospora crassa*/EAA31448](http://200.137.194.69/phorestwww/ncbi.php?accno=32421817) | [7e -22](http://200.137.194.69/phorestwww/xmlparse.php?clone=10736&frame=3&accno=32421817#x) | 15 | 4 |
|  | Ribosomal protein L4B | [*Aspergillus nidulans*/XP_412313](http://200.137.194.69/phorestwww/ncbi.php?accno=40740008) | [3e -60](http://200.137.194.69/phorestwww/xmlparse.php?clone=10498&frame=2&accno=40740008#x) | 2 | 1 |
|  | Ribosomal protein L10 | [*Paracoccidioides brasiliensis*/](http://200.137.194.69/phorestwww/ncbi.php?accno=28797723)AAO47090 | [4e -60](http://200.137.194.69/phorestwww/xmlparse.php?clone=9990&frame=1&accno=28797723#x) | 1 | 1 |
|  | Ribosomal protein L21A | [Aspergillus nidulans/XP_408939](http://200.137.194.69/phorestwww/ncbi.php?accno=40741182) | [1e -54](http://200.137.194.69/phorestwww/xmlparse.php?clone=9958&frame=3&accno=40741182#x) | 3 | 2 |
|  | Ribosomal protein L31A | [*Aspergillus nidulans*/XP_409623](http://200.137.194.69/phorestwww/ncbi.php?accno=40743456) | [3e -51](http://200.137.194.69/phorestwww/xmlparse.php?clone=10148&frame=1&accno=40743456#x) | 6 | 1 |
|  | Ribosomal protein L34B | [*Aspergillus nidulans*/XP_408659](http://200.137.194.69/phorestwww/ncbi.php?accno=40741675) | [5e -54](http://200.137.194.69/phorestwww/xmlparse.php?clone=10829&frame=1&accno=40741675#x) | 9 | 3 |
|  | Ribosomal protein L37 | [*Emericella nidulan*/AAK17097](http://200.137.194.69/phorestwww/ncbi.php?accno=13272327) | [1e -34](http://200.137.194.69/phorestwww/xmlparse.php?clone=10535&frame=1&accno=13272327#x) | 2 | 1 |
|  | Mitochondrial ribosomal protein S19^b^ | [*Aspergillus nidulans*/XP_404292](http://200.137.194.69/phorestwww/ncbi.php?accno=40746872) | [5e -19](http://200.137.194.69/phorestwww/xmlparse.php?clone=10246&frame=2&accno=40746872#x) | - | 1 |
|  | Mitochondrial 40S ribosomal protein MRP17 | [*Aspergillus fumigatus*](http://200.137.194.69/phorestwww/ncbi.php?accno=41581285)/CAE47934 | [5e -35](http://200.137.194.69/phorestwww/xmlparse.php?clone=10269&frame=3&accno=41581285#x) | 1 | 1 |
|  | Mitochondrial large ribosomal subunit | [*Neurospora crassa*/](http://200.137.194.69/phorestwww/ncbi.php?accno=32405764)XP_323495 | [3e -05](http://200.137.194.69/phorestwww/xmlparse.php?clone=10793&frame=2&accno=32405764#x) | 1 | 1 |
|  | 14 kDa mitochondrial ribosomal protein* | [*Aspergillus nidulans*/XP_408748](http://200.137.194.69/phorestwww/ncbi.php?accno=40741223) | [4e -46](http://200.137.194.69/phorestwww/xmlparse.php?clone=10336&frame=3&accno=40741223#x) | - | 7 |
|  | Eukaryotic initiation factor 4A | [*Aspergillus nidulans*/XP_407069](http://200.137.194.69/phorestwww/ncbi.php?accno=40744327) | [5e -51](http://200.137.194.69/phorestwww/xmlparse.php?clone=10129&frame=2&accno=40744327#x) | 9 | 2 |
|  | Translational machinery component protein^b^ | [*Aspergillus nidulans*/XP_405417](http://200.137.194.69/phorestwww/ncbi.php?accno=40746717) | [1e -19](http://200.137.194.69/phorestwww/xmlparse.php?clone=10837&frame=3&accno=40746717#x) | - | 1 |
|  | Translation initiation factor eIF1 | [*Aspergillus nidulans*/XP_408879](http://200.137.194.69/phorestwww/ncbi.php?accno=40741594) | [8e -24](http://200.137.194.69/phorestwww/xmlparse.php?clone=10095&frame=1&accno=40741594#x) | 4 | 2 |
|  | Translation initiation factor eIF1 subunit Sui1^a^ | [*Gibberella zeae*/XP_389056](http://200.137.194.69/phorestwww/ncbi.php?accno=42549211) | [2e -36](http://200.137.194.69/phorestwww/xmlparse.php?clone=10370&frame=3&accno=42549211#x) | 2 | 5 |
|  | Translation initiation factor eIF-5A^a^ | *Neurospora crassa*/P38672 | [6e -06](http://200.137.194.69/phorestwww/xmlparse.php?clone=10406&frame=1&accno=729823#x) | 4 | 4 |
|  | Translation initiation factor 3 subunit 2* | *Aspergillus nidulans* **/**XP_660601 | 6e -80 | - | 3 |
|  | Translation initiation factor 3 subunit 4 | [*Aspergillus nidulans*/XP_410154](http://200.137.194.69/phorestwww/ncbi.php?accno=40738468) | [3e -40](http://200.137.194.69/phorestwww/xmlparse.php?clone=10116&frame=3&accno=40738468#x) | 2 | 1 |
|  | Translational elongation factor EF-1 alpha | [*Aspergillus nidulans*/XP_405299](http://200.137.194.69/phorestwww/ncbi.php?accno=40747124) | [4e -31](http://200.137.194.69/phorestwww/xmlparse.php?clone=10416&frame=1&accno=40747124#x) | 28 | 8 |
|  | Translation elongation factor 2 | [*Gibberella zeae*/EAA77131](http://200.137.194.69/phorestwww/ncbi.php?accno=42554288) | [1e -57](http://200.137.194.69/phorestwww/xmlparse.php?clone=10010&frame=1&accno=42554288#x) | 3 | 2 |
|  | Translation elongation factor Tu, mitochondrial | [*Aspergillus fumigatus*/](http://200.137.194.69/phorestwww/ncbi.php?accno=19309398)CAD27297 | [1e -14](http://200.137.194.69/phorestwww/xmlparse.php?clone=10553&frame=3&accno=19309398#x) | 1 | 1 |
|  | Isoleucyl-tRNA synthetase^b^ | [*Aspergillus nidulans*/XP_407499](http://200.137.194.69/phorestwww/ncbi.php?accno=40744150) | [1e -52](http://200.137.194.69/phorestwww/xmlparse.php?clone=10058&frame=2&accno=40744150#x) | - | 2 |
|  | Peptide chain release factor 2 | [*Bordetella parapertussis*/CAE37347](http://200.137.194.69/phorestwww/ncbi.php?accno=33596662) | [3e -71](http://200.137.194.69/phorestwww/xmlparse.php?clone=10786&frame=2&accno=33596662#x) | 2 | 1 |
|  | GTP-binding GTP1/OBG family protein^b^ | [*Aspergillus nidulans*/XP_404829](http://200.137.194.69/phorestwww/ncbi.php?accno=40746312) | [1e -70](http://200.137.194.69/phorestwww/xmlparse.php?clone=10302&frame=2&accno=40746312#x) | - | 1 |
|  |  |  |  |  |  |
| **Protein fate** | Protein disulfide isomerase | [*Gibberella zeae*/XP_389492](http://200.137.194.69/phorestwww/ncbi.php?accno=42553220) | [1e -43](http://200.137.194.69/phorestwww/xmlparse.php?clone=9951&frame=2&accno=42553220#x) | 4 | 3 |
|  | Cyclophilin like peptidyl prolyl cis-trans isomerase^b^ | [*Neurospora crassa*](http://200.137.194.69/phorestwww/ncbi.php?accno=18376309)/CAD21421 | [8e -39](http://200.137.194.69/phorestwww/xmlparse.php?clone=10030&frame=3&accno=18376309#x) | - | 1 |
|  | Cyclophilin seven suppressor 1^b^ | [*Aspergillus nidulans*/XP_409575](http://200.137.194.69/phorestwww/ncbi.php?accno=40743408) | [8e -12](http://200.137.194.69/phorestwww/xmlparse.php?clone=10243&frame=1&accno=40743408#x) | - | 2 |
|  | Peptidyl-prolyl cis-trans isomerase H | [*Gibberella zeae*/XP_381060](http://200.137.194.69/phorestwww/ncbi.php?accno=42547634) | [5e -55](http://200.137.194.69/phorestwww/xmlparse.php?clone=10565&frame=3&accno=42547634#x) | 8 | 1 |
|  | Peptidyl-prolyl cis-trans isomerase (FKBP-type) | [*Magnaporthe grisea*/XP_362031](http://200.137.194.69/phorestwww/ncbi.php?accno=38104100) | [1e -44](http://200.137.194.69/phorestwww/xmlparse.php?clone=10802&frame=1&accno=38104100#x) | 2 | 2 |
|  | Peptidyl-prolyl cis-trans isomerase E | [*Aspergillus nidulans*/XP_410393](http://200.137.194.69/phorestwww/ncbi.php?accno=40739450) | [3e -33](http://200.137.194.69/phorestwww/xmlparse.php?clone=10305&frame=1&accno=40739450#x) | 2 | 1 |
|  | Peptidyl-prolyl cis-trans isomerase (cyclophilin-2) | [*Aspergillus nidulans*/XP_412817](http://200.137.194.69/phorestwww/ncbi.php?accno=40740912) | [2e -41](http://200.137.194.69/phorestwww/xmlparse.php?clone=10264&frame=1&accno=40740912#x) | 11 | 1 |
|  | Peptidyl-prolyl cis/trans isomerase (PPIC-type) | [*Paracoccidioides brasiliensis*/](http://200.137.194.69/phorestwww/ncbi.php?accno=34979129)AAQ83700 | [4e -38](http://200.137.194.69/phorestwww/xmlparse.php?clone=10290&frame=2&accno=34979129#x) | 6 | 1 |
|  | Peptidyl-prolyl cis-trans isomerase-like 4^a^ | *Coccidioides immitis*/ EAS29016 | 1e -46 | 1 | 5 |
|  | SNF7 domain protein | [*Aspergillus nidulans*/XP_409769](http://200.137.194.69/phorestwww/ncbi.php?accno=40743535) | [1e -52](http://200.137.194.69/phorestwww/xmlparse.php?clone=10850&frame=3&accno=40743535#x) | 2 | 2 |
|  | Rab geranylgeranyl transferase* | [*Aspergillus nidulans*/XP_412816](http://200.137.194.69/phorestwww/ncbi.php?accno=40740911) | [8e -13](http://200.137.194.69/phorestwww/xmlparse.php?clone=10666&frame=1&accno=40740911#x) | - | 1 |
|  | Protein-L-isoaspartate(D-aspartate)O-methyltransferase^a^ | [*Aspergillus nidulans*/XP_407601](http://200.137.194.69/phorestwww/ncbi.php?accno=40743818) | [5e -55](http://200.137.194.69/phorestwww/xmlparse.php?clone=18843&frame=-1&accno=40743818#x) | 4 | 5 |
|  | COP9 signalosome complex subunit 5^a^ | [*Aspergillus nidulans*/XP_406266](http://200.137.194.69/phorestwww/ncbi.php?accno=40745805) | [1e -35](http://200.137.194.69/phorestwww/xmlparse.php?clone=10063&frame=3&accno=40745805#x) | 1 | 2 |
|  | COP9 signalosome complex subunit 7a | [*Aspergillus nidulans*/XP_407760](http://200.137.194.69/phorestwww/ncbi.php?accno=40740641) | [1e -35](http://200.137.194.69/phorestwww/xmlparse.php?clone=4115&frame=1&accno=40740641#x) | 3 | 1 |
|  | Palmitoyl thioesterase protein | [*Aspergillus nidulans*/XP_406634](http://200.137.194.69/phorestwww/ncbi.php?accno=40744826) | [4e -12](http://200.137.194.69/phorestwww/xmlparse.php?clone=10855&frame=1&accno=40744826#x) | 4 | 1 |
|  | Oligosaccharyltransferase alpha subunit | [*Aspergillus niger*/AAK08631](http://200.137.194.69/phorestwww/ncbi.php?accno=13027697) | [2e -23](http://200.137.194.69/phorestwww/xmlparse.php?clone=10426&frame=3&accno=13027697#x) | 3 | 1 |
|  | Guanosine diphosphatase*^#^ | [*Aspergillus nidulans*/XP_405219](http://200.137.194.69/phorestwww/ncbi.php?accno=40747044) | [2e -15](http://200.137.194.69/phorestwww/xmlparse.php?clone=10356&frame=1&accno=40747044#x) | - | 1 |
|  | N-acetyltransferase | [*Aspergillus nidulans*/XP_409130](http://200.137.194.69/phorestwww/ncbi.php?accno=40741881) | [3e -17](http://200.137.194.69/phorestwww/xmlparse.php?clone=10738&frame=1&accno=40741881#x) | 1 | 1 |
|  | Ubiquitin fusion protein^a^ | [*Schizosaccharomyces pombe*/NP_593923](http://200.137.194.69/phorestwww/ncbi.php?accno=19115310) | [8e -67](http://200.137.194.69/phorestwww/xmlparse.php?clone=20004&frame=3&accno=19115310#x) | 3 | 3 |
|  | Ubiquitin thiolesterase otubain like protein* | [*Aspergillus nidulans*/ EAA60354](http://200.137.194.69/phorestwww/ncbi.php?accno=40741164) | [1e -28](http://200.137.194.69/phorestwww/xmlparse.php?clone=10493&frame=3&accno=40741164#x) | - | 1 |
|  | Ubiquitin conjugating enzyme E2^a^ | [*Gibberella zeae*/XP_388490](http://200.137.194.69/phorestwww/ncbi.php?accno=42547969) | [1e -29](http://200.137.194.69/phorestwww/xmlparse.php?clone=10639&frame=1&accno=42547969#x) | 6 | 7 |
|  | [Ubiquitin/S27a fusion protein](http://200.137.194.69/phorestwww/ncbi.php?accno=11275235)^a^ | [*Aspergillus nidulans*/XP_409009](http://200.137.194.69/phorestwww/ncbi.php?accno=40741760) | [2e -60](http://200.137.194.69/phorestwww/xmlparse.php?clone=10589&frame=3&accno=40741760#x) | 7 | 7 |
|  | Ubiquitin-protein ligase (HUL4) | *Coccidioides immitis* **/**EAS31641 | 4e -88 | 1 | 1 |
|  | Polyubiquitin [ubi4](http://200.137.194.69/phorestwww/ncbi.php?accno=19112201) | [*Arabidopsis thaliana/*](http://200.137.194.69/phorestwww/ncbi.php?accno=2760349)AAB95252 | [1e -48](http://200.137.194.69/phorestwww/xmlparse.php?clone=10071&frame=2&accno=2760349#x) | 4 | 3 |
|  | Lon protease^b^ | *Oryza sativa/*[AAV59316](http://www.ncbi.nlm.nih.gov/entrez/query.fcgi?cmd=Retrieve&db=Protein&list_uids=55733809&dopt=GenPept) | 1e -05 | - | 1 |
|  | Zinc metalloprotease^b^ | [*Neurospora crassa*](http://200.137.194.69/phorestwww/ncbi.php?accno=18376099)/CAD21161 | [3e -47](http://200.137.194.69/phorestwww/xmlparse.php?clone=10827&frame=1&accno=18376099#x) | - | 1 |
|  | ATP-dependent Clp protease, proteolytic subunit | [*Magnaporthe grisea*/XP_370260](http://200.137.194.69/phorestwww/ncbi.php?accno=38109190) | [4e -37](http://200.137.194.69/phorestwww/xmlparse.php?clone=10303&frame=3&accno=38109190#x) | 1 | 1 |
|  | Aspartyl protease^a^ | [*Paracoccidioides brasiliensis*](http://200.137.194.69/phorestwww/ncbi.php?accno=30575834)/AAP32823 | [3e -72](http://200.137.194.69/phorestwww/xmlparse.php?clone=10126&frame=2&accno=30575834#x) | 3 | 7 |
|  | 26S protease subunit protein^b^ | [*Aspergillus nidulans*/XP_411125](http://200.137.194.69/phorestwww/ncbi.php?accno=40742444) | [4e -23](http://200.137.194.69/phorestwww/xmlparse.php?clone=10421&frame=2&accno=40742444#x) | - | 1 |
|  | Non-ATPase regulatory subunit of the 26S proteasome* | [*Aspergillus nidulans*/XP_408912](http://200.137.194.69/phorestwww/ncbi.php?accno=40741155) | [2e -68](http://200.137.194.69/phorestwww/xmlparse.php?clone=10616&frame=2&accno=40741155#x) | - | 1 |
|  | 26S proteasome regulatory complex component protein | [*Aspergillus nidulans*/XP_408920](http://200.137.194.69/phorestwww/ncbi.php?accno=40741163) | [2e -73](http://200.137.194.69/phorestwww/xmlparse.php?clone=10701&frame=1&accno=40741163#x) | 3 | 1 |
|  | 26s proteasome regulatory subunit rpn12^b^ | [*Aspergillus nidulans*/XP_407156](http://200.137.194.69/phorestwww/ncbi.php?accno=40744414) | [5e -30](http://200.137.194.69/phorestwww/xmlparse.php?clone=10450&frame=3&accno=40744414#x) | - | 1 |
|  | Proteasome subunit alpha type 1 | [*Aspergillus nidulans*/XP_410684](http://200.137.194.69/phorestwww/ncbi.php?accno=40738697) | [5e -65](http://200.137.194.69/phorestwww/xmlparse.php?clone=10508&frame=1&accno=40738697#x) | 4 | 1 |
|  | Proteasome subunit alpha type 6 | *Candida albicans*/XP_717755 | [1e -24](http://200.137.194.69/phorestwww/xmlparse.php?clone=10250&frame=3&accno=49652372#x) | 1 | 1 |
|  | F-box/LRR-repeat protein 7^b^ | [*Aspergillus nidulans*/XP_408647](http://200.137.194.69/phorestwww/ncbi.php?accno=40741663) | [8e -28](http://200.137.194.69/phorestwww/xmlparse.php?clone=10531&frame=3&accno=40741663#x) | - | 3 |
|  | Calnexin | [*Aspergillus nidulans*/XP_407729](http://200.137.194.69/phorestwww/ncbi.php?accno=40740610) | [5e -63](http://200.137.194.69/phorestwww/xmlparse.php?clone=10592&frame=1&accno=40740610#x) | 5 | 1 |
|  | Peptidase C19 subfamily protein^b^ | [*Aspergillus nidulans*/XP_412211](http://200.137.194.69/phorestwww/ncbi.php?accno=40740506) | [7e -08](http://200.137.194.69/phorestwww/xmlparse.php?clone=10809&frame=2&accno=40740506#x) | - | 3 |
|  | Peptidase M28 domain protein* | *Coccidioides immitis*/EAS33583 | 1e -22 | - | 1 |
|  | Alpha-1, 2-galactosyltransferase*^#^ | [*Aspergillus nidulans*/XP_406106](http://200.137.194.69/phorestwww/ncbi.php?accno=40745978) | [3e -14](http://200.137.194.69/phorestwww/xmlparse.php?clone=10841&frame=1&accno=40745978#x) | - | 1 |
|  | Mitochondrial processing peptidase subunit | *Coccidioides immitis* **/**EAS32113 | 2e -40 | 3 | 1 |
|  | Oligosaccharyltransferase subunit ribophorin^+^ | *Coccidioides immitis*/ EAS29547 | 9e -37 | - | 1 |
|  | Tailless Complex Polypeptide 1 chaperonin, subunit epsilon^b^ | *Schizosaccharomyces pombe*/EAA65069 | 6e -16 | - | 2 |
|  | Mannosyltransferase^b^ | [*Paracoccidioides brasiliensis*](http://200.137.194.69/phorestwww/ncbi.php?accno=14161489)/AAK54761 | [3e -70](http://200.137.194.69/phorestwww/xmlparse.php?clone=10220&frame=1&accno=14161489#x) | - | 1 |
|  | Alpha-1, 2-mannosyltransferase^a^ | [*Neurospora crassa*/CAC18268](http://200.137.194.69/phorestwww/ncbi.php?accno=32412802) | [1e -29](http://200.137.194.69/phorestwww/xmlparse.php?clone=10332&frame=1&accno=32412802#x) | 3 | 3 |
|  | Ring (Really Interesting New Gene) type zinc finger (C3HC4) protein^b^ | [*Schizosaccharomyces pombe*/CAB08748](http://200.137.194.69/phorestwww/ncbi.php?accno=19114241) | [5e -10](http://200.137.194.69/phorestwww/xmlparse.php?clone=19931&frame=2&accno=19114241#x) | - | 1 |
|  |  |  |  |  |  |
| **Protein with binding function or cofactor requirement** |  |  |  |  |  |
| RPEL repeat protein^a^ | [*Aspergillus nidulans*/XP_407503.1](http://200.137.194.69/phorestwww/ncbi.php?accno=40744154) | [5e -22](http://200.137.194.69/phorestwww/xmlparse.php?clone=10591&frame=2&accno=40744154#x) | 1 | 3 |
|  |  |  |  |  |
|  |  |  |  |  |  |
| **Transport Facilitation** | Mitochondrial carrier protein Ggc1 fragment | [*Aspergillus nidulans*/XP_409269](http://200.137.194.69/phorestwww/ncbi.php?accno=40743123) | [5e -69](http://200.137.194.69/phorestwww/xmlparse.php?clone=10496&frame=2&accno=40743123#x) | 1 | 1 |
| Mitochondrial carrier protein^b^ | [*Gibberella zeae*/XP_391004](http://200.137.194.69/phorestwww/ncbi.php?accno=42552339) | [6e -22](http://200.137.194.69/phorestwww/xmlparse.php?clone=10260&frame=1&accno=42552339#x) | - | 3 |
|  | Xanthine/uracil/vitamin C permease^+^ | [*Magnaporthe grisea*/XP_362769](http://200.137.194.69/phorestwww/ncbi.php?accno=38111793) | [7e -34](http://200.137.194.69/phorestwww/xmlparse.php?clone=10481&frame=1&accno=38111793#x) | - | 1 |
|  | Sugar (and other) transporter protein | *Aspergillus nidulans*/XP_410859 | [8e -80](http://200.137.194.69/phorestwww/xmlparse.php?clone=969&frame=2&accno=40739350#x) | 12 | 3 |
|  | Sugar transporter protein^a^ | [*Gibberella zeae*/XP_381006](http://200.137.194.69/phorestwww/ncbi.php?accno=42547580) | [9e -55](http://200.137.194.69/phorestwww/xmlparse.php?clone=10128&frame=2&accno=42547580#x) | 3 | 5 |
|  | Copper transporter family protein^a^ | [*Gibberella zeae*/XP_380949](http://200.137.194.69/phorestwww/ncbi.php?accno=42547876) | [1e -28](http://200.137.194.69/phorestwww/xmlparse.php?clone=10912&frame=1&accno=42547876#x) | 4 | 8 |
|  | Copper transport protein-CTR2 | [*Aspergillus nidulans*/XP_407071](http://200.137.194.69/phorestwww/ncbi.php?accno=40744329) | [6e -13](http://200.137.194.69/phorestwww/xmlparse.php?clone=10087&frame=3&accno=40744329#x) | 2 | 1 |
|  | Sulfate permease^b^ | [*Gibberella zeae*/XP_384418](http://200.137.194.69/phorestwww/ncbi.php?accno=42550725) | [4e -46](http://200.137.194.69/phorestwww/xmlparse.php?clone=10823&frame=2&accno=42550725#x) | - | 1 |
|  | Glucose transporter | *Aspergillus fumigatus*/[XP_754226](http://www.ncbi.nlm.nih.gov/entrez/query.fcgi?cmd=Retrieve&db=Protein&list_uids=70999009&dopt=GenPept) | 2e -28 | 1 | 1 |
|  | Arabinose efflux permease | *Aspergillus nidulans*/XP_407577 | [6e -18](http://200.137.194.69/phorestwww/xmlparse.php?clone=3114&frame=1&accno=40743708#x) | 4 | 1 |
|  | Uridine diphosphate N-Acetylglucosamine transporter*^#^ | [*Neurospora crassa*](http://200.137.194.69/phorestwww/ncbi.php?accno=11359692)/T50997 | [9e -30](http://200.137.194.69/phorestwww/xmlparse.php?clone=10385&frame=3&accno=11359692#x) | - | 1 |
|  | Monossaccharide transport protein^b^ | [*Aspergillus nidulans*/XP_408414](http://200.137.194.69/phorestwww/ncbi.php?accno=40739625) | [8e -55](http://200.137.194.69/phorestwww/xmlparse.php?clone=10362&frame=3&accno=40739625#x) | - | 1 |
|  | Amino acid permease | [*Neurospora crassa*/](http://200.137.194.69/phorestwww/ncbi.php?accno=32404308)XP_322767 | [1e -23](http://200.137.194.69/phorestwww/xmlparse.php?clone=10792&frame=1&accno=32404308#x) | 8 | 2 |
|  | Acidic amino acid permease^a^ | [*Aspergillus nidulans*/XP_410255](http://200.137.194.69/phorestwww/ncbi.php?accno=40738903) | [2e -17](http://200.137.194.69/phorestwww/xmlparse.php?clone=9960&frame=1&accno=40738903#x) | 9 | 9 |
|  | B-cell receptor-associated protein 31-like^+^ | [*Aspergillus nidulans*/XP_404956](http://200.137.194.69/phorestwww/ncbi.php?accno=40746493) | [9e -43](http://200.137.194.69/phorestwww/xmlparse.php?clone=10557&frame=2&accno=40746493#x) | - | 1 |
|  | ATP synthase subunit 4 | [*Paracoccidioides brasiliensis*/](http://200.137.194.69/phorestwww/ncbi.php?accno=30351150)AAP22959 | [2e -79](http://200.137.194.69/phorestwww/xmlparse.php?clone=10261&frame=1&accno=30351150#x) | 6 | 2 |
|  | Purine nucleoside permease | [*Aspergillus nidulans*/XP_409674](http://200.137.194.69/phorestwww/ncbi.php?accno=40743507) | [2e -05](http://200.137.194.69/phorestwww/xmlparse.php?clone=10077&frame=-1&accno=40743507#x) | 1 | 1 |
|  | Malate permease^b^ | [*Gibberella zeae*/XP_389995](http://200.137.194.69/phorestwww/ncbi.php?accno=42554838) | [2e -25](http://200.137.194.69/phorestwww/xmlparse.php?clone=9995&frame=1&accno=42554838#x) | - | 2 |
|  | Nuclear pore protein 84/107* | [*Aspergillus nidulans*/XP_405327](http://200.137.194.69/phorestwww/ncbi.php?accno=40746627) | [3e -07](http://200.137.194.69/phorestwww/xmlparse.php?clone=10617&frame=2&accno=40746627#x) | - | 1 |
|  | Mitochondrial import receptor subunit Tom20^b^ | [*Aspergillus nidulans*/XP_404696](http://200.137.194.69/phorestwww/ncbi.php?accno=40747502) | [9e -40](http://200.137.194.69/phorestwww/xmlparse.php?clone=10335&frame=3&accno=40747502#x) | - | 1 |
|  | Mitochondrial RNA splicing protein mrs3 | [*Aspergillus nidulans*/XP_407827](http://200.137.194.69/phorestwww/ncbi.php?accno=40740708) | [1e -45](http://200.137.194.69/phorestwww/xmlparse.php?clone=9994&frame=3&accno=40740708#x) | 3 | 1 |
|  | ADP, ATP carrier-like protein | *Aspergillus nidulans*/EAA58952 | [3e -101](http://200.137.194.69/phorestwww/xmlparse.php?clone=13230&frame=1&accno=40739762#x) | 1 | 1 |
|  | Coatomer protein complex, subunit epsilon | [*Magnaporthe grisea*/XP_367110](http://200.137.194.69/phorestwww/ncbi.php?accno=38111045) | [8e -13](http://200.137.194.69/phorestwww/xmlparse.php?clone=10817&frame=1&accno=38111045#x) | 2 | 1 |
|  | Coatomer gamma-2 subunit | [*Aspergillus nidulans*/XP_408684](http://200.137.194.69/phorestwww/ncbi.php?accno=40741700) | [4e -60](http://200.137.194.69/phorestwww/xmlparse.php?clone=10570&frame=3&accno=40741700#x) | 1 | 1 |
|  | Clathrin adaptor appendage domain protein^b^ | [*Aspergillus nidulans*/XP_408344](http://200.137.194.69/phorestwww/ncbi.php?accno=40740116) | [1e -13](http://200.137.194.69/phorestwww/xmlparse.php?clone=10880&frame=3&accno=40740116#x) | - | 1 |
|  | ER to Golgi transport related protein^b^ | [*Aspergillus nidulans*/XP_412331](http://200.137.194.69/phorestwww/ncbi.php?accno=40739648) | [5e -75](http://200.137.194.69/phorestwww/xmlparse.php?clone=10527&frame=2&accno=40739648#x) | - | 2 |
|  | Vacuolar protein sorting/targeting protein PEP1 precursor^b^ | *Coccidioides immitis*/EAS36959 | 1e -42 | - | 1 |
|  | Vacuolar protein sorting 29 | *Aspergillus nidulans*/ XP_658945 | 2e -63 | 11 | 1 |
|  | Regulator of V-ATPase in vacuolar membrane protein* | [*Aspergillus nidulans*/XP_404840](http://200.137.194.69/phorestwww/ncbi.php?accno=40746323) | [9e -59](http://200.137.194.69/phorestwww/xmlparse.php?clone=10272&frame=3&accno=40746323#x) | - | 1 |
|  | LMBR1 integral membrane protein-like | [*Aspergillus nidulans*/XP_408348](http://200.137.194.69/phorestwww/ncbi.php?accno=40740120) | [9e -14](http://200.137.194.69/phorestwww/xmlparse.php?clone=10152&frame=1&accno=40740120#x) | 2 | 1 |
|  | ADP-ribosylation factor 2 | [*Ajellomyces capsulata*/P34727](http://200.137.194.69/phorestwww/ncbi.php?accno=1362500) | [2e -63](http://200.137.194.69/phorestwww/xmlparse.php?clone=10886&frame=3&accno=1362500#x) | 2 | 2 |
|  | T-snare superfamily protein | *Aspergillus nidulans*/XP_411817 | [1e -23](http://200.137.194.69/phorestwww/xmlparse.php?clone=19476&frame=2&accno=40742676#x) | 3 | 1 |
|  | Tctex-1 family protein* | [*Aspergillus nidulans*/XP_405470](http://200.137.194.69/phorestwww/ncbi.php?accno=40746360) | [6e -25](http://200.137.194.69/phorestwww/xmlparse.php?clone=10333&frame=2&accno=40746360#x) | - | 2 |
|  | Phosphatidylinositol transfer protein^b^ | [*Aspergillus nidulan*s/XP_410990](http://200.137.194.69/phorestwww/ncbi.php?accno=40739062) | [9e -79](http://200.137.194.69/phorestwww/xmlparse.php?clone=10304&frame=2&accno=40739062#x) | - | 1 |
|  | Importing beta protein^b^ | [*Aspergillus nidulans*/XP_410871](http://200.137.194.69/phorestwww/ncbi.php?accno=40739362) | [6e -71](http://200.137.194.69/phorestwww/xmlparse.php?clone=10298&frame=1&accno=40739362#x) | - | 1 |
|  | Importin-beta N-terminal domain protein* | [*Aspergillus nidulans*/XP_410143](http://200.137.194.69/phorestwww/ncbi.php?accno=40738565) | [1e -44](http://200.137.194.69/phorestwww/xmlparse.php?clone=10710&frame=1&accno=40738565#x) | - | 1 |
|  | Phox homology (PX) domain protein^b^ | [*Aspergillus nidulans*/XP_410488](http://200.137.194.69/phorestwww/ncbi.php?accno=40739545) | [3e -06](http://200.137.194.69/phorestwww/xmlparse.php?clone=10536&frame=3&accno=40739545#x) | - | 1 |
|  | Lysine-specific permease | *Coccidioides immitis*/ EAS34877 | 4e-46 | 12 | 1 |
|  | Nucleoporin SONB^a^ | *Aspergillus fumigatus*/ XP_751721 | 7e -47 | 1 | 2 |
|  | GTP-binding protein ypt1 | *Phaeosphaeria nodorum* **/**EAT86676 | 3e -63 | 47 | 1 |
|  | Exocyst complex component Sec15 protein^a^ | *Coccidioides immitis* **/**EAS37215 | 4e -65 | 1 | 3 |
|  |  |  |  |  |  |
| **Signal Transduction** | [Two-component sensor kinase*](http://200.137.194.69/phorestwww/ncbi.php?accno=50121360) | *Anopheles gambiae*/EAA02130 | 2e -38 | - | 6 |
| RACK1-like protein | *Aspergillus nidulans*/EAA59424 | [9e -94](http://200.137.194.69/phorestwww/xmlparse.php?clone=10878&frame=2&accno=40740234#x) | 1 | 1 |
|  | Rhodopsin-like GPCR superfamily protein | [*Aspergillus nidulans*/XP_409821](http://200.137.194.69/phorestwww/ncbi.php?accno=40743587) | [4e -23](http://200.137.194.69/phorestwww/xmlparse.php?clone=10894&frame=2&accno=40743587#x) | 1 | 1 |
|  | Histidine protein kinase sensor for GlnG regulator*^#^ | *Tetrahymena thermophila*/[EAR83219](http://www.ncbi.nlm.nih.gov/entrez/query.fcgi?cmd=Retrieve&db=Protein&list_uids=89285199&dopt=GenPept) | 2e -04 | - | 24 |
|  | Protein kinase C conserved region 2^b^ | [*Aspergillus nidulans*/XP_409761](http://200.137.194.69/phorestwww/ncbi.php?accno=40743527) | [3e -55](http://200.137.194.69/phorestwww/xmlparse.php?clone=10329&frame=1&accno=40743527#x) | - | 1 |
|  | Anti-silencing factor 1 | [*Gibberella zeae*/XP_380526](http://200.137.194.69/phorestwww/ncbi.php?accno=42546767) | [1e -64](http://200.137.194.69/phorestwww/xmlparse.php?clone=10434&frame=1&accno=42546767#x) | 4 | 1 |
|  | WD40 domain protein | [*Aspergillus nidulans*/XP_405832](http://200.137.194.69/phorestwww/ncbi.php?accno=40745659) | [2e -25](http://200.137.194.69/phorestwww/xmlparse.php?clone=10503&frame=1&accno=40745659#x) | 1 | 1 |
|  | UVSB Phosphatidylinositol-3 kinase*^#^ | [*Aspergillus nidulans*/XP_411112](http://200.137.194.69/phorestwww/ncbi.php?accno=40742431) | [1e -29](http://200.137.194.69/phorestwww/xmlparse.php?clone=10695&frame=2&accno=40742431#x) | - | 1 |
|  | Serine/threonine-protein kinase SAT4^b^ | [*Aspergillus nidulans* /XP_412967](http://200.137.194.69/phorestwww/ncbi.php?accno=40740928) | [2e -51](http://200.137.194.69/phorestwww/xmlparse.php?clone=10766&frame=2&accno=40740928#x) | - | 1 |
|  | Serine/Threonine protein kinase, catalytic domain | [*Aspergillus nidulans*/XP_411674](http://200.137.194.69/phorestwww/ncbi.php?accno=40742927) | [3e -06](http://200.137.194.69/phorestwww/xmlparse.php?clone=10577&frame=2&accno=40742927#x) | 1 | 1 |
|  | Serine/threonine kinase receptor associated protein | [*Aspergillus nidulans*/XP_411842](http://200.137.194.69/phorestwww/ncbi.php?accno=40742030) | [4e -73](http://200.137.194.69/phorestwww/xmlparse.php?clone=10103&frame=2&accno=40742030#x) | 1 | 1 |
|  | Serine/threonine protein kinase | [*Aspergillus nidulans*/XP_404372](http://200.137.194.69/phorestwww/ncbi.php?accno=40746952) | [2e -49](http://200.137.194.69/phorestwww/xmlparse.php?clone=10073&frame=2&accno=40746952#x) | 2 | 2 |
|  | Protein kinase | [*Magnaporthe grisea*/XP_363079](http://200.137.194.69/phorestwww/ncbi.php?accno=38104597) | [1e -12](http://200.137.194.69/phorestwww/xmlparse.php?clone=10875&frame=2&accno=38104597#x) | 1 | 1 |
|  | Mitogen activated protein kinase^b^ | [*Aspergillus oryzae*](http://200.137.194.69/phorestwww/ncbi.php?accno=45504120)/BAD12561 | [1e -52](http://200.137.194.69/phorestwww/xmlparse.php?clone=10216&frame=3&accno=45504120#x) | - | 1 |
|  | GTPase, G3E family protein^b^ | [*Neurospora crassa*](http://200.137.194.69/phorestwww/ncbi.php?accno=32404938)/XP_323082 | [1e -11](http://200.137.194.69/phorestwww/xmlparse.php?clone=10861&frame=3&accno=32404938#x) | - | 1 |
|  | Rho GTPase activating protein* | [*Aspergillus nidulans*/XP_407883](http://200.137.194.69/phorestwww/ncbi.php?accno=40740764) | [3e -49](http://200.137.194.69/phorestwww/xmlparse.php?clone=10241&frame=3&accno=40740764#x) | - | 1 |
|  | Rho1 GTPase | [*Paracoccidioides brasiliensis*](http://200.137.194.69/phorestwww/ncbi.php?accno=41777352)/AAQ93069 | [7e -82](http://200.137.194.69/phorestwww/xmlparse.php?clone=10033&frame=2&accno=41777352#x) | 4 | 1 |
|  | GTP binding protein^b^ | [*Neurospora crassa*/CAD70888](http://200.137.194.69/phorestwww/ncbi.php?accno=32412930) | [5e -84](http://200.137.194.69/phorestwww/xmlparse.php?clone=10698&frame=2&accno=32412930#x) | - | 1 |
|  | Calcineurin subunit b* | *Neurospora crassa*/P87072 | [1e -77](http://200.137.194.69/phorestwww/xmlparse.php?clone=9976&frame=3&accno=52001480#x) | - | 2 |
|  | Calmodulin | *Fusarium proliferatum*/ AAL04428 | 3e -07 | 5 | 1 |
|  | Forkhead associated (FHA) protein* | [*Gibberella zeae*/XP_389397](http://200.137.194.69/phorestwww/ncbi.php?accno=42554218) | [4e -10](http://200.137.194.69/phorestwww/xmlparse.php?clone=10072&frame=2&accno=42554218#x) | - | 1 |
|  | Ca^2+^- binding protein (EF-Hand superfamily) | *Aspergillus nidulans*/XP_404404 | [2e -36](http://200.137.194.69/phorestwww/xmlparse.php?clone=16620&frame=2&accno=40746984#x) | 1 | 1 |
|  | 14-3-3-like protein 2 | *Paracoccidioides brasiliensis*/ AAR24348 | 5e -26 | 4 | 1 |
|  |  |  |  |  |  |
| **Cell Rescue, Defense and Virulence** | Potential secreted Cu/Zn superoxide dismutase^b^ | [*Magnaporthe grisea*/XP_360807](http://200.137.194.69/phorestwww/ncbi.php?accno=38105314) | [8e -11](http://200.137.194.69/phorestwww/xmlparse.php?clone=9973&frame=3&accno=38105314#x) | - | 1 |
| Thiol specific antioxidant protein | *Ajellomyces capsulatus*/ AAG31645 | 1e -71 | 2 | 1 |
| Peroxisomal like protein | *Paracoccidioides brasiliensis*/ AAQ84041 | 2e -33 | 2 | 1 |
|  | Peroxisomal catalase^a^ | [*Paracoccidioides brasiliensis*/AAL34518](http://200.137.194.69/phorestwww/ncbi.php?accno=30995492) | [4e -66](http://200.137.194.69/phorestwww/xmlparse.php?clone=10382&frame=1&accno=30995492#x) | 3 | 4 |
|  | Heat shock protein 10, mitochondrial^a^ | [*Gibberella zeae*/XP_386383](http://200.137.194.69/phorestwww/ncbi.php?accno=42551720) | [2e -40](http://200.137.194.69/phorestwww/xmlparse.php?clone=10859&frame=1&accno=42551720#x) | 1 | 3 |
|  | Heat shock protein 30 | *Aspergillus nidulans*/XP_406667 | [3e -45](http://200.137.194.69/phorestwww/xmlparse.php?clone=17649&frame=1&accno=40745479#x) | 2 | 1 |
|  | Heat shock protein 60, mitochondrial precursor | *Paracoccidioides brasiliensis*/AAC14712 | 2e -39 | 1 | 1 |
|  | Heat shock protein 60^b^ | *Aspergillus nidulans* **/**XP_659508 | 3e -12 | - | 1 |
|  | Heat shock protein 70^b^ | [*Paracoccidioides brasiliensis*](http://200.137.194.69/phorestwww/ncbi.php?accno=31324921)/AAP05987 | [3e -64](http://200.137.194.69/phorestwww/xmlparse.php?clone=10139&frame=1&accno=31324921#x) | - | 2 |
|  | Heat shock protein 70 | [*Paracoccidioides brasiliensis*](http://200.137.194.69/phorestwww/ncbi.php?accno=14538021)/AAK66771 | [4e -81](http://200.137.194.69/phorestwww/xmlparse.php?clone=9972&frame=1&accno=14538021#x) | 31 | 7 |
|  | Heat shock protein 70^b^ | [*Emericella nidulans*](http://200.137.194.69/phorestwww/ncbi.php?accno=2764949)/CAA67431 | [7e -39](http://200.137.194.69/phorestwww/xmlparse.php?clone=10548&frame=2&accno=2764949#x) | - | 1 |
|  | Heat shock protein 82 | *Aspergillus nidulans*/EAA59007 | [1e -12](http://200.137.194.69/phorestwww/xmlparse.php?clone=10627&frame=2&accno=40739817#x) | 8 | 1 |
|  | Heat shock protein 88 | [*Aspergillus nidulans*/XP_405184](http://200.137.194.69/phorestwww/ncbi.php?accno=40747009) | [1e -80](http://200.137.194.69/phorestwww/xmlparse.php?clone=10178&frame=1&accno=40747009#x) | 9 | 3 |
|  | Heat shock protein 90^a^ | *Aspergillus nidulans/*EAA59007 | [4e -38](http://200.137.194.69/phorestwww/xmlparse.php?clone=17239&frame=1&accno=40739817#x) | 5 | 6 |
|  | MDJ1-like protein | [*Aspergillus nidulans*/XP_410331](http://200.137.194.69/phorestwww/ncbi.php?accno=40738790) | [2e -16](http://200.137.194.69/phorestwww/xmlparse.php?clone=10320&frame=3&accno=40738790#x) | 4 | 1 |
|  | DNAJ protein | *Coccidioides immitis* **/** EAS35517 | 7e -77 | 14 | 3 |
|  | Hemolysin | [*Magnaporthe grisea*/XP_359943](http://200.137.194.69/phorestwww/ncbi.php?accno=38099942) | [2e -11](http://200.137.194.69/phorestwww/xmlparse.php?clone=10830&frame=1&accno=38099942#x) | 3 | 2 |
|  | Hemolysin like protein*^#^ | [*Aspergillus nidulans*/XP_406013](http://200.137.194.69/phorestwww/ncbi.php?accno=40745885) | [2e -70](http://200.137.194.69/phorestwww/xmlparse.php?clone=10650&frame=2&accno=40745885#x) | - | 1 |
|  | Copper-zinc superoxide dismutase | *Paracoccidioides brasiliensis*/AAX13803 | 2e -24 | 11 | 1 |
|  | Hemerythrin HHE cation binding domain protein | [*Magnaporthe grisea*/XP_360008](http://200.137.194.69/phorestwww/ncbi.php?accno=38108596) | [1e -05](http://200.137.194.69/phorestwww/xmlparse.php?clone=10534&frame=3&accno=38108596#x) | 1 | 1 |
|  | Fasciclin-like protein | [*Aspergillus nidulans*/XP_405664](http://200.137.194.69/phorestwww/ncbi.php?accno=40744627) | [3e -19](http://200.137.194.69/phorestwww/xmlparse.php?clone=10526&frame=3&accno=40744627#x) | 3 | 1 |
|  |  |  |  |  |  |
| **Biogenesis of cellular components** | Tubulin alpha 1^b^ | [*Aspergillus nidulans*/XP_411707](http://200.137.194.69/phorestwww/ncbi.php?accno=40742960) | [1e -63](http://200.137.194.69/phorestwww/xmlparse.php?clone=10826&frame=1&accno=40742960#x) | - | 1 |
| Tubulin beta-1 chain^b^ | *Aspergillus flavus*/P22012 | [7e -120](http://200.137.194.69/phorestwww/xmlparse.php?clone=16498&frame=1&accno=280475#x) | - | 1 |
|  | Actin related protein 3 | [*Gibberella zeae*/XP_391032](http://200.137.194.69/phorestwww/ncbi.php?accno=42551466) | [9e -48](http://200.137.194.69/phorestwww/xmlparse.php?clone=10848&frame=3&accno=42551466#x) | 2 | 1 |
|  | Actin related protein 2/3 complex, subunit 1A | [*Coccidioides immitis*](http://200.137.194.69/phorestwww/ncbi.php?accno=15811426)/AAL08969 | [1e -18](http://200.137.194.69/phorestwww/xmlparse.php?clone=10480&frame=2&accno=15811426#x) | 30 | 1 |
|  | Actin related protein^b^ | [*Aspergillus nidulans*/XP_411146](http://200.137.194.69/phorestwww/ncbi.php?accno=40742465) | [1e -40](http://200.137.194.69/phorestwww/xmlparse.php?clone=10516&frame=1&accno=40742465#x) | - | 1 |
|  | Histone H3^a^ | *Aspergillus fumigatus*/XP_752749 | [5e -67](http://200.137.194.69/phorestwww/xmlparse.php?clone=10455&frame=3&accno=21322637#x) | 11 | 6 |
|  | Histone H4 | [*Aspergillus nidulans*/XP_404254](http://200.137.194.69/phorestwww/ncbi.php?accno=40746139) | [7e -44](http://200.137.194.69/phorestwww/xmlparse.php?clone=10225&frame=3&accno=40746139#x) | 24 | 6 |
|  | Histone H2A^a^ | [*Aspergillus nidulans*/XP_412176](http://200.137.194.69/phorestwww/ncbi.php?accno=40740471) | [2e -52](http://200.137.194.69/phorestwww/xmlparse.php?clone=10120&frame=2&accno=40740471#x) | 7 | 6 |
|  | Histone H2B | *Coccidioides immitis*/EAS36779 | [4e -28](http://200.137.194.69/phorestwww/xmlparse.php?clone=10488&frame=1&accno=27531291#x) | 12 | 2 |
|  | Peroxisomal import complex protein Pex12 | [*Aspergillus nidulans*/XP_411443](http://200.137.194.69/phorestwww/ncbi.php?accno=40742167) | [5e -26](http://200.137.194.69/phorestwww/xmlparse.php?clone=10442&frame=2&accno=40742167#x) | 2 | 1 |
|  |  |  |  |  |  |
| **Cell type differentiation** |  |  |  |  |  |
| Suppressor of anucleate metulae B protein*^#^ | [*Aspergillus nidulans*/XP_404215.1](http://200.137.194.69/phorestwww/ncbi.php?accno=40746100) | [6e -46](http://200.137.194.69/phorestwww/xmlparse.php?clone=10487&frame=3&accno=40746100#x) | - | 2 |
|  |  |  |  |  |  |
| **Unclassified** | Y20 protein | [*Paracoccidioides brasiliensis*](http://200.137.194.69/phorestwww/ncbi.php?accno=17980998)/AAL50803 | [4e -63](http://200.137.194.69/phorestwww/xmlparse.php?clone=10190&frame=1&accno=17980998#x) | 4 | 1 |
|  | Predicted membrane protein | [*Rattus norvegicus*](http://200.137.194.69/phorestwww/ncbi.php?accno=34862932)/XP_345034 | [6e -05](http://200.137.194.69/phorestwww/xmlparse.php?clone=10510&frame=-2&accno=34862932#x) | 42 | 3 |
|  | Complex 1 protein (LYR family)* | [*Aspergillus nidulans*/XP_408902](http://200.137.194.69/phorestwww/ncbi.php?accno=40741617) | [8e -32](http://200.137.194.69/phorestwww/xmlparse.php?clone=10681&frame=1&accno=40741617#x) | - | 1 |
|  | Homolog of translationally controlled tumor protein^a^ | [*Aspergillus nidulans*/XP_404778](http://200.137.194.69/phorestwww/ncbi.php?accno=40746028) | [4e -25](http://200.137.194.69/phorestwww/xmlparse.php?clone=10056&frame=3&accno=40746028#x) | 3 | 3 |
|  | YCII related domain^b^ | [*Gibberella zeae*/XP_390542](http://200.137.194.69/phorestwww/ncbi.php?accno=42546425) | [2e -23](http://200.137.194.69/phorestwww/xmlparse.php?clone=10633&frame=2&accno=42546425#x) | - | 1 |
|  | 27 kDa antigen | [*Paracoccidioides brasiliensis*](http://200.137.194.69/phorestwww/ncbi.php?accno=1778408)/AAC49615 | [2e -67](http://200.137.194.69/phorestwww/xmlparse.php?clone=10248&frame=1&accno=1778408#x) | 1 | 1 |
|  | Dimeric alpha-beta barrel domain^b^ | [*Aspergillus nidulans*/XP_406116](http://200.137.194.69/phorestwww/ncbi.php?accno=40744724) | [1e -23](http://200.137.194.69/phorestwww/xmlparse.php?clone=10891&frame=2&accno=40744724#x) | - | 1 |
|  | Isopenicillin N synthase and related dioxygenase | [*Magnaporthe grisea*/XP_364516](http://200.137.194.69/phorestwww/ncbi.php?accno=38104893) | [7e -05](http://200.137.194.69/phorestwww/xmlparse.php?clone=10375&frame=-1&accno=38104893#x) | 2 | 1 |
|  | Iron-sulfur cluster Isu1-like protein^a^ | [*Gibberella zeae*/XP_382800.1](http://200.137.194.69/phorestwww/ncbi.php?accno=42547060) | [3e -41](http://200.137.194.69/phorestwww/xmlparse.php?clone=10663&frame=3&accno=42547060#x) | 5 | 4 |

^a^ Transcripts induced in the transition library in comparison to the mycelium transcriptome according to the Audic and Claverie´s method.

^b^ Transcripts non detected in the mycelia transcriptome (https//dna.biomol.unb.br/Pb).

* Novel genes detected in *P. brasiliensis*.

^#^ Transcripts confirmed by semi-quantitative RT-PCR.

^+^Genes not described previously in *P. brasiliensis* isolate *Pb*01, but present in public databases.
