# Supplementary material for: The transcriptome analysis of early morphogenesis in Paracoccidioides brasiliensis mycelium reveals novel and induced genes potentially associated to the dimorphic process
Source: BMC Microbiol. 2007 Apr 10;7:29. doi: 10.1186/1471-2180-7-29 (PMC1855332; doi:10.1186/1471-2180-7-29)
Supplement: Additional File 2 — Induced P. brasiliensis ESTs and novel genes generated from the transition library. Table representing the annotated clusters that were generated by sequencing of the cDNA clones. For each cluster the table includes: the function as assigned by BLAST-based similarity, the BLAST subject species, the GenBank ID for the BLAST subject used for functional assignment and the Expect value obtained with each unisequence, the redundancy in the transition library and in the mycelium transcriptome database. [file 1471-2180-7-29-S2.doc]

**Table 2 – Induced *P. brasiliensis* ESTs and novel genes generated from the transition library‡**.

| **MIPS Category** | **Gene Product** | **Best hit organism/Accession number** | **e-value** | **Redundancy** | |
| --- | --- | --- | --- | --- | --- |
| **M** | **T** |
| **Amino acid metabolism** | Histidinol phosphate aminotransferase^b^ | [*Aspergillus fumigatus*](http://200.137.194.69/phorestwww/ncbi.php?accno=44890004)/CAF32122 | [1e -69](http://200.137.194.69/phorestwww/xmlparse.php?clone=10803&frame=2&accno=44890004#x) | - | 1 |
| Diphthine synthase*^#^ | [*Aspergillus fumigatus*](http://200.137.194.69/phorestwww/ncbi.php?accno=44889994)/CAF32112 | [1e -38](http://200.137.194.69/phorestwww/xmlparse.php?clone=10808&frame=2&accno=44889994#x) | - | 2 |
|  | Acetylornithine deacetylase* | *Arabidopsis thaliana*/BP845946 | 1e-31 | - | 1 |
|  | Gamma-glutamyl phosphate reductase^+^ | *Coccidioides immitis*/ EAS33218 | 1e -21 | - | 1 |
|  | Fumarylacetoacetate hydrolase^b^ | *Emericella nidulans*/AAA85778 | [1e -69](http://200.137.194.69/phorestwww/xmlparse.php?clone=10452&frame=1&accno=1130507#x) | - | 2 |
|  | 2,4-dihydroxyhept-2-ene-1,7-dioic acid aldolase^b^ | [*Aspergillus nidulans* /XP_407838](http://200.137.194.69/phorestwww/ncbi.php?accno=40740719) | [5e -07](http://200.137.194.69/phorestwww/xmlparse.php?clone=10562&frame=3&accno=40740719#x) | - | 1 |
|  | Anthranilate phosphoribosyltransferase^a^ | [*Aspergillus nidulans* /XP_407771](http://200.137.194.69/phorestwww/ncbi.php?accno=40740652) | [2e -44](http://200.137.194.69/phorestwww/xmlparse.php?clone=10273&frame=-2&accno=40740652#x) | 1 | 2 |
|  | Histidine ammonia lyase* | *Dictyostelium discoideum*/XP_636944 | 1e -16 | - | 1 |
|  | **Glutamate dehydrogenase (NADP(+))*** | *Emericella nidulans*/ S04904 | 5e -06 | - | 2 |
|  |  |  |  |  |  |
| **Nitrogen and sulfur metabolism** | Nitrogen regulatory protein P-II^b^ | [*Aspergillus nidulans*/XP_408436](http://200.137.194.69/phorestwww/ncbi.php?accno=40740849) | [5e -37](http://200.137.194.69/phorestwww/xmlparse.php?clone=10006&frame=2&accno=40740849#x) | - | 2 |
| Acetamidase^b^ | [*Aspergillus nidulans*/XP_405649](http://200.137.194.69/phorestwww/ncbi.php?accno=40744669) | [2e -53](http://200.137.194.69/phorestwww/xmlparse.php?clone=9974&frame=2&accno=40744669#x) | - | 1 |
| Sulfite reductase NADPH beta subunit^b^ | [*Aspergillus nidulans*/XP_411737](http://200.137.194.69/phorestwww/ncbi.php?accno=40742990) | [3e -75](http://200.137.194.69/phorestwww/xmlparse.php?clone=10045&frame=2&accno=40742990#x) | - | 1 |
|  | Urease, alpha subunit^a^ | [*Aspergillus fumigatus*](http://200.137.194.69/phorestwww/ncbi.php?accno=50788080)/CAE17672 | [1e -71](http://200.137.194.69/phorestwww/xmlparse.php?clone=10685&frame=3&accno=50788080#x) | 1 | 4 |
|  | Cyanate lyase^b^ | [*Aspergillus nidulans*/XP_411468](http://200.137.194.69/phorestwww/ncbi.php?accno=40742192) | [6e -46](http://200.137.194.69/phorestwww/xmlparse.php?clone=10622&frame=3&accno=40742192#x) | - | 1 |
|  | Thiosulfate sulphurtransferase^a^ | [*Gibberella zeae*/XP_381684](http://200.137.194.69/phorestwww/ncbi.php?accno=42546050) | [1e -45](http://200.137.194.69/phorestwww/xmlparse.php?clone=10605&frame=2&accno=42546050#x) | 1 | 2 |
|  |  |  |  |  |  |
| **Nucleotide metabolism** | Nudix hydrolase family protein* | [*Aspergillus nidulans*/XP_409279](http://200.137.194.69/phorestwww/ncbi.php?accno=40743133) | [1e -19](http://200.137.194.69/phorestwww/xmlparse.php?clone=10834&frame=2&accno=40743133#x) | - | 3 |
| RNA (guanine-N7-) methyltransferase^a^ | [*Neurospora crassa*](http://200.137.194.69/phorestwww/ncbi.php?accno=40882314)/CAF06136 | [2e -18](http://200.137.194.69/phorestwww/xmlparse.php?clone=10483&frame=3&accno=40882314#x) | 1 | 2 |
|  | Adenosine deaminase* | *Aspergillus oryzae***/**BAE60718 | 2e -34 | - | 4 |
|  | Orotate phosphoribosyltransferase* | [*Mortierella alpina*](http://www.ncbi.nlm.nih.gov/Taxonomy/Browser/wwwtax.cgi?id=64518)/BAD29963 | 3e -44 | - | 2 |
|  | Pyrimidine 5-nucleotidase^+^ | [*Aspergillus nidulans*/XP_410462](http://200.137.194.69/phorestwww/ncbi.php?accno=40739519) | [4e -49](http://200.137.194.69/phorestwww/xmlparse.php?clone=10435&frame=3&accno=40739519#x) | - | 2 |
|  | TatD DNAse^b^ | [*Aspergillus nidulans*/XP_408185](http://200.137.194.69/phorestwww/ncbi.php?accno=40740329) | [4e -10](http://200.137.194.69/phorestwww/xmlparse.php?clone=10794&frame=1&accno=40740329#x) | - | 1 |
|  | Xanthine dehydrogenase* | *Gibberella zeae*/XP_381737 | 9e -07 | - | 1 |
|  |  |  |  |  |  |
|  |  |  |  |  |  |
| **Phosphate metabolism** | phnO protein* | *Rhizopus oryzae*/EE002192 | 4e -116 | - | 4 |
|  | Inorganic pyrophosphatase^b^ | *Coccidioides immitis*/ EAS28880 | 3e -32 | - | 1 |
|  |  |  |  |  |  |
| **C-compound and carbohydrate metabolism** | Alpha-1,3-glucan synthase^b^ | [*Paracoccidioides brasiliensis*/](http://200.137.194.69/phorestwww/ncbi.php?accno=55509190)AAV52833 | [8e -68](http://200.137.194.69/phorestwww/xmlparse.php?clone=9964&frame=1&accno=55509190#x) | - | 1 |
| Glucanosyltransferase family protein^a^ | *Aspergillus nidulans*/XP_408051 | [9e -35](http://200.137.194.69/phorestwww/xmlparse.php?clone=9708&frame=2&accno=40740033#x) | 1 | 3 |
| Chitinase 1^a^ | [*Aspergillus nidulans*/XP_413527](http://200.137.194.69/phorestwww/ncbi.php?accno=40747301) | [1e -1](http://200.137.194.69/phorestwww/xmlparse.php?clone=10380&frame=1&accno=40747301#x)8 | 1 | 2 |
|  | Chitinase 3*^#^ | [*Coccidioides immitis*/](http://200.137.194.69/phorestwww/ncbi.php?accno=29539602)AAO88269 | [7e -40](http://200.137.194.69/phorestwww/xmlparse.php?clone=10213&frame=3&accno=29539602#x) | - | 1 |
|  | Mannitol-1-phosphate dehydrogenase^a^ | [*Paracoccidioides brasiliensis*/](http://200.137.194.69/phorestwww/ncbi.php?accno=28797565)AAO47089 | [2e -88](http://200.137.194.69/phorestwww/xmlparse.php?clone=10439&frame=3&accno=28797565#x) | 2 | 3 |
|  | Uridine diphosphate glucose pyrophosphorylase^a^ | [*Emericella nidulans*/](http://200.137.194.69/phorestwww/ncbi.php?accno=57236787)AAW49005 | [3e -69](http://200.137.194.69/phorestwww/xmlparse.php?clone=10002&frame=3&accno=57236787#x) | 1 | 2 |
|  | Alpha-glucosidase I*^#^ | [*Aspergillus fumigatus*](http://200.137.194.69/phorestwww/ncbi.php?accno=38564743)/AAR23808 | [3e -46](http://200.137.194.69/phorestwww/xmlparse.php?clone=10632&frame=3&accno=38564743#x) | - | 2 |
|  |  |  |  |  |  |
| **Lipid metabolism** | Myo-inositol-1-phosphate synthase^+^ | [*Aspergillus nidulans*/XP_411762](http://200.137.194.69/phorestwww/ncbi.php?accno=40742621) | [4e -15](http://200.137.194.69/phorestwww/xmlparse.php?clone=10344&frame=1&accno=40742621#x) | - | 1 |
| Glycerophosphodiester phosphodiesterase^a^ | [*Aspergillus nidulans*/XP_404274](http://200.137.194.69/phorestwww/ncbi.php?accno=40746159) | [9e -70](http://200.137.194.69/phorestwww/xmlparse.php?clone=10540&frame=3&accno=40746159#x) | 1 | 4 |
|  | Esterase family protein^+^ | [*Aspergillus nidulans* /XP_412451](http://200.137.194.69/phorestwww/ncbi.php?accno=40747781) | [2e -13](http://200.137.194.69/phorestwww/xmlparse.php?clone=10814&frame=3&accno=40747781#x) | - | 1 |
|  | Lysophospholipase^b^ | [*Aspergillus nidulans*/XP_412885](http://200.137.194.69/phorestwww/ncbi.php?accno=40741351) | [3e -58](http://200.137.194.69/phorestwww/xmlparse.php?clone=10606&frame=2&accno=40741351#x) | - | 1 |
|  | Phospholipase A2^+^ | *Coccidioides immitis*/ EAS34384 | 3e-27 | - | 1 |
|  | Peroxisomal hydratase dehydrogenase epimerase^b^ | [*Aspergillus nidulans*/XP_411248](http://200.137.194.69/phorestwww/ncbi.php?accno=40742126) | [1e -34](http://200.137.194.69/phorestwww/xmlparse.php?clone=10760&frame=2&accno=40742126#x) | - | 4 |
|  | Serine esterase^+^ | [*Aspergillus nidulans*/XP_406618](http://200.137.194.69/phorestwww/ncbi.php?accno=40744643) | [8e -92](http://200.137.194.69/phorestwww/xmlparse.php?clone=10364&frame=1&accno=40744643#x) | - | 3 |
|  | Glycerol-3-phosphate dehydrogenase (NAD(P)+)* | *Cryptococcus neoformans*/AAM26266 | 2e -14 | - | 1 |
|  | Phosphatidylserine synthase*^#^ | [*Neurospora crassa*/EAA30566](http://200.137.194.69/phorestwww/ncbi.php?accno=32421427) | [6e -38](http://200.137.194.69/phorestwww/xmlparse.php?clone=10504&frame=3&accno=32421427#x) | - | 1 |
|  | Fatty acid desaturase^a^ | [*Neurospora crassa*/EAA29146](http://200.137.194.69/phorestwww/ncbi.php?accno=32420673) | [4e -48](http://200.137.194.69/phorestwww/xmlparse.php?clone=10873&frame=3&accno=32420673#x) | 1 | 2 |
|  | Sterol delta 5,6-desaturase ERG3^b^ | [*Aspergillus nidulans*/XP_410643](http://200.137.194.69/phorestwww/ncbi.php?accno=40738656) | [7e -40](http://200.137.194.69/phorestwww/xmlparse.php?clone=10055&frame=2&accno=40738656#x) | - | 1 |
|  | Lanosterol 14-alpha-demethylase^a^ | [*Ajellomyces capsulatus*](http://200.137.194.69/phorestwww/ncbi.php?accno=51341096)/AAU01158 | [1e -89](http://200.137.194.69/phorestwww/xmlparse.php?clone=9956&frame=3&accno=51341096#x) | 3 | 4 |
|  | Lipoic acid synthase^b^ | [*Aspergillus nidulans*/XP_413623](http://200.137.194.69/phorestwww/ncbi.php?accno=40747621) | [8e -40](http://200.137.194.69/phorestwww/xmlparse.php?clone=10131&frame=3&accno=40747621#x) | - | 1 |
|  |  |  |  |  |  |
| **Metabolism of vitamins, cofactors and prosthetic groups** | Uroporphyrinogen III methylase* | [*Rhizopus oryzae*](http://www.ncbi.nlm.nih.gov/Taxonomy/Browser/wwwtax.cgi?name=Rhizopus+oryzae)/EE010378 | 6e-109 | - | 4 |
| Ubiquinone/menaquinone biosynthesis methyltransferase UbiE^b^ | *Dictyostelium discoideum*/XP_641323 | 2e-12 | - | 18 |
| Para aminobenzoic acid synthetase^b^ | [*Aspergillus nidulans*/XP_410687](http://200.137.194.69/phorestwww/ncbi.php?accno=40738700) | [8e -19](http://200.137.194.69/phorestwww/xmlparse.php?clone=10879&frame=2&accno=40738700#x) | - | 1 |
|  |  |  |  |  |
|  |  |  |  |  |  |
| **Energy** | Phosphoglucomutase^b^ | [*Neurospora crassa*/EAA34468](http://200.137.194.69/phorestwww/ncbi.php?accno=32405624) | [5e -73](http://200.137.194.69/phorestwww/xmlparse.php?clone=10074&frame=3&accno=32405624#x) | - | 1 |
|  | Glyceraldehyde-3-phosphate dehydrogenase^b^ | [*Paracoccidioides brasiliensis*](http://200.137.194.69/phorestwww/ncbi.php?accno=30995493)/AAL34975 | [7e -70](http://200.137.194.69/phorestwww/xmlparse.php?clone=14021&frame=2&accno=30995493#x) | - | 1 |
|  | NADH dehydrogenase, 21 kDa subunit^b^ | [*Aspergillus nidulans*/XP_411113](http://200.137.194.69/phorestwww/ncbi.php?accno=40742432) | [4e -38](http://200.137.194.69/phorestwww/xmlparse.php?clone=9999&frame=1&accno=40742432#x) | - | 1 |
|  | Ubiquinol cytochrome c reductase^b^ | [*Aspergillus nidulans*/XP_408525](http://200.137.194.69/phorestwww/ncbi.php?accno=40741115) | [6e -39](http://200.137.194.69/phorestwww/xmlparse.php?clone=9993&frame=3&accno=40741115#x) | - | 2 |
|  | Ferredoxin-like iron-sulfur protein^a^ | [*Paracoccidioides brasiliensis*](http://200.137.194.69/phorestwww/ncbi.php?accno=30351132)/AAP23044 | [2e -58](http://200.137.194.69/phorestwww/xmlparse.php?clone=10307&frame=1&accno=30351132#x) | 2 | 3 |
|  | Protein PET191, mitochondrial precursor^b^ | [*Gibberella zeae*/XP_388901](http://200.137.194.69/phorestwww/ncbi.php?accno=42549579) | [4e -11](http://200.137.194.69/phorestwww/xmlparse.php?clone=10862&frame=1&accno=42549579#x) | - | 1 |
|  | ATP synthase gamma chain^a^ | [*Aspergillus nidulans*/XP_404389](http://200.137.194.69/phorestwww/ncbi.php?accno=40746969) | [9e -57](http://200.137.194.69/phorestwww/xmlparse.php?clone=10505&frame=1&accno=40746969#x) | 1 | 3 |
|  | ATP synthase subunit 9^a^ | *Neurospora crassa*/T43671 | [8e -22](http://200.137.194.69/phorestwww/xmlparse.php?clone=10115&frame=3&accno=11266857#x) | 1 | 4 |
|  | Acyl-coenzyme A synthetase^b^ | [*Neurospora crassa*/EAA26946](http://200.137.194.69/phorestwww/ncbi.php?accno=32403204) | [5e -10](http://200.137.194.69/phorestwww/xmlparse.php?clone=10580&frame=2&accno=32403204#x) | - | 1 |
|  | L-carnitine dehydratase^+^ | [*Aspergillus nidulans*/XP_408367](http://200.137.194.69/phorestwww/ncbi.php?accno=40740139) | [1e -62](http://200.137.194.69/phorestwww/xmlparse.php?clone=10090&frame=3&accno=40740139#x) | - | 1 |
|  | Acetyl CoA hydrolase* | [*Aspergillus nidulans*/XP_405684](http://200.137.194.69/phorestwww/ncbi.php?accno=40745098) | [5e -42](http://200.137.194.69/phorestwww/xmlparse.php?clone=10067&frame=3&accno=40745098#x) | - | 1 |
|  |  |  |  |  |  |
| **Cell cycle and DNA processing** | Endonuclease III^b^ | [*Aspergillus nidulans*/XP_411790](http://200.137.194.69/phorestwww/ncbi.php?accno=40742649) | [1e -06](http://200.137.194.69/phorestwww/xmlparse.php?clone=11228&frame=1&accno=40742649#x) | - | 1 |
| Rad21 protein* | [*Neurospora crassa*/EAA34981](http://200.137.194.69/phorestwww/ncbi.php?accno=32420567) | [6e -17](http://200.137.194.69/phorestwww/xmlparse.php?clone=10884&frame=2&accno=32420567#x) | - | 2 |
| Prohibitin 2^a^ | [*Aspergillus nidulans*/XP_410210](http://200.137.194.69/phorestwww/ncbi.php?accno=40738858) | [6e -52](http://200.137.194.69/phorestwww/xmlparse.php?clone=10150&frame=1&accno=40738858#x) | 3 | 5 |
|  | Arrestin, N-terminal domain^b^ | [*Aspergillus nidulans*/XP_404193](http://200.137.194.69/phorestwww/ncbi.php?accno=40746078) | [2e -56](http://200.137.194.69/phorestwww/xmlparse.php?clone=10905&frame=1&accno=40746078#x) | - | 1 |
|  | Proliferating Cell Nuclear Antigen (PCNA)* | [*Aspergillus nidulans*/XP_404552](http://200.137.194.69/phorestwww/ncbi.php?accno=40747358) | [3e -36](http://200.137.194.69/phorestwww/xmlparse.php?clone=10782&frame=2&accno=40747358#x) | - | 1 |
|  | DNA polymerase delta 2 subunit^+^ | *Coccidioides immitis*/EAS28821 | 6e -33 | - | 2 |
|  | DNA polymerase eta^b^ | *Aspergillus nidulans*/XP_408815 | [1e -32](http://200.137.194.69/phorestwww/xmlparse.php?clone=18855&frame=3&accno=40741530#x) | - | 1 |
|  | Heterokaryon incompatibility protein Het-C^b^ | [*Aspergillus nidulans*/XP_406304](http://200.137.194.69/phorestwww/ncbi.php?accno=40745055) | [1e -27](http://200.137.194.69/phorestwww/xmlparse.php?clone=10413&frame=1&accno=40745055#x) | - | 2 |
|  | Uracil DNA glycosylase* | *Aspergillus fumigatus*/ XP_749743 | 3e -24 | - | 1 |
|  | Cell division protein 48 (CDC48)^a^ | *Coccidioides immitis* ***/***EAS28203 | 9e -15 | 2 | 3 |
|  | Chromosome segregation ATPase* | *Coccidioides immitis* **/**EAS30662 | 6e -52 | - | 1 |
|  |  |  |  |  |  |
| **Transcription** | DEAD-like helicases superfamily protein* ^#^ | [*Aspergillus nidulans*/XP_410144](http://200.137.194.69/phorestwww/ncbi.php?accno=40738566) | 3[e -55](http://200.137.194.69/phorestwww/xmlparse.php?clone=10424&frame=3&accno=40738566#x) | - | 1 |
|  | Transcription factor, bromodomain* | [*Aspergillus nidulans*/EAA60972](http://200.137.194.69/phorestwww/ncbi.php?accno=40741782) | [2e -55](http://200.137.194.69/phorestwww/xmlparse.php?clone=10495&frame=2&accno=40741782#x) | - | 1 |
|  | GatB/YqeY domain protein* | [*Aspergillus nidulans*/XP_410874](http://200.137.194.69/phorestwww/ncbi.php?accno=40739365) | [1e -22](http://200.137.194.69/phorestwww/xmlparse.php?clone=10286&frame=3&accno=40739365#x) | - | 1 |
|  | snRNA-associated protein, Sm class^b^ | [*Magnaporthe grisea*/XP_368889](http://200.137.194.69/phorestwww/ncbi.php?accno=38101787) | [9e -32](http://200.137.194.69/phorestwww/xmlparse.php?clone=10279&frame=3&accno=38101787#x) | - | 1 |
|  | U6 snRNA-associated Sm-like protein LSm5^b^ | [*Neurospora crassa*](http://200.137.194.69/phorestwww/ncbi.php?accno=16944624)/CAD11394 | [4e -32](http://200.137.194.69/phorestwww/xmlparse.php?clone=9977&frame=1&accno=16944624#x) | - | 1 |
|  | Ring type Zinc finger protein* | [*Aspergillus nidulans*/XP_411042](http://200.137.194.69/phorestwww/ncbi.php?accno=40739114) | [1e -12](http://200.137.194.69/phorestwww/xmlparse.php?clone=10338&frame=1&accno=40739114#x) | - | 2 |
|  | Zinc finger domain protein* | [*Aspergillus nidulans*/XP_405585](http://200.137.194.69/phorestwww/ncbi.php?accno=40745422) | [3e -14](http://200.137.194.69/phorestwww/xmlparse.php?clone=10222&frame=1&accno=40745422#x) | - | 2 |
|  | tRNA (guanine) methyltransferase^b^ | [*Aspergillus nidulans*/XP_407082](http://200.137.194.69/phorestwww/ncbi.php?accno=40744340) | [3e -23](http://200.137.194.69/phorestwww/xmlparse.php?clone=10818&frame=1&accno=40744340#x) | - | 3 |
|  | [Ap-1-like transcription factor](http://200.137.194.69/phorestwww/ncbi.php?accno=19114574)^b^ | *Aspergillus nidulans*/XP_411679 | [2e -30](http://200.137.194.69/phorestwww/xmlparse.php?clone=10993&frame=3&accno=40742932#x) | - | 1 |
|  | Regulator of nonsense transcripts 1 homolog protein^b^ | *Neurospora crassa*/XP_323582 | [5e -15](http://200.137.194.69/phorestwww/xmlparse.php?clone=10469&frame=-2&accno=32405938#x) | - | 6 |
|  | Arylsulfatase regulatory protein* | *Blastocladiella emersonii*/CO964913 | 1e -138 | - | 11 |
|  | Transcriptional activator protein* | *Coccidioides immitis* **/**EAS34609 | 8e -26 | - | 1 |
|  | Nucleolar protein NOP56^a^ | *Coccidioides immitis* **/**EAS36543 | 8e -84 | 1 | 3 |
|  |  |  |  |  |  |
| **Protein Synthesis** | 40S ribosomal protein S5^a^ | [*Aspergillus nidulans*/XP_404980](http://200.137.194.69/phorestwww/ncbi.php?accno=40746517) | [8e -22](http://200.137.194.69/phorestwww/xmlparse.php?clone=10658&frame=3&accno=40746517#x) | 8 | 7 |
| 40S ribosomal protein S13^b^ | [*Neurospora crassa*/EAA34807](http://200.137.194.69/phorestwww/ncbi.php?accno=32419563) | [2e -37](http://200.137.194.69/phorestwww/xmlparse.php?clone=10367&frame=1&accno=32419563#x) | - | 1 |
|  | 40S ribosomal protein S26^b^ | [*Neurospora crassa*/CAA39162](http://200.137.194.69/phorestwww/ncbi.php?accno=3076) | [3e -52](http://200.137.194.69/phorestwww/xmlparse.php?clone=10870&frame=1&accno=3076#x) | - | 1 |
|  | 60S ribosomal protein L2^b^ | *Coccidioides immitis*/EAS30555 | 9e -54 | - | 1 |
|  | 60S ribosomal protein L3^a^ | [*Neurospora crassa*/CAD70371](http://200.137.194.69/phorestwww/ncbi.php?accno=28881190) | [2e -57](http://200.137.194.69/phorestwww/xmlparse.php?clone=10518&frame=1&accno=28881190#x) | 1 | 2 |
|  | 60S ribosomal protein L20^a^ | [*Magnaporthe grisea*/XP_361110](http://200.137.194.69/phorestwww/ncbi.php?accno=38105659) | [3e -16](http://200.137.194.69/phorestwww/xmlparse.php?clone=10740&frame=2&accno=38105659#x) | 3 | 6 |
|  | 60S Ribosomal protein L27^a^ | [*Aspergillus nidulans*/XP_408359](http://200.137.194.69/phorestwww/ncbi.php?accno=40740131) | [4e -63](http://200.137.194.69/phorestwww/xmlparse.php?clone=10322&frame=2&accno=40740131#x) | 1 | 3 |
|  | 60S ribosomal protein L43B^b^ | [*Ustilago maydis*/XP_400133](http://200.137.194.69/phorestwww/ncbi.php?accno=46098416) | [1e -30](http://200.137.194.69/phorestwww/xmlparse.php?clone=10600&frame=1&accno=46098416#x) | - | 1 |
|  | 60S ribosome subunit biogenesis protein NIP7^b^ | [*Aspergillus fumigatus*](http://200.137.194.69/phorestwww/ncbi.php?accno=28394453)/AAM08680 | [3e -14](http://200.137.194.69/phorestwww/xmlparse.php?clone=10816&frame=3&accno=28394453#x) | - | 1 |
|  | Mitochondrial ribosomal protein S19^b^ | [*Aspergillus nidulans*/XP_404292](http://200.137.194.69/phorestwww/ncbi.php?accno=40746872) | [5e -19](http://200.137.194.69/phorestwww/xmlparse.php?clone=10246&frame=2&accno=40746872#x) | - | 1 |
|  | 14 kDa mitochondrial ribosomal protein* | [*Aspergillus nidulans*/XP_408748](http://200.137.194.69/phorestwww/ncbi.php?accno=40741223) | [4e -46](http://200.137.194.69/phorestwww/xmlparse.php?clone=10336&frame=3&accno=40741223#x) | - | 7 |
|  | Translational machinery component protein^b^ | [*Aspergillus nidulans*/XP_405417](http://200.137.194.69/phorestwww/ncbi.php?accno=40746717) | [1e -19](http://200.137.194.69/phorestwww/xmlparse.php?clone=10837&frame=3&accno=40746717#x) | - | 1 |
|  | Translation initiation factor eIF1 subunit Sui1^a^ | [*Gibberella zeae*/XP_389056](http://200.137.194.69/phorestwww/ncbi.php?accno=42549211) | [2e -36](http://200.137.194.69/phorestwww/xmlparse.php?clone=10370&frame=3&accno=42549211#x) | 2 | 5 |
|  | Translation initiation factor eIF-5A^a^ | *Neurospora crassa*/P38672 | [6e -06](http://200.137.194.69/phorestwww/xmlparse.php?clone=10406&frame=1&accno=729823#x) | 4 | 4 |
|  | Translation initiation factor 3 subunit 2* | *Aspergillus nidulans* **/**XP_660601 | 6e -80 | - | 3 |
|  | Isoleucyl-tRNA synthetase^b^ | [*Aspergillus nidulans*/XP_407499](http://200.137.194.69/phorestwww/ncbi.php?accno=40744150) | [1e -52](http://200.137.194.69/phorestwww/xmlparse.php?clone=10058&frame=2&accno=40744150#x) | - | 2 |
|  | GTP-binding GTP1/OBG-family protein^b^ | [*Aspergillus nidulans*/XP_404829](http://200.137.194.69/phorestwww/ncbi.php?accno=40746312) | [1e -70](http://200.137.194.69/phorestwww/xmlparse.php?clone=10302&frame=2&accno=40746312#x) | - | 1 |
|  |  |  |  |  |  |
| **Protein fate** | Cyclophilin like peptidyl prolyl cis-trans isomerase^b^ | [*Neurospora crassa*](http://200.137.194.69/phorestwww/ncbi.php?accno=18376309)/CAD21421 | [8e -39](http://200.137.194.69/phorestwww/xmlparse.php?clone=10030&frame=3&accno=18376309#x) | - | 1 |
|  | Cyclophilin seven suppressor 1^b^ | [*Aspergillus nidulans*/XP_409575](http://200.137.194.69/phorestwww/ncbi.php?accno=40743408) | [8e -12](http://200.137.194.69/phorestwww/xmlparse.php?clone=10243&frame=1&accno=40743408#x) | - | 2 |
|  | Peptidyl-prolyl cis-trans isomerase-like 4^a^ | *Coccidioides immitis*/ EAS29016 | 1e -46 | 1 | 5 |
|  | Rab geranylgeranyl transferase* | [*Aspergillus nidulans*/XP_412816](http://200.137.194.69/phorestwww/ncbi.php?accno=40740911) | [8e -13](http://200.137.194.69/phorestwww/xmlparse.php?clone=10666&frame=1&accno=40740911#x) | - | 1 |
|  | Protein-L-isoaspartate(D-aspartate)O-methyltransferase^a^ | [*Aspergillus nidulans*/XP_407601](http://200.137.194.69/phorestwww/ncbi.php?accno=40743818) | [5e -55](http://200.137.194.69/phorestwww/xmlparse.php?clone=18843&frame=-1&accno=40743818#x) | 4 | 5 |
|  | COP9 signalosome complex subunit 5^a^ | [*Aspergillus nidulans*/XP_406266](http://200.137.194.69/phorestwww/ncbi.php?accno=40745805) | [1e -35](http://200.137.194.69/phorestwww/xmlparse.php?clone=10063&frame=3&accno=40745805#x) | 1 | 2 |
|  | Guanosine diphosphatase* ^#^ | [*Aspergillus nidulans*/XP_405219](http://200.137.194.69/phorestwww/ncbi.php?accno=40747044) | [2e -15](http://200.137.194.69/phorestwww/xmlparse.php?clone=10356&frame=1&accno=40747044#x) | - | 1 |
|  | Ubiquitin fusion protein^a^ | [*Schizosaccharomyces pombe*/NP_593923](http://200.137.194.69/phorestwww/ncbi.php?accno=19115310) | [8e -67](http://200.137.194.69/phorestwww/xmlparse.php?clone=20004&frame=3&accno=19115310#x) | 3 | 3 |
|  | Ubiquitin thiolesterase otubain-like protein* | [*Aspergillus nidulans*/EAA60354](http://200.137.194.69/phorestwww/ncbi.php?accno=40741164) | [1e -28](http://200.137.194.69/phorestwww/xmlparse.php?clone=10493&frame=3&accno=40741164#x) | - | 1 |
|  | Ubiquitin-conjugating enzyme E2^a^ | [*Gibberella zeae*/XP_388490](http://200.137.194.69/phorestwww/ncbi.php?accno=42547969) | [1e -29](http://200.137.194.69/phorestwww/xmlparse.php?clone=10639&frame=1&accno=42547969#x) | 6 | 7 |
|  | [Ubiquitin/S27a fusion protein](http://200.137.194.69/phorestwww/ncbi.php?accno=11275235)^a^ | [*Aspergillus nidulans*/XP_409009](http://200.137.194.69/phorestwww/ncbi.php?accno=40741760) | [2e -60](http://200.137.194.69/phorestwww/xmlparse.php?clone=10589&frame=3&accno=40741760#x) | 7 | 7 |
|  | Lon protease^b^ | *Oryza sativa/*[AAV59316](http://www.ncbi.nlm.nih.gov/entrez/query.fcgi?cmd=Retrieve&db=Protein&list_uids=55733809&dopt=GenPept) | 1e-05 | - | 1 |
|  | Zinc metalloprotease^b^ | [*Neurospora crassa*](http://200.137.194.69/phorestwww/ncbi.php?accno=18376099)/CAD21161 | [3e -47](http://200.137.194.69/phorestwww/xmlparse.php?clone=10827&frame=1&accno=18376099#x) | - | 1 |
|  | Aspartyl protease^a^ | [*Paracoccidioides brasiliensis*](http://200.137.194.69/phorestwww/ncbi.php?accno=30575834)/AAP32823 | [3e -72](http://200.137.194.69/phorestwww/xmlparse.php?clone=10126&frame=2&accno=30575834#x) | 3 | 7 |
|  | 26S protease subunit protein^b^ | [*Aspergillus nidulans*/XP_411125](http://200.137.194.69/phorestwww/ncbi.php?accno=40742444) | [4e -23](http://200.137.194.69/phorestwww/xmlparse.php?clone=10421&frame=2&accno=40742444#x) | - | 1 |
|  | Non-ATPase regulatory subunit of the 26S proteasome* | [*Aspergillus nidulans*/XP_408912](http://200.137.194.69/phorestwww/ncbi.php?accno=40741155) | [2e -68](http://200.137.194.69/phorestwww/xmlparse.php?clone=10616&frame=2&accno=40741155#x) | - | 1 |
|  | 26S proteasome regulatory subunit rpn12^b^ | [*Aspergillus nidulans*/XP_407156](http://200.137.194.69/phorestwww/ncbi.php?accno=40744414) | [5e -30](http://200.137.194.69/phorestwww/xmlparse.php?clone=10450&frame=3&accno=40744414#x) | - | 1 |
|  | F-box/LRR-repeat protein 7^b^ | [*Aspergillus nidulans*/XP_408647](http://200.137.194.69/phorestwww/ncbi.php?accno=40741663) | [8e -28](http://200.137.194.69/phorestwww/xmlparse.php?clone=10531&frame=3&accno=40741663#x) | - | 3 |
|  | Peptidase C19 subfamily protein^b^ | [*Aspergillus nidulans*/XP_412211](http://200.137.194.69/phorestwww/ncbi.php?accno=40740506) | [7e -08](http://200.137.194.69/phorestwww/xmlparse.php?clone=10809&frame=2&accno=40740506#x) | - | 3 |
|  | Peptidase M28 domain protein* | *Coccidioides immitis*/EAS33583 | 1e -22 | - | 1 |
|  | Alpha-1, 2-galactosyltransferase* ^#^ | [*Aspergillus nidulans*/XP_406106](http://200.137.194.69/phorestwww/ncbi.php?accno=40745978) | [3e -14](http://200.137.194.69/phorestwww/xmlparse.php?clone=10841&frame=1&accno=40745978#x) | - | 1 |
|  | Oligosaccharyltransferase subunit ribophorin^+^ | *Coccidioides immitis*/ EAS29547 | 9e -37 | - | 1 |
|  | Ring (Really Interesting New Gene) type zinc finger (C3HC4) protein^b^ | [*Schizosaccharomyces pombe*/CAB08748.1](http://200.137.194.69/phorestwww/ncbi.php?accno=19114241) | [5e -10](http://200.137.194.69/phorestwww/xmlparse.php?clone=19931&frame=2&accno=19114241#x) | - | 1 |
|  | Tailless Complex Polypeptide 1 chaperonin, subunit epsilon^b^ | *Schizosaccharomyces pombe*/EAA65069 | 6e -16 | - | 2 |
|  | Mannosyltransferase^b^ | [*Paracoccidioides brasiliensis*](http://200.137.194.69/phorestwww/ncbi.php?accno=14161489)/AAK54761 | [3e -70](http://200.137.194.69/phorestwww/xmlparse.php?clone=10220&frame=1&accno=14161489#x) | - | 1 |
|  | Alpha-1, 2-mannosyltransferase^a^ | [*Neurospora crassa*/CAC18268](http://200.137.194.69/phorestwww/ncbi.php?accno=32412802) | [1e -29](http://200.137.194.69/phorestwww/xmlparse.php?clone=10332&frame=1&accno=32412802#x) | 3 | 3 |
|  |  |  |  |  |  |
| **Protein with binding function or cofactor requirement** |  |  |  |  |  |
| RPEL repeat protein^a^ | [*Aspergillus nidulans*/XP_407503.1](http://200.137.194.69/phorestwww/ncbi.php?accno=40744154) | [5e -22](http://200.137.194.69/phorestwww/xmlparse.php?clone=10591&frame=2&accno=40744154#x) | 1 | 3 |
|  |  |  |  |  |
|  |  |  |  |  |  |
| **Transport Facilitation** | Mitochondrial carrier protein^b^ | [*Gibberella zeae*/XP_391004](http://200.137.194.69/phorestwww/ncbi.php?accno=42552339) | [6e -22](http://200.137.194.69/phorestwww/xmlparse.php?clone=10260&frame=1&accno=42552339#x) | - | 3 |
| Xanthine/uracil/vitamin C permease^+^ | [*Magnaporthe grisea*/XP_362769](http://200.137.194.69/phorestwww/ncbi.php?accno=38111793) | [7e -34](http://200.137.194.69/phorestwww/xmlparse.php?clone=10481&frame=1&accno=38111793#x) | - | 1 |
|  | Sugar transporter protein^a^ | [*Gibberella zeae*/XP_381006](http://200.137.194.69/phorestwww/ncbi.php?accno=42547580) | [9e -55](http://200.137.194.69/phorestwww/xmlparse.php?clone=10128&frame=2&accno=42547580#x) | 3 | 5 |
|  | Copper transporter family protein^a^ | [*Gibberella zeae*/XP_380949](http://200.137.194.69/phorestwww/ncbi.php?accno=42547876) | [1e -28](http://200.137.194.69/phorestwww/xmlparse.php?clone=10912&frame=1&accno=42547876#x) | 4 | 6 |
|  | Sulfate permease^b^ | [*Gibberella zeae*/XP_384418](http://200.137.194.69/phorestwww/ncbi.php?accno=42550725) | [4e -46](http://200.137.194.69/phorestwww/xmlparse.php?clone=10823&frame=2&accno=42550725#x) | - | 1 |
|  | Uridine diphosphate N-Acetylglucosamine transporter* ^#^ | [*Neurospora crassa*](http://200.137.194.69/phorestwww/ncbi.php?accno=11359692)/T50997 | [9e -30](http://200.137.194.69/phorestwww/xmlparse.php?clone=10385&frame=3&accno=11359692#x) | - | 1 |
|  | Monossaccharide transport protein^b^ | [*Aspergillus nidulans*/XP_408414](http://200.137.194.69/phorestwww/ncbi.php?accno=40739625) | [8e -55](http://200.137.194.69/phorestwww/xmlparse.php?clone=10362&frame=3&accno=40739625#x) | - | 1 |
|  | Acidic amino acid permease^a^ | [*Aspergillus nidulans*/XP_410255](http://200.137.194.69/phorestwww/ncbi.php?accno=40738903) | [2e -17](http://200.137.194.69/phorestwww/xmlparse.php?clone=9960&frame=1&accno=40738903#x) | 9 | 9 |
|  | B-cell receptor-associated protein 31-like protein+ | [*Aspergillus nidulans*/XP_404956](http://200.137.194.69/phorestwww/ncbi.php?accno=40746493) | [9e -43](http://200.137.194.69/phorestwww/xmlparse.php?clone=10557&frame=2&accno=40746493#x) | - | 1 |
|  | Malate permease^b^ | [*Gibberella zeae*/XP_389995](http://200.137.194.69/phorestwww/ncbi.php?accno=42554838) | [2e -25](http://200.137.194.69/phorestwww/xmlparse.php?clone=9995&frame=1&accno=42554838#x) | - | 2 |
|  | Nuclear pore protein 84/107* | *Coccidioides immitis*/EAS31445 | 2e -13 | - | 1 |
|  | Mitochondrial import receptor subunit Tom20^b^ | [*Aspergillus nidulans*/XP_404696](http://200.137.194.69/phorestwww/ncbi.php?accno=40747502) | [9e -40](http://200.137.194.69/phorestwww/xmlparse.php?clone=10335&frame=3&accno=40747502#x) | - | 1 |
|  | Clathrin adaptor appendage domain protein^b^ | [*Aspergillus nidulans*/XP_408344](http://200.137.194.69/phorestwww/ncbi.php?accno=40740116) | [1e -13](http://200.137.194.69/phorestwww/xmlparse.php?clone=10880&frame=3&accno=40740116#x) | - | 1 |
|  | ER to Golgi transport related protein^b^ | [*Aspergillus nidulans*/XP_412331](http://200.137.194.69/phorestwww/ncbi.php?accno=40739648) | [5e -75](http://200.137.194.69/phorestwww/xmlparse.php?clone=10527&frame=2&accno=40739648#x) | - | 2 |
|  | Vacuolar protein sorting/targeting protein PEP1 precursor^b^ | *Coccidioides immitis*/EAS36959 | 1e -42 | - | 1 |
|  | Regulator of V-ATPase in vacuolar membrane protein* | [*Aspergillus nidulans*/XP_404840](http://200.137.194.69/phorestwww/ncbi.php?accno=40746323) | [9e -59](http://200.137.194.69/phorestwww/xmlparse.php?clone=10272&frame=3&accno=40746323#x) | - | 1 |
|  | Tctex-1 family protein* | [*Aspergillus nidulans*/XP_405470](http://200.137.194.69/phorestwww/ncbi.php?accno=40746360) | [6e -25](http://200.137.194.69/phorestwww/xmlparse.php?clone=10333&frame=2&accno=40746360#x) | - | 2 |
|  | Phosphatidylinositol transfer protein^b^ | [*Aspergillus nidulan*s/XP_410990](http://200.137.194.69/phorestwww/ncbi.php?accno=40739062) | [9e -79](http://200.137.194.69/phorestwww/xmlparse.php?clone=10304&frame=2&accno=40739062#x) | - | 1 |
|  | Importing beta protein^b^ | [*Aspergillus nidulans*/XP_410871](http://200.137.194.69/phorestwww/ncbi.php?accno=40739362) | [6e -71](http://200.137.194.69/phorestwww/xmlparse.php?clone=10298&frame=1&accno=40739362#x) | - | 1 |
|  | Importin beta N-terminal domain protein* | [*Aspergillus nidulans*/XP_410143](http://200.137.194.69/phorestwww/ncbi.php?accno=40738565) | [1e -44](http://200.137.194.69/phorestwww/xmlparse.php?clone=10710&frame=1&accno=40738565#x) | - | 1 |
|  | Phox homology (PX) domain protein^b^ | [*Aspergillus nidulans*/XP_410488](http://200.137.194.69/phorestwww/ncbi.php?accno=40739545) | [3e -06](http://200.137.194.69/phorestwww/xmlparse.php?clone=10536&frame=3&accno=40739545#x) | - | 1 |
|  | Nucleoporin SONB^a^ | *Aspergillus fumigatus*/ XP_751721 | 7e -47 | 1 | 2 |
|  | Exocyst complex component Sec15 protein^a^ | *Coccidioides immitis* **/**EAS37215 | 4e -65 | 1 | 3 |
|  |  |  |  |  |  |
| **Signal Transduction** | [Two-component sensor kinase*](http://200.137.194.69/phorestwww/ncbi.php?accno=50121360) | *Anopheles gambiae*/EAA02130.2 | 2e-38 | - | 6 |
| Histidine protein kinase sensor for GlnG regulator* ^#^ | *Tetrahymena thermophila*/[EAR83219.1](http://www.ncbi.nlm.nih.gov/entrez/query.fcgi?cmd=Retrieve&db=Protein&list_uids=89285199&dopt=GenPept) | 2e-04 | - | 24 |
|  | Protein kinase C conserved region 2^b^ | [*Aspergillus nidulans*/XP_409761.1](http://200.137.194.69/phorestwww/ncbi.php?accno=40743527) | [3e -55](http://200.137.194.69/phorestwww/xmlparse.php?clone=10329&frame=1&accno=40743527#x) | - | 1 |
|  | UVSB Phosphatidylinositol-3 kinase* ^#^ | [*Aspergillus nidulans*/XP_411112.1](http://200.137.194.69/phorestwww/ncbi.php?accno=40742431) | [1e -29](http://200.137.194.69/phorestwww/xmlparse.php?clone=10695&frame=2&accno=40742431#x) | - | 1 |
|  | Serine/threonine-protein kinase SAT4^b^ | [*Aspergillus nidulans* /XP_412967.1](http://200.137.194.69/phorestwww/ncbi.php?accno=40740928) | [2e -51](http://200.137.194.69/phorestwww/xmlparse.php?clone=10766&frame=2&accno=40740928#x) | - | 1 |
|  | Mitogen activated protein kinase^b^ | [*Aspergillus oryzae*](http://200.137.194.69/phorestwww/ncbi.php?accno=45504120)/BAD12561 | [1e -52](http://200.137.194.69/phorestwww/xmlparse.php?clone=10216&frame=3&accno=45504120#x) | - | 1 |
|  | GTPase, G3E family protein^b^ | [*Neurospora crassa*](http://200.137.194.69/phorestwww/ncbi.php?accno=32404938)/XP_323082 | [1e -11](http://200.137.194.69/phorestwww/xmlparse.php?clone=10861&frame=3&accno=32404938#x) | - | 1 |
|  | Rho GTPase activating protein* | [*Aspergillus nidulans*/XP_407883.1](http://200.137.194.69/phorestwww/ncbi.php?accno=40740764) | [3e -49](http://200.137.194.69/phorestwww/xmlparse.php?clone=10241&frame=3&accno=40740764#x) | - | 1 |
|  | GTP binding protein^b^ | [*Neurospora crassa*/CAD70888.1](http://200.137.194.69/phorestwww/ncbi.php?accno=32412930) | [5e -84](http://200.137.194.69/phorestwww/xmlparse.php?clone=10698&frame=2&accno=32412930#x) | - | 1 |
|  | Calcineurin subunit b* | *Neurospora crassa*/P87072 | [1e -77](http://200.137.194.69/phorestwww/xmlparse.php?clone=9976&frame=3&accno=52001480#x) | - | 2 |
|  | Forkhead associated (FHA) protein* | [*Gibberella zeae*/XP_389397.1](http://200.137.194.69/phorestwww/ncbi.php?accno=42554218) | [4e -10](http://200.137.194.69/phorestwww/xmlparse.php?clone=10072&frame=2&accno=42554218#x) | - | 1 |
|  |  |  |  |  |  |
| **Cell Rescue, Defense and Virulence** | Potential secreted Cu/Zn superoxide dismutase^b^ | [*Magnaporthe grisea*/XP_360807](http://200.137.194.69/phorestwww/ncbi.php?accno=38105314) | [8e -11](http://200.137.194.69/phorestwww/xmlparse.php?clone=9973&frame=3&accno=38105314#x) | - | 1 |
| Peroxisomal catalase^a^ | [*Paracoccidioides brasiliensis*/AAL34518](http://200.137.194.69/phorestwww/ncbi.php?accno=30995492) | [4e -66](http://200.137.194.69/phorestwww/xmlparse.php?clone=10382&frame=1&accno=30995492#x) | 3 | 4 |
| Heat shock protein 10, mitochondrial^a^ | [*Gibberella zeae*/XP_386383](http://200.137.194.69/phorestwww/ncbi.php?accno=42551720) | [2e -40](http://200.137.194.69/phorestwww/xmlparse.php?clone=10859&frame=1&accno=42551720#x) | 1 | 3 |
|  | Heat shock protein 60^b^ | *Aspergillus nidulans* **/**XP_659508 | 3e -12 | - | 1 |
|  | Heat shock protein 70^b^ | [*Paracoccidioides brasiliensis*](http://200.137.194.69/phorestwww/ncbi.php?accno=31324921)/AAP05987 | [3e -64](http://200.137.194.69/phorestwww/xmlparse.php?clone=10139&frame=1&accno=31324921#x) | - | 2 |
|  | Heat shock protein 70^b^ | [*Emericella nidulans*](http://200.137.194.69/phorestwww/ncbi.php?accno=2764949)/CAA67431 | [7e -39](http://200.137.194.69/phorestwww/xmlparse.php?clone=10548&frame=2&accno=2764949#x) | - | 1 |
|  | Heat shock protein 90^a^ | *Aspergillus nidulans/*EAA59007 | [4e -38](http://200.137.194.69/phorestwww/xmlparse.php?clone=17239&frame=1&accno=40739817#x) | 5 | 6 |
|  | Hemolysin like protein* ^#^ | [*Aspergillus nidulans*/XP_406013](http://200.137.194.69/phorestwww/ncbi.php?accno=40745885) | [2e -70](http://200.137.194.69/phorestwww/xmlparse.php?clone=10650&frame=2&accno=40745885#x) | - | 1 |
|  |  |  |  |  |  |
| **Biogenesis of cellular components** | Tubulin alpha 1^b^ | [*Aspergillus nidulans*/XP_411707](http://200.137.194.69/phorestwww/ncbi.php?accno=40742960) | [1e -63](http://200.137.194.69/phorestwww/xmlparse.php?clone=10826&frame=1&accno=40742960#x) | - | 1 |
| Tubulin beta-1 chain^b^ | *Aspergillus flavus*/P22012 | [7e -120](http://200.137.194.69/phorestwww/xmlparse.php?clone=16498&frame=1&accno=280475#x) | - | 1 |
| Actin related protein^b^ | [*Aspergillus nidulans*/XP_411146](http://200.137.194.69/phorestwww/ncbi.php?accno=40742465) | [1e -40](http://200.137.194.69/phorestwww/xmlparse.php?clone=10516&frame=1&accno=40742465#x) | - | 1 |
|  | Histone H3^a^ | *Aspergillus fumigatus*/XP_752749 | [5e -67](http://200.137.194.69/phorestwww/xmlparse.php?clone=10455&frame=3&accno=21322637#x) | 11 | 6 |
|  | Histone H2A^a^ | [*Aspergillus nidulans*/XP_412176](http://200.137.194.69/phorestwww/ncbi.php?accno=40740471) | [2e -52](http://200.137.194.69/phorestwww/xmlparse.php?clone=10120&frame=2&accno=40740471#x) | 7 | 6 |
|  |  |  |  |  |  |
| **Cell type differentiation** | Suppressor of anucleate metulae B protein* ^#^ | [*Aspergillus nidulans*/XP_404215.1](http://200.137.194.69/phorestwww/ncbi.php?accno=40746100) | [6e -46](http://200.137.194.69/phorestwww/xmlparse.php?clone=10487&frame=3&accno=40746100#x) | - | 2 |
|  |  |  |  |  |
|  |  |  |  |  |  |
| **Unclassified** | Complex 1 protein (LYR family)* | [*Aspergillus nidulans*/XP_408902](http://200.137.194.69/phorestwww/ncbi.php?accno=40741617) | [8e -32](http://200.137.194.69/phorestwww/xmlparse.php?clone=10681&frame=1&accno=40741617#x) | - | 1 |
|  | Homolog of translationally controlled tumor protein^a^ | [*Aspergillus nidulans*/XP_404778](http://200.137.194.69/phorestwww/ncbi.php?accno=40746028) | [4e -25](http://200.137.194.69/phorestwww/xmlparse.php?clone=10056&frame=3&accno=40746028#x) | 3 | 3 |
|  | YCII related domain protein^b^ | [*Gibberella zeae*/XP_390542](http://200.137.194.69/phorestwww/ncbi.php?accno=42546425) | [2e -23](http://200.137.194.69/phorestwww/xmlparse.php?clone=10633&frame=2&accno=42546425#x) | - | 1 |
|  | Dimeric alpha-beta barrel domain protein^b^ | [*Aspergillus nidulans*/XP_406116](http://200.137.194.69/phorestwww/ncbi.php?accno=40744724) | [1e -23](http://200.137.194.69/phorestwww/xmlparse.php?clone=10891&frame=2&accno=40744724#x) | - | 1 |
|  | Iron-sulfur cluster Isu1-like protein^a^ | [*Gibberella zeae*/XP_382800](http://200.137.194.69/phorestwww/ncbi.php?accno=42547060) | [3e -41](http://200.137.194.69/phorestwww/xmlparse.php?clone=10663&frame=3&accno=42547060#x) | 5 | 4 |

**‡**Transcripts not detected in the mycelium transcriptome (https//dna.biomol.unb.br/Pb) or those induced as detected according to the Audic and Claverie’s method.

^a^ Transcript induced in the transition library compared to the mycelium transcriptome database.

^b^ Transcripts non detected in the mycelia transcriptome (https//dna.biomol.unb.br/Pb).

* Novel genes of *P. brasiliensis*.

^#^ Transcripts confirmed by semi-quantitative RT-PCR.

^+^Genes not described previously for *P. brasiliensis*, isolate *Pb*01, but present in public databases.
