# Supplementary material for: The transcriptome analysis of early morphogenesis in Paracoccidioides brasiliensis mycelium reveals novel and induced genes potentially associated to the dimorphic process
Source: BMC Microbiol. 2007 Apr 10;7:29. doi: 10.1186/1471-2180-7-29 (PMC1855332; doi:10.1186/1471-2180-7-29)
Supplement: Additional File 3 — P. brasiliensis induced transcripts potentially related to signal transduction. Table representing the annotated clusters that were generated by sequencing of the cDNA clones of the transition library. For each cluster the table includes: the function as assigned by BLAST-based similarity, the redundancy in the transition library and in the mycelium transcriptome database. [file 1471-2180-7-29-S3.doc]

**Table 3- *P. brasiliensis* induced transcripts potentially** related to signal transduction.

| **Gene product** | **Putative function** | **Predicted redundancy‡** | |
| --- | --- | --- | --- |
|  |  | **M** | **T** |
| Two component sensor kinase* (yehU) | Stress response, drug sensitivity, sexual development and virulence | - | 6 |
| Histidine protein kinase sensor for GlnG regulator* (glnL) | Defense against nitrogen limitation | - | 24 |
| Protein kinase C conserved region 2 (calB) | Promotes cell-wall stability and increased melanin production | - | 1 |
| UVSB phosphatydilinositol-3-kinase* (uvsB) | Required for S-phase progression and recovery from DNA damage | - | 1 |
| GTP binding protein (ygr) | Cytoskeleton reorganization | - | 1 |
| Serine/threonine-protein kinase SAT4 (sat4) | Involved in cellular potassium uptake | - | 1 |
| Rho GTPase activating protein* (bem3) | Regulator of the beta-(1,3)-glucan synthase | - | 1 |
| Calcineurin subunit b* (canB) | Survival during a variety of stress conditions | - | 2 |
| Mitogen-activated protein kinase (mapkA) | Maintenance of cell integrity | - | 1 |
| Forkhead associated (FHA) protein* (fha) | Play important roles in serine/threonine kinase signaling mechanisms | - | 1 |

‡ The predicted redundancy was obtained on basis of the transition cDNA library and from the mycelia transcriptome database (<https://dna.biomol.unb.br/Pb>/).

* Novel genes detected in *P. brasiliensis*.
